# Efficacy of Off-Label Anti-Amoebic Agents to Suppress Trophozoite Formation of *Acanthamoeba* spp. on Non-Nutrient Agar *Escherichia Coli* Plates

**DOI:** 10.3390/microorganisms10081642

**Published:** 2022-08-13

**Authors:** Vithusan Muthukumar, Lei Shi, Ning Chai, Achim Langenbucher, Sören L. Becker, Berthold Seitz, Erika Orosz, Tanja Stachon, Albrecht F. Kiderlen, Markus Bischoff, Nóra Szentmáry

**Affiliations:** 1 Institute for Medical Microbiology and Hygiene, Saarland University, 66421 Homburg, Germany; 2 Dr. Rolf M. Schwiete Center for Limbal Stem Cell and Aniridia Research, Saarland University, 66421 Homburg, Germany; 3 Division of Life Science and Medicine, University of Science and Technology of China, Hefei 230026, China; 4 Experimental Ophthalmology, Saarland University, 66421 Homburg, Germany; 5 Department of Ophthalmology, Saarland University Medical Center, 66421 Homburg, Germany; 6 Department of Parasitology, National Public Health Center, 1097 Budapest, Hungary; 7 Division for Mycotic and Parasitic Agents and Mycobacteria, Robert Koch Institute, 13353 Berlin, Germany

**Keywords:** *Acanthamoeba* keratitis, off-label anti-amoebic drugs, non-nutrient agar *Escherichia coli* plate assay

## Abstract

*Acanthamoeba* keratitis (AK) is a dangerous infectious disease, which is associated with a high risk of blindness for the infected patient, and for which no standard therapy exists thus far. Patients suffering from AK are thus treated, out of necessity, with an off-label therapy, using drugs designed and indicated for other diseases/purposes. Here, we tested the capability of the off-label anti-amoebic drugs chlorhexidine (CH; 0.1%), dibromopropamidine diisethionate (DD; 0.1%), hexamidine diisethionate (HD; 0.1%), miltefosine (MF; 0.0065%), natamycin (NM; 5%), polyhexamethylene biguanide (PHMB; 0.02%), povidone iodine (PVPI; 1%), and propamidine isethionate (PD; 0.1%) to suppress trophozoite formation of *Acantamoeba castellanii* and *Acanthamoeba hatchetti* cysts on non-nutrient agar *Escherichia coli* plates. Of the eight off-label anti-amoebic drugs tested, only PVPI allowed for a complete suppression of trophozoite formation by drug-challenged cysts for all four *Acanthamoeba* isolates in all five biological replicates. Drugs such as NM, PD, and PHMB repeatedly suppressed trophozoite formation with some, but not all, tested *Acanthamoeba* isolates, while other drugs such as CH, DD, and MF failed to exert a relevant effect on the excystation capacities of the tested *Acanthamoeba* isolates in most, if not all, of our repetitions. Our findings suggest that pre-testing of the AK isolate with the non-nutrient agar *E. coli* plate assay against the anti-amoebic drug intended for treatment should be performed to confirm that the selected drug is cysticidal for the *Acanthamoeba* isolate.

## 1. Introduction

*Acanthamoeba* spp. are microscopic, single-celled living organisms commonly found in the environment in soil, dust, and various water sources (i.e., fresh, brackish, and sea water) that can cause severe illness such as *Acanthamoeba* keratitis (AK), granulomatous amoebic encephalitis (GAE), or disseminated infection [1,2]. The life cycle of *Acanthamoeba* spp. consists of two stages: the actively dividing trophozoite stage, and the cyst, a permanent stage allowing the parasite to better survive hostile conditions. During the trophozoite phase, *Acanthamoebae* divide mitotically and feed on organic matter, bacteria, and other microbes. Under unfavorable conditions, such as a lack of food, extreme temperatures, or a high or low pH value, the trophozoite transforms into a double-walled cyst form, a process called “encystation”. If the conditions become more favorable again, the cyst may convert back into a trophozoite, a process called “excystation” [1,3].

Despite the fact that AK may lead to a complete loss of vision, there is still no specific drug on the market to treat this disease [4]. For this reason, ophthalmologists are forced to use other compounds as off-label anti-amoebic drugs, such as the disinfectant/antiseptic diamidines propamidine isethionate (PD; Brolene), hexamidine diisethionate (HD; Hexacyl), and dibromopropamidine diisethionate (DD; Golden Eye). Other off-label anti-amoebic drugs are the disinfectant/antiseptic biguanides polyhexamethylene biguanide (PHMB; Lavasept) or chlorhexidine (CH; Curasept) (reviewed in [4]). In addition, the antibiotic neomycin, the disinfectant/antiseptic povidone iodine (PVPI), and the anti-leischmanial drug hexadecylphosphocholine (MF; miltefosine) have been described as effective against *Acanthamoeba* spp. isolates. Some authors have also suggested the use of antifungals such as miconazol, clotrimazol, voriconazol, or natamycin (NM) in AK [4].

In case of bacterial keratitis, following corneal smear and culture, the antibiogram helps in finding the appropriate antibiotic medication to treat the keratitis. In contrast, there is neither a standardized treatment regime nor a standardized procedure for AK to test the in vitro susceptibility of the causative agent against the potential off-label drugs, and there is no overall accepted method to define *Acanthamoeba* susceptibility to potential anti-amoebic agents. However, it would be highly valuable for clinicians to define the anti-amoebic susceptibility of the given clinical isolate as soon as possible, so that an aggressive (eye drops hourly), but effective, topical treatment can be introduced at an early stage.

Narasimhan et al. [5] and Kowalski and colleagues [6] described non-nutrient agar *Escherichia coli* plate assays to observe *Acanthamoeba* growth properties under in vitro conditions. In a recent study, we reported that the survival rates of drug-treated trophozoites/cysts determined by enzymatic- and dye-based viability assays might differ substantially from those yielded with the non-nutrient agar *E. coli* plate assay and proposed the latter test system as the gold standard for studying the treatment efficacy of drugs against *Acanthamoeba* spp. isolates [7]. In that study, we also observed that the off-label anti-amoebic drugs PHMB, PD, NM, and PVPI were more effective against the *A. castellanii* strain 1BU than the off-label anti-amoebic drugs CH, HD, DD, and MF under in vitro conditions; however, this was without providing a quantitative evaluation of the observed findings made with the non-nutrient agar *E. coli* plate assay [7]. In the study presented here, we aimed to quantify the effectiveness of the off-label anti-amoebic agents PHMB, CH, HD, PD, DD, NM, MF, and PVPI in killing cysts of the *A. castellanii* strains 1BU (sequence type T4), 3ST (T4), and 9GU (T4), and the *A. hatchetti* strain 11DS (T6), under in vitro conditions. Additionally, we aimed to establish a method which could offer reliable testing of the anti-amoebic susceptibility of *Acanthamoeba* spp. isolates.

## 2. Materials and Methods

### 2.1. Medium and Non-Nutrient Agar Preparation 

Peptone-yeast-glucose medium (PYG), Neff’s constant-pH encystment medium, and Page’s amoeba saline (PAS) were prepared as described in [8]. For the non-nutrient agar, 15 g of agar (Sigma-Aldrich) was mixed with 100 mL PAS and 900 mL distilled water and autoclaved at 121 °C for 15 min.

### 2.2. Acanthamoeba Isolates

The *A. castellanii* strains 1BU and 3ST (both sequence type T4, isolated from corneal specimen of keratitis patients [9]), as well as 9GU (sequence type T4, isolated from the contact lens of a non-AK patient [9]), and the *A. hachetti* strain 11DS (sequence type T6, isolated from a contact lens case of a keratitis patient [9]) were obtained from the Division for Mycotic and Parasitic Agents and Mycobacteria of the Robert Koch Institute, Berlin, Germany.

### 2.3. Acanthamoeba Cultures 

Culturing of 1BU, 3ST, 9GU, or 11DS trophozoites was conducted as described in [8]. Briefly, trophozoites were grown in tissue culture flasks containing 5 mL of PYG broth medium at 30 °C, in an airtight container. Encystment was induced by replacing PYG broth with Neff’s constant-pH encystment medium after trophozoites had reached confluence. After one week of cultivation in Neff’s constant-pH encystment medium at 30 °C, cysts were scraped off and collected by centrifugation for 10 min at 800× *g*. The sedimented cysts were washed once with 5 mL PAS and subsequently resuspended in PAS to a concentration of 3.30 × 10^6^ cysts/mL. This cyst solution was stored at 4 °C until use.

### 2.4. Off-Label Anti-Amoebic Agents and Their Preparation 

For the experiments carried out here, the off-label anti-amoebic agents and concentrations listed in Table 1 were used. 

CH, DD, HD, and MF were obtained as powders, PHMB as a 20% solution, and PVPI as a 7.5% solution. PD was available in a concentration of 0.1% as Brolene^®®^ eye drops, and NM in a concentration of 5% as Natamet^®®^ eye drops. Agents were dissolved or diluted in PBS for treatment of cysts to final concentrations corresponding to the clinically used concentrations [2,10]. Cysts treated with PBS or Lysoform (20%, Rossmann GmbH, Berlin, Germany) served as controls.

### 2.5. Non-Nutrient Agar Escherichia coli Plate Assay

The non-nutrient agar *Escherichia coli* plate assay was carried out as described in [8]. Briefly, cells of *E. coli* strain IM08B [11] were grown overnight on sheep blood agar plates (BD, Heidelberg, Germany) at 35 °C. Fresh grown IM08B colonies were picked with a cotton swab and suspended in PAS equivalent to a McFarland of 4.5. A 100 μL aliquot of this suspension was spread out onto the surface of a non-nutrient agar plate.

**Figure 1:**
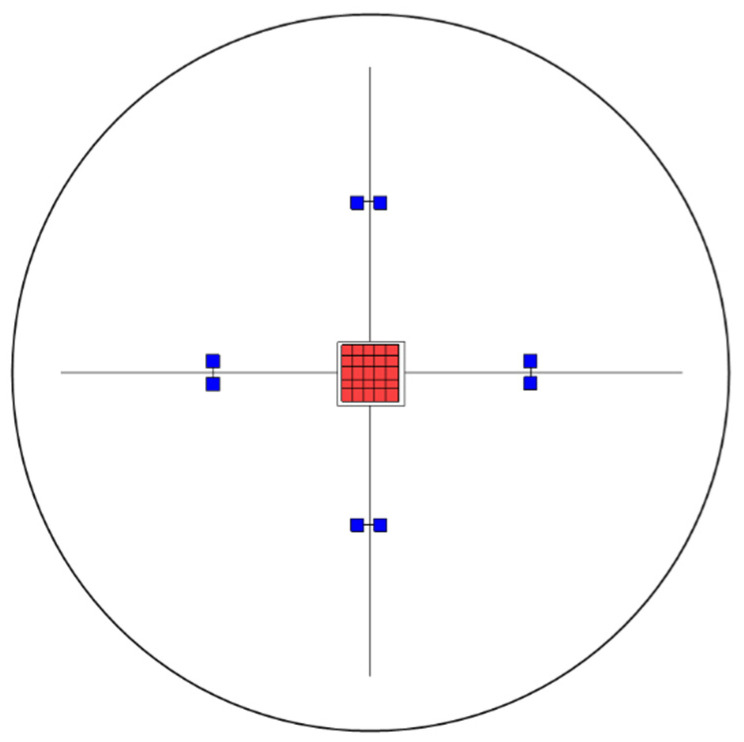
Schematic image of the non-nutrient agar *E. coli* plate (black). Bright-field images were taken with a Leica DMI4000 B microscope after 1 and 3 weeks with a 10-fold objective and 8-fold magnification changer. Twenty-five images were taken from the center (red squares) and eight images from the periphery (blue squares, 1.5 cm distance from the central images and 1.5 mm between the peripheral images along the drawn line) of each plate at each time point.

In the second step, 2 × 10^4^ cysts were mixed with 100 μL PBS in the absence and presence of one of the putative anti-amoebic agents (Table 1) and were incubated for 2 h at 30 °C and 650 rpm to prevent sedimentation. Then, 10 μL of the drug-containing cell suspensions was transferred into fresh tubes and supplemented with 990 μL of PBS (thus diluting the drugs by 100-fold), and the cysts were carefully suspended by pipetting the cell suspension gently up and down. Next, 10 μL of the cell suspensions (~20 cysts) was pipetted onto the middle of the non-nutrient agar *E. coli* plates that were marked on the backside with a 6 × 6 mm^2^ square and crosslines (Figure 1), and the suspension was allowed to desiccate for about 10 min. In the next step, bright-field images of the inoculation regions were taken to determine the exact numbers of cysts that were placed onto the agar plates.

*Acanthamoeba*-inoculated plates were incubated upside up for 24 h at 30 °C. Thereafter, plates were sealed with parafilm (Pechiney, Menasha, WI, USA) and were incubated upside down for up to 3 weeks at 30 °C.

Bright-field images were taken with a Leica DMI4000 B microscope every week for up to 3 weeks with a 10-fold objective and 8-fold magnification changer, which amounts to a total of 80-fold magnification. Twenty-five images were taken from the center (to cover the complete cyst solution spotting area) and eight images from the periphery of each plate at each time point (Figure 1). These experiments (with each off-label anti-amoebic agent) were repeated five times on different days with freshly prepared drugs. All captured images were evaluated manually. Cysts and trophozoites on each picture were counted by the operator. Dark circular structures were identified as cysts, whereas the lighter oval to polygonal structures were identified as trophozoites.

### 2.6. Statistical Analysis

Statistical analysis was performed using GraphPad Prism, version 7.02. Data were analyzed with a Kruskal–Wallis test followed by Dunn’s post hoc test to compare the number of trophozoites in different treatment groups in relation to the PBS control at the analyzed time points. *P* values < 0.05 were considered as statistically significant.

## 3. Results

**Figure 2:**
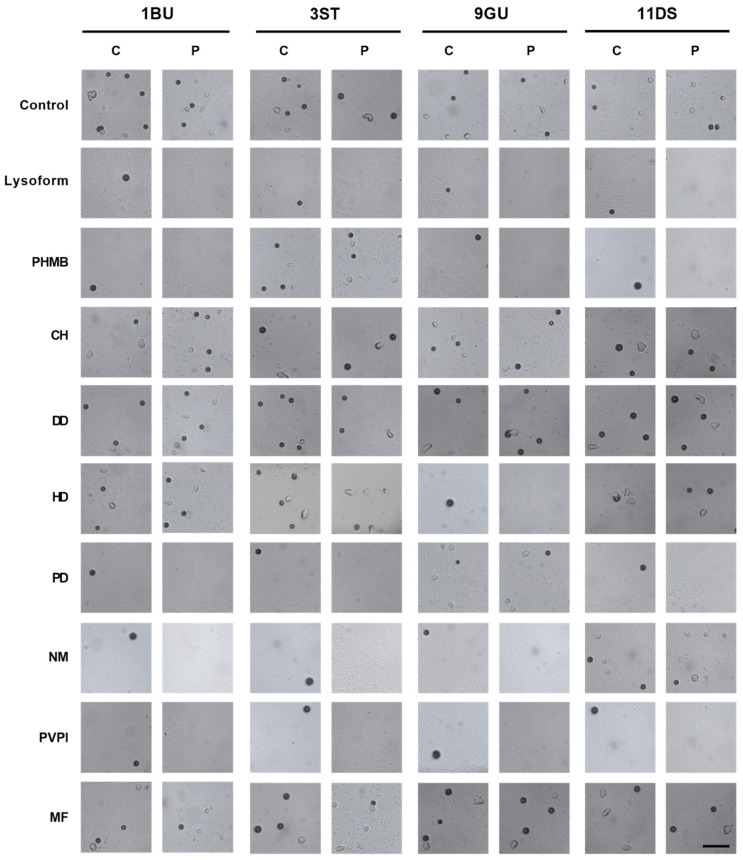
Impact of the drug treatment on the excystment and growth behavior of *A. castellanii* 1BU, 3ST, and 9GU and *A. hatchetti* 11DS cysts on non-nutrient agar *E. coli* plates. Cysts were incubated in PBS medium either in the absence of drugs (control) or with the drugs indicated; afterwards, they were diluted in medium to a cell density of 2000 cysts/mL. Then, 10 μL of the solution (~20 cysts) was pipetted onto the centers of fresh non-nutrient agar *E. coli* plates. Cyst-inoculated plates were subsequently incubated at 30 °C for up to 3 weeks, and images of the central and peripheral regions after 3 weeks of incubation are shown. Images are representative of five independent biological experiments. The disinfectant lysoform served as a killing control. Dark circular structures were identified as cysts, whereas transparent amorphous structures were identified as trophozoites. CH, chlorhexidine; DD, dibromopropamidine diisethionate; HD, hexamidine diisethionate; MF, miltefosine; NM, natamycin; PHMB, polyhexamethylene biguanide; PVPI, povidone iodine; PD, propamidine isethionate; C, central region; P, peripheral region. Scale bar, 100 µm.

Cysts are the more robust life stage of *Acanthamoeba* spp. and are considered less susceptible to drug treatment. In order to prevent a recurrent AK infection, the drug of choice also needs to target the cyst stage of the amoeba [4]. Hence, we tested the efficacy of eight putative anti-amoebic agents (0.02% PHMB, 0.02% CH, 0.1% HD, 0.1% PD, 0.1% DD, 5% NM, 0.0065% MF, 1% PVPI) against the cyst stages of four different *Acanthamoeba* isolates in a single treatment approach (Figure 2).

In our assay, cysts were co-incubated with the drug for 2 h, and defined numbers of drug-treated cysts were subsequently spotted onto the center of *E. coli*-coated non-nutrient agar plates (about 15–20 cysts per plate). Monitoring the central and peripheral regions of the cyst-inoculated plates for up to 3 weeks by light microscopy allowed us to determine whether drug-treated cysts retained their ability to form trophozoites in an excystment-stimulating environment (i.e., on an *E. coli* lawn formed on the surface of a non-nutrient agar plate). Representative images of cysts and trophozoites of the 1BU, 3ST, 9GU, and 11DS isolates on the non-nutrient agar *E. coli* plate at the center and periphery after 3 weeks are displayed in Figure 2 (higher-resolution versions of these images can be found in Supplementary Materials).

**Figure 3:**
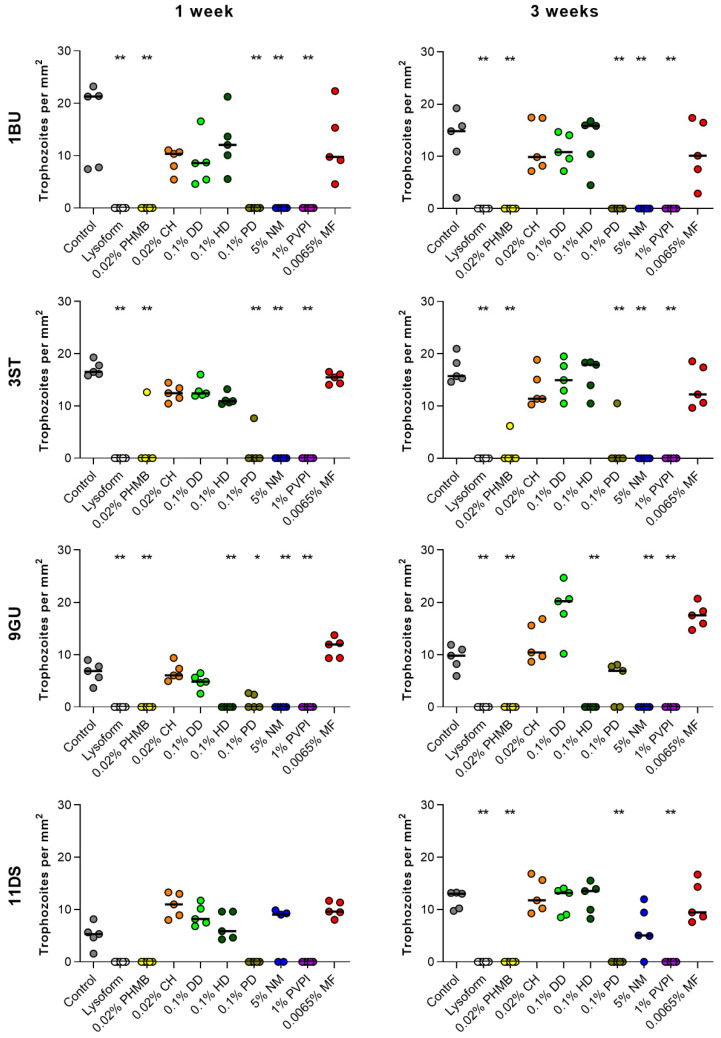
Impact of drug treatment on trophozoite densities of *Acanthamoeba* test strains on non-nutrient agar *E. coli* plates after 1 and 3 weeks. Data represent the median (horizontal line) and the mean trophozoite numbers per mm^2^ per plate of five biological replicates. Statistical differences between drug treatment groups (colored symbols) and the PBS control (gray symbols) were determined by the Kruskal–Wallis test followed by Dunn’s post hoc test. *, *p* < 0.05; **, *p* < 0.01. CH, chlorhexidine (orange); DD, dibromopropamidine diisethionate (mint green); HD, hexamidine diisethionate (dark green); MF, miltefosine (red); NM, natamycin (blue); PHMB, polyhexamethylene biguanide (yellow); PVPI, povidone iodine (purple); PD, propamidine isethionate (moss green).

On all plates, remnants of the original cysts spotted in the central region of the plate remained observable in the same location during the entire follow-up. This location did not change, irrespective of whether an excystation took place or not. However, with the microscopic setup used in this study, discrimination between remnants of cysts releasing a trophozoite and cysts that did not excyst was not possible. Except for the disinfectant Lysoform, which was used as a positive control in this study, only one of the eight drugs, 1% PVPI, allowed for complete suppression of trophozoite formation for all four *Acanthamoeba* test strains in all five repetitions (Figure 3). Other drugs such as 5% NM and 0.02% PHMB effectively suppressed outgrowth of cysts in three out of the four tested *Acanthamoeba* isolates, while 0.1% PD and 0.1% HD repeatedly prevented trophozoite formation for only two and one isolate, respectively. The anti-amoebic drug candidates 0.02% CH, 0.1% DD, and 0.0065% MF, on the other hand, failed to suppress the excystation of drug-challenged cysts on the *E. coli* lawn in all cases (Figure 3). Notably, the anti-amoebic drug candidates that allowed the suppression of trophozoite formation with at least two out of the four *Acanthamoeba* test strains, failed to be effective with different isolates. While 5% NM treatment of cysts still allowed for trophozoite formation by drug-treated *A. hatchettii* 11DS cysts in four out of five repetitions, excystation for all three *A. castellanii* test strains (i.e., 1BU, 3ST, 9GU) was effectively prevented by this drug. The diamidine PD at a concentration of 0.1% suppressed trophozoite formation of *A. castellanii* isolate 1BU and of *A. hatchettii* strain 11DS, but failed to prevent trophozoite formation of drug-challenged *A. castellanii* 3ST and 9GU cysts in one and three out of five repetitions, respectively. The biguanide PHMB, at a concentration of 0.02%, suppressed trophozoite formation of drug-challenged *A. castellanii* isolates 1BU and 9GU, and of *A. hatchettii* strain 11DS, but failed to do so for drug-challenged *A. castellanii* 3ST cysts in one out of five repetitions. Comparing the number of plates featuring trophozoites anywhere on the plate at a given time revealed a consistency in trophozoite-negative/positive plates in 48 out of 50 experiments. Only in two experiments were trophozoites detected after three weeks of incubation, but not after one week, suggesting that an observation period of one week is already sufficient to predict whether the anti-amoebic drug candidate chosen for treatment is cysticidal for the Acanthamoeba isolate intended to be killed.

## 4. Discussion

AK is an infectious disease, which is associated with a high risk of blindness for the infected patient, and thus far, no standard therapy exists. Patients suffering from AK are usually treated with an off-label therapy with drugs designed and indicated for other diseases, for example, the disinfectant PD, which is used in ophthalmology as a treatment option for minor bacterial or fungal infections of the eye or eyelid [12]. Another example is MF, which was originally developed as a cancer medication but is currently used as a treatment option against cutaneous leishmaniasis in humans and animals [13].

Several clinical reports indicate that the drugs PD and MF have also succeeded in treating individual AK cases [12]. However, information on whether these drugs work on different *Acanthamoeba* spp. isolates is usually missing [9]. In order to fill this gap, this study examined the in vitro activity of several drugs suggested as off-label therapy options for AK against four different *Acanthamoeba* spp. isolates of sequence types T4 and T6. In the process, the focus was on cysts, the dormant stage of this amoeba, which are usually less susceptible to drug treatment than the actively dividing trophozoites [8,14]. We treated the cyst suspension with each drug for 2 h, then diluted the cysts and drug suspension by 100-fold, and placed an aliquot of the diluted cyst/drug mixture onto the center of a non-nutrient agar *E. coli* plate, thereby transferring lower concentrations of the drug onto the test plate. This method differs from the in vitro drug treatment procedure reported in other studies [5,6], in which the drug was completely withdrawn from the cysts after the incubation step and before the drug-challenged cysts were seeded in/onto the growth medium. However, we found it important to observe the cyst behavior under the conditions described here, as in vivo, the anti-amoebic drugs are also continuously in contact with the pathogen while being diluted in human tear fluid and the deepithelialized corneal tissue over time. We found that, under our test conditions, only one of the suggested AK drug candidates, the disinfectant/antiseptic PVPI, effectively prevented the excystment and formation of trophozoites for a period of at least 3 weeks (suggesting a cysticidal efficacy of 100%) on all four *Acanthamoeba* spp. isolates. Most of the AK drug candidates, on the other hand, worked effectively on cysts of some, but not all, *Acanthamoeba* spp. isolates tested in this study. However, the disinfectants/antiseptics CH and DD and the anti-leischmanial drug MF failed to exert a relevant killing capacity against cysts of all four tested *Acanthamoeba* spp. isolates in our in vitro test system (i.e., pre-treating cysts with the drug for 2 h, and culturing the drug-treated cysts on non-nutrient agar *E. coli* plates for up to 3 weeks). Our findings for CH and MF are in contrast to several in vitro and in vivo studies reporting promising cysticidal activities of these compounds against *Acanthamoeba* spp. isolates [5,10,15,16,17,18,19,20]. Travis K. Redd and colleagues [16] recently reported minimum cysticidal concentrations (MCCs) for CH between 3.1 µg/mL and 25 µg/mL when testing the cysticidal activity of the disinfectant on nine human AK isolates, which are about 8- to 30-fold lower than the CH concentration used in our assays (0.02% = 200 µg/mL). Narasimhan and colleagues [5] reported MCCs for CH in the range of 1.6 µg/mL to 100 µg/mL for 19 AK isolates when the compound was co-cultured with the cysts for 48 h. A promising in vivo activity of CH was reported by Kosrirukvongs and colleagues [18] on AK isolates when the drug was applied hourly for one month followed by four times a day for up to nine months. The anti-leischmanial drug MF was reported to display a significant cysticidal activity on *Acanthamoeba* spp. in vitro, although MF failed to completely prevent trophozoite formation in this study, even if the drug was co-cultured with the cysts for 72 h [19]. A more recent study identified MCCs for MF in the range of 0.001% to 0.0013% for five AK isolates and reported a promising in vivo activity of topical MF as monotherapy in the treatment of AK [20]. Satisfactory in vivo activity of MF against AK was also reported by Thulasi and colleagues [17] in a clinical case study, in which the drug was applied perorally. The discrepancy between our observations and the finding listed above might be, at least in part, explained by the fact that in our in vitro test system, cysts were in contact with the drug for two hours only, whereas in the other in vitro studies and in the in vivo setting, the residence time of the drug on cysts was much longer. Support for this hypothesis is given by earlier findings made by Sunada and colleagues [10], who reported a 100% cysticidal effect for 0.02% chlorhexidine gluconate on AK isolates if the cysts were exposed to the drug for 24 h, while trophozoite formation was observed in all repetitions when the cysts were co-cultured with the drug for one hour only.

We also observed that some of the off-label AK drug candidates were highly effective (no trophozoites formed within 3 weeks post-treatment) in some of our biological replications but failed to kill all cysts in the other replications for reasons which are still unknown. The latter observation indicates that false positive or false negative results might occur if the in vitro drug efficacy is tested only once with the non-nutrient agar *E. coli* plate assay. However, in the vast majority of our repetitions (48/50), the efficacy of the drug against the *Acanthamoeba* spp. isolate was already reliably assessable after one week post-treatment. Thus, we propose that the start of an empirical treatment of AK should be accompanied by an in vitro-based drug efficacy study on cysts, which should be carried out in at least two independent experiments. Furthermore, an observation period of one week is usually sufficient to assess the treatment efficacy. This testing procedure of the in vitro susceptibility of the AK isolate with the non-nutrient agar *E. coli* plate assay against the intended anti-amoebic drug is likely to offer valuable information at an early stage of the treatment regime in terms of whether the drug is likely to be active on the *Acanthamoeba* isolate in vivo.

Study limitations: in our study, only a small number of *Acanthamoeba* isolates were tested with only one drug concentration by co-culturing cysts and the drug for 2 h. Thus, we cannot exclude that some of the anti-amoebic drug candidates such as 0.02% CH, 0.1% DD, and 0.0065% MF, which failed to be cysticidal in our study, might be highly effective against other AK isolates, while 1% PVPI, which was cysticidal for all four *Acanthamoeba* isolates tested here, might fail to exert a cysticidal effect on other AK isolates. Similarly, higher concentrations of the drugs and/or longer co-incubation intervals might render drugs more effective. As earlier observations also indicated that a good in vitro activity of a certain anti-amoebic drug candidate might not necessarily correlate with the clinical outcomes of AK [14], we also cannot exclude that pre-testing of the in vitro susceptibility of the AK isolate with the non-nutrient agar *E. coli* plate assay against the anti-amoebic drug intended for treatment might indicate a susceptibility of the AK isolate that is not seen in vivo.

## 5. Conclusions

Our findings strongly suggest that an empirical treatment of an AK patient with any of the AK drug candidates studied here should be accompanied by an in vitro activity testing of the AK isolate against the chosen drug. This test can determine whether the off-label drug is also highly effective against the cyst stage of this isolate. Such a procedure could reduce the risk of recurrent AK infections, since theoretically, even one surviving cyst could induce a new episode of AK in the absence of treatment. Monitoring trophozoite formation of drug-challenged *Acanthamoeba* spp. cysts on the non-nutrient agar *E. coli* plate for 1 week in experiments and repeated at least twice is sufficient to reliably inform us about the efficacy of the tested drugs to prevent excystation. In our opinion, this is an important measure to estimate the overall susceptibility of the given clinical isolate to the anti-amoebic drugs considered for treatment.

## Figures and Tables

**Table 1:** Off-label anti-amoebic agents, drug concentrations used in experiments, and sources.

Drug Name	Abbreviation	Final Concentration	Source *
Chlorhexidine	CH	0.02%	Sigma-Aldrich, USA
Dibromopropamidine-diisethionat	DD	0.1%	EP, Strasbourg, France
Hexamidine-diisethionate	HD	0.1%	EP, Strasbourg, France
Miltefosine	MF	0.0065%	Sigma-Aldrich, USA
Natamycin	NM	5%	Alcon laboratories, Fort Worth, USA
Propamidine-isethionate	PD	0.1%	Patheon UK Ltd., Swindon, UK
Polyhexamethylene biguanide	PHMB	0.02%	PSMU, Homburg/Saar, Germany
Povidone-iodine	PVPI	1%	B. Braun, Melsungen, Germany

* EP, European Pharmacopoeia; PSMU, Pharmacy of Saarland Medical University.

## Data Availability

The datasets generated and analyzed during the current study are available from the corresponding author on reasonable request.

## Supplementary Materials

The following are available online at https://www.mdpi.com/article/10.3390/microorganisms10081642/s1; Figures S1–S80, Original jpeg images of Figure 2.
