# Supplementary figures and images for: Efficacy of Off-Label Anti-Amoebic Agents to Suppress Trophozoite Formation of Acanthamoeba spp. on Non-Nutrient Agar Escherichia Coli Plates
Source: Microorganisms. 2022 Aug 13;10(8):1642. doi: 10.3390/microorganisms10081642 (PMC9412465; doi:10.3390/microorganisms10081642)

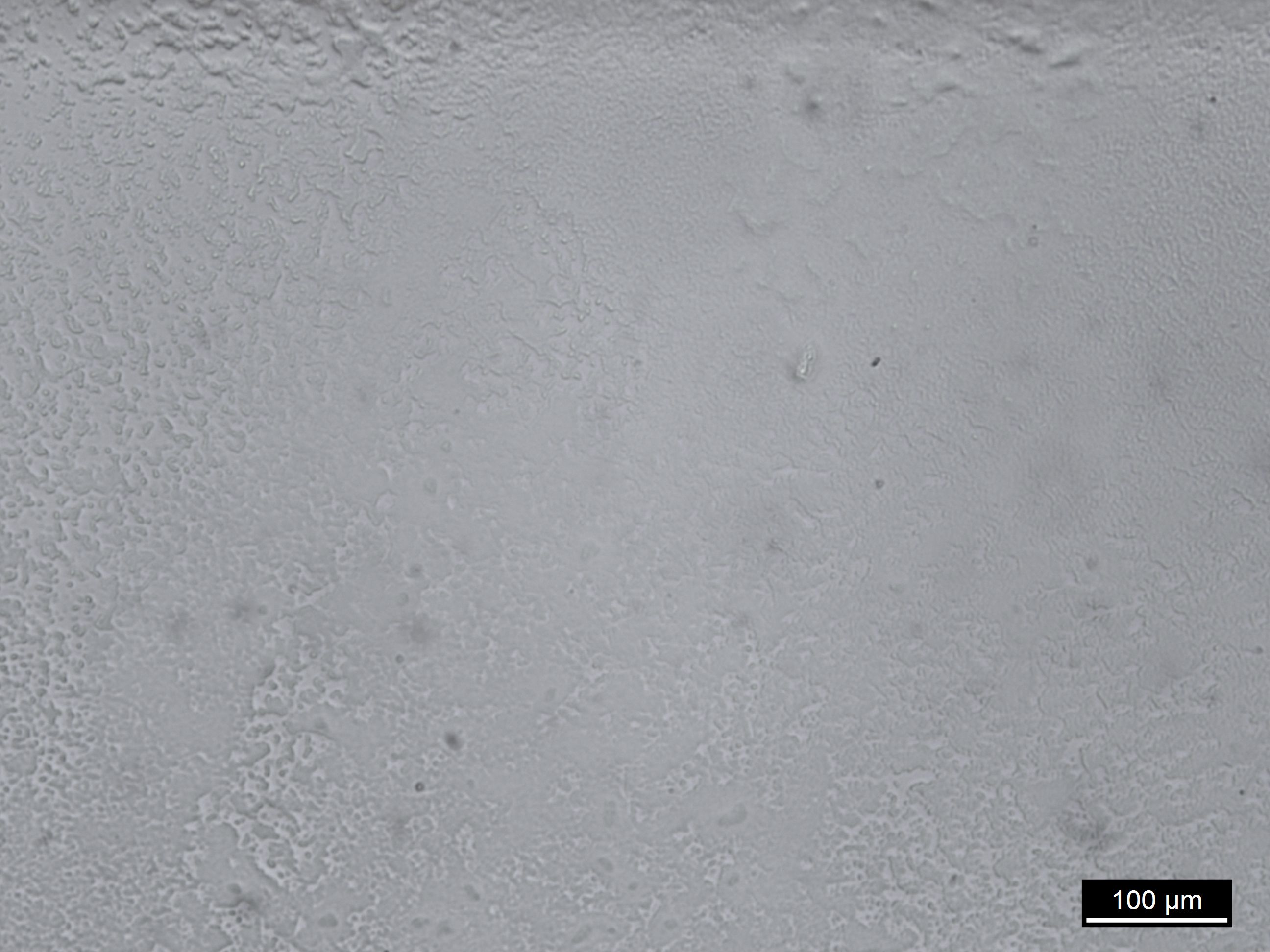

Supplement: Supplementary file 1 [file microorganisms-10-01642-s001.zip › S10_IBU_Lysoform_P.jpg]

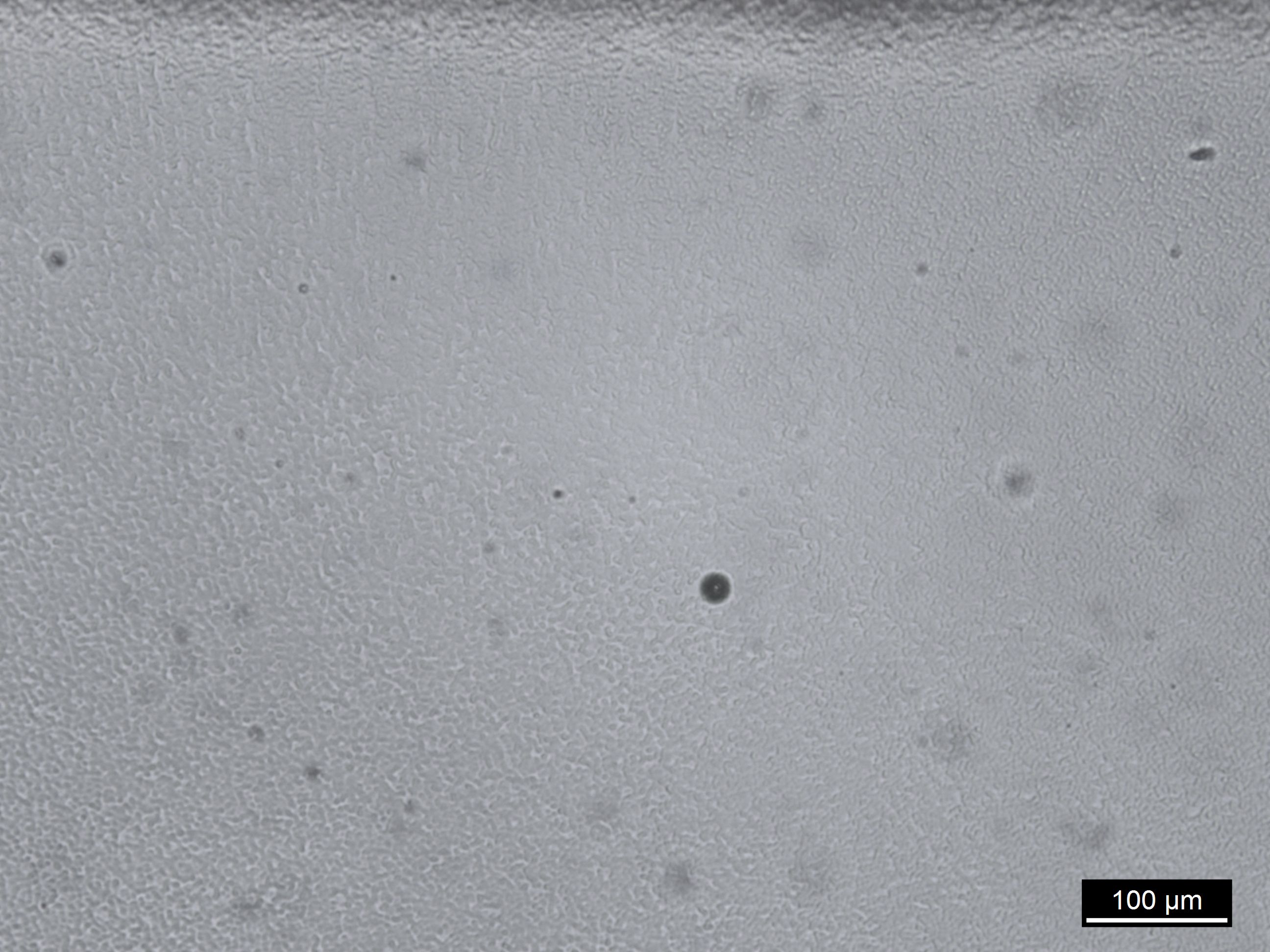

Supplement: Supplementary file 1 [file microorganisms-10-01642-s001.zip › S11_3ST_Lysoform_C.jpg]

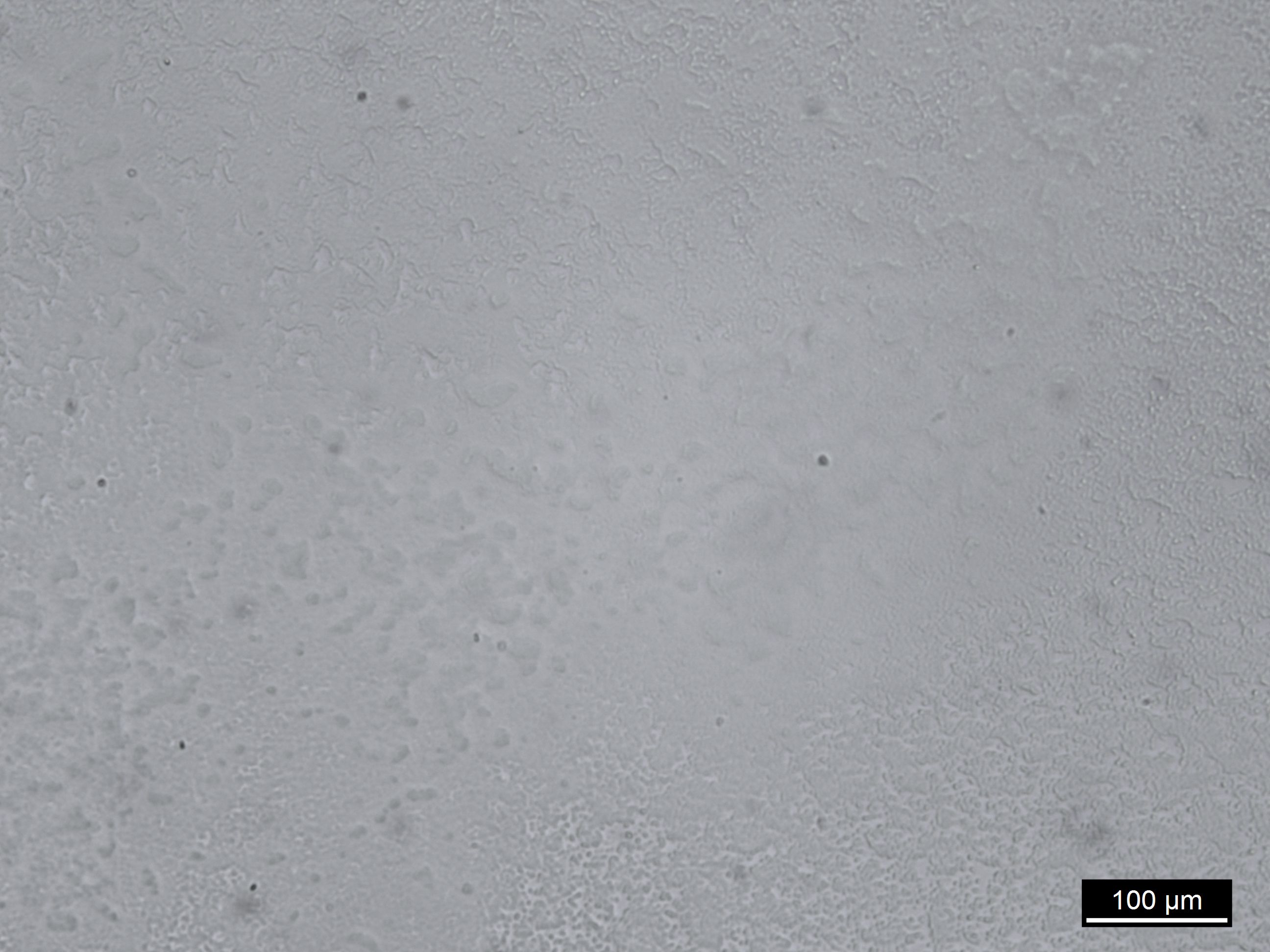

Supplement: Supplementary file 1 [file microorganisms-10-01642-s001.zip › S12_3ST_Lysoform_P.jpg]

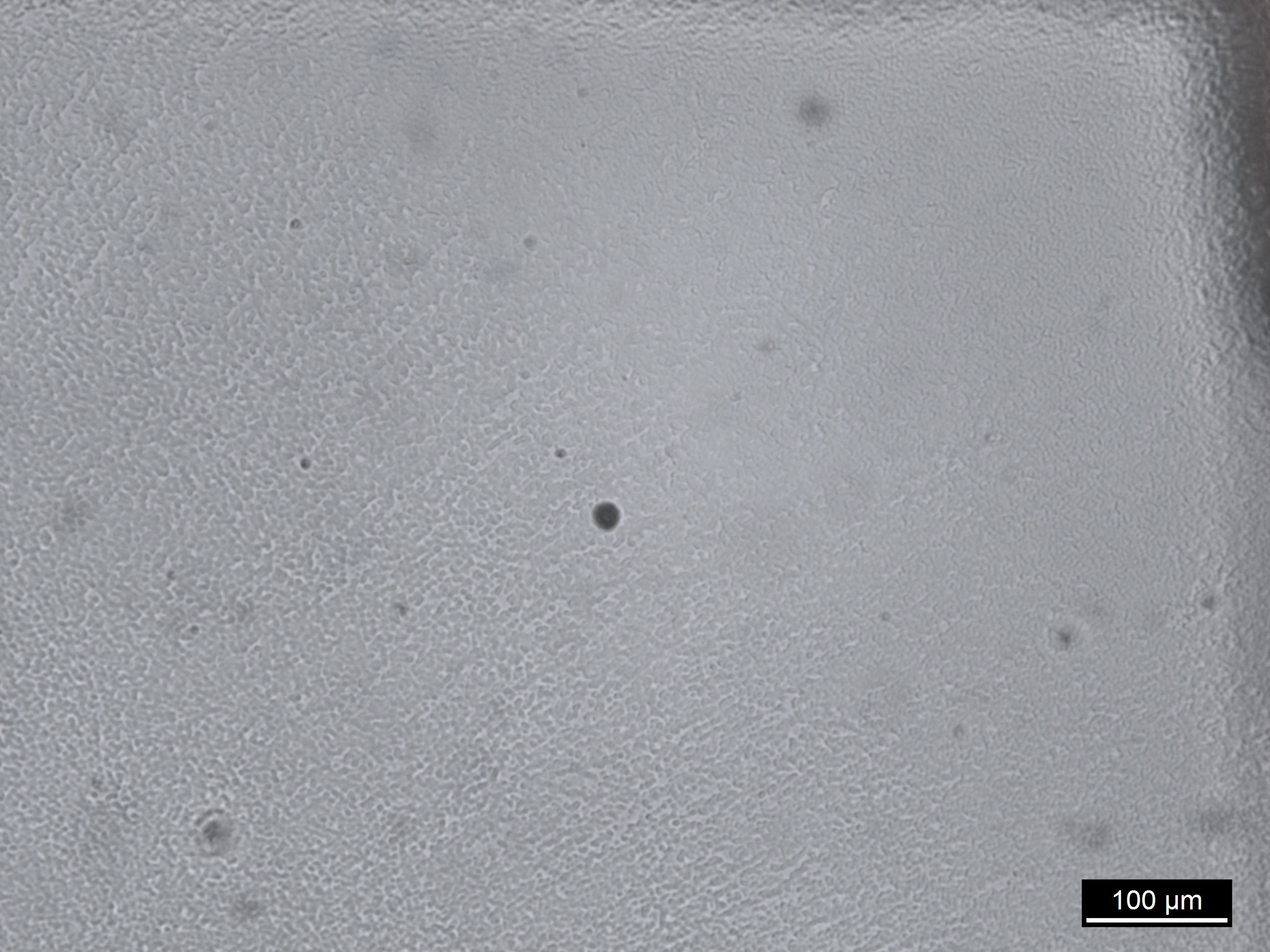

Supplement: Supplementary file 1 [file microorganisms-10-01642-s001.zip › S13_9GU_Lysoform_C.jpg]

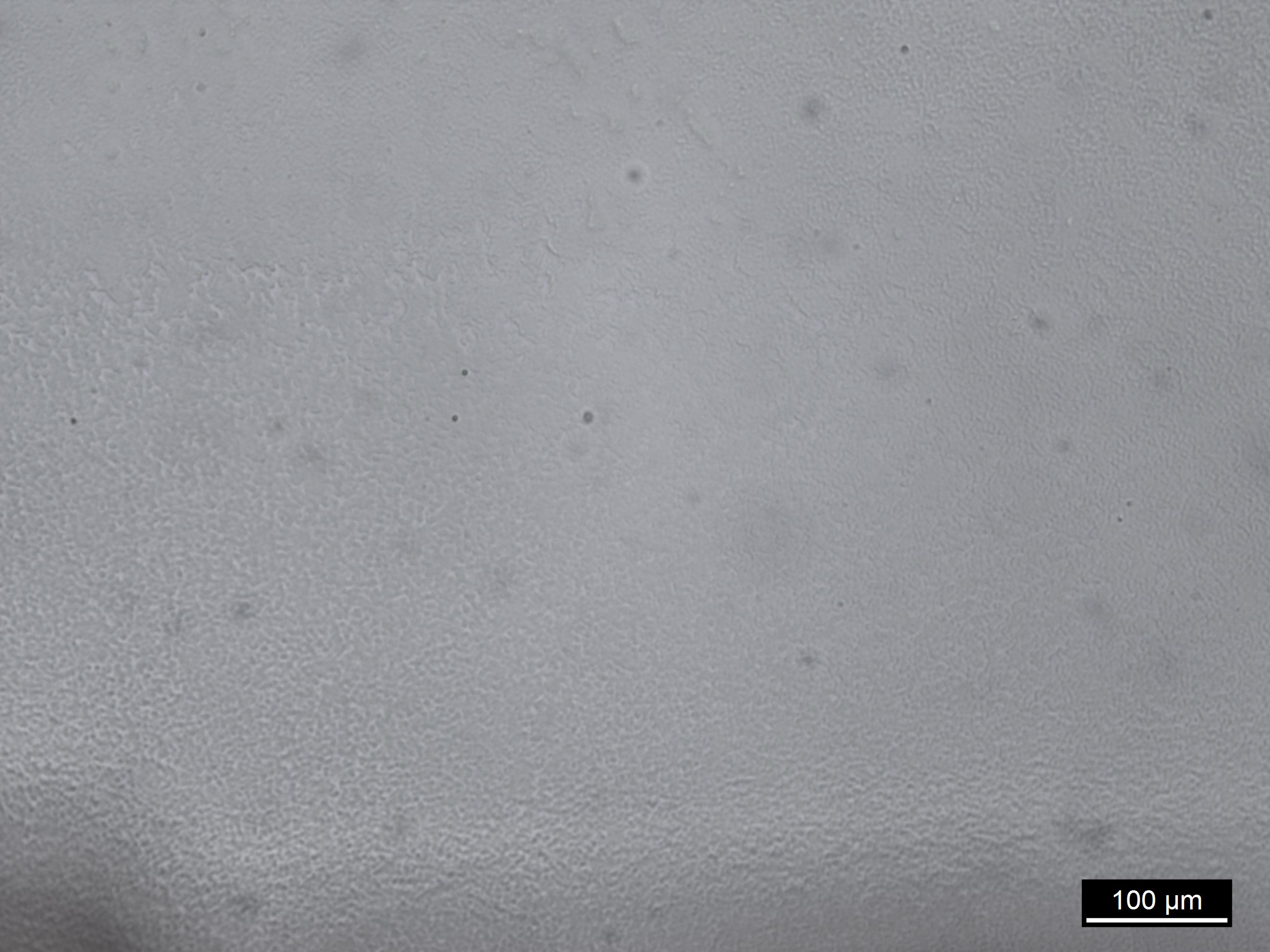

Supplement: Supplementary file 1 [file microorganisms-10-01642-s001.zip › S14_9GU_Lysoform_P.jpg]

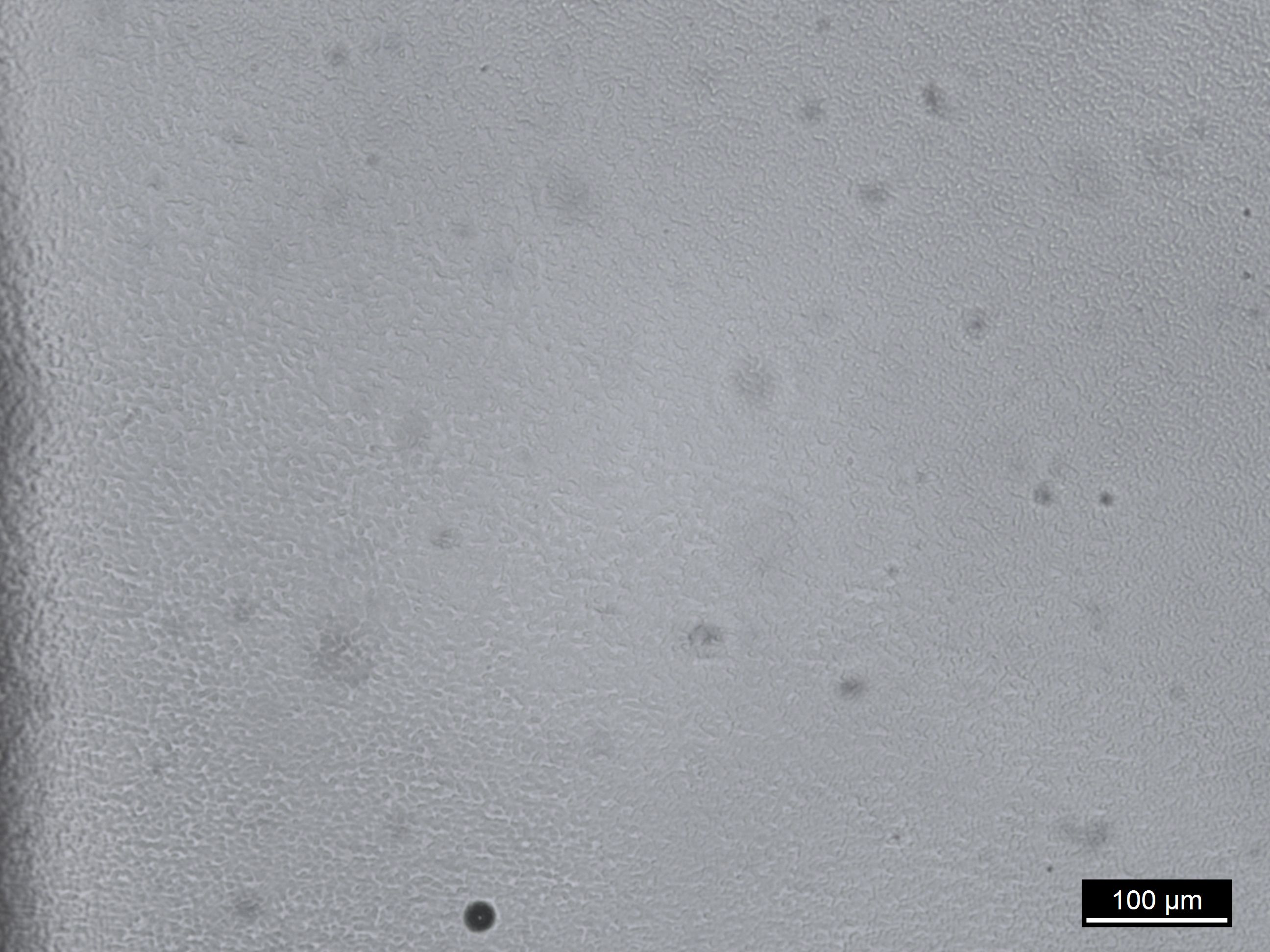

Supplement: Supplementary file 1 [file microorganisms-10-01642-s001.zip › S15_11DS_Lysoform_C.jpg]

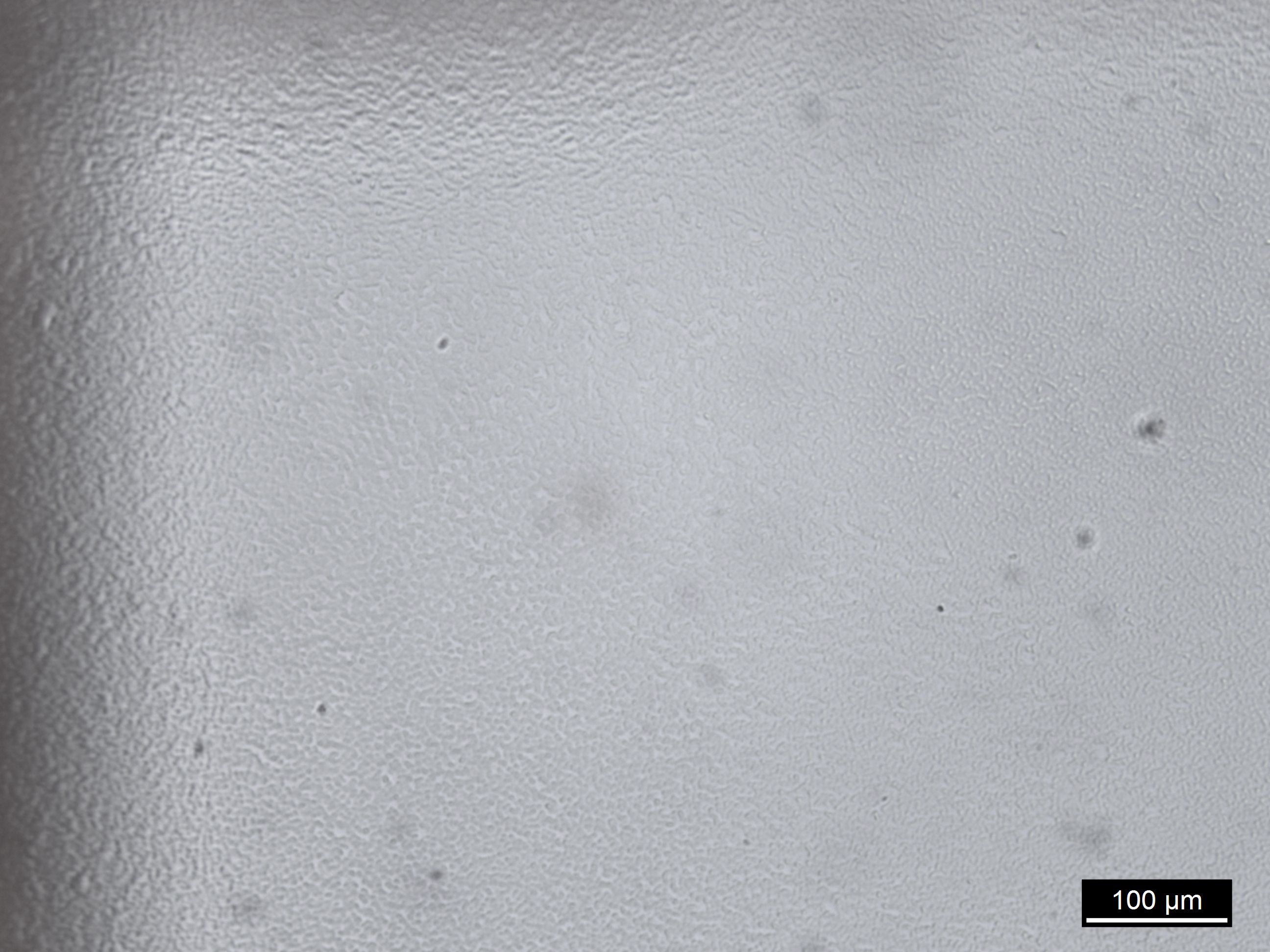

Supplement: Supplementary file 1 [file microorganisms-10-01642-s001.zip › S16_11DS_Lysoform_P.jpg]

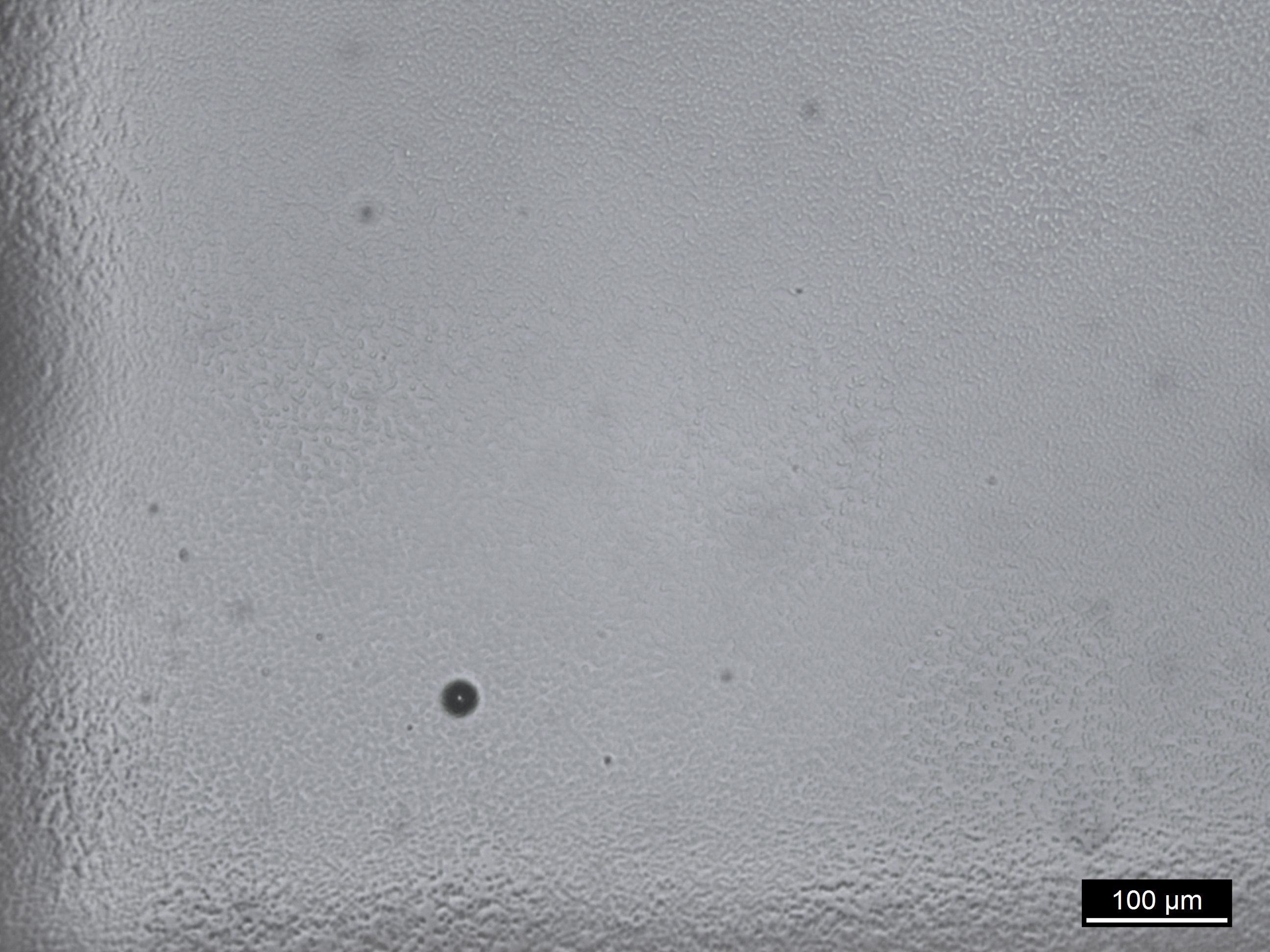

Supplement: Supplementary file 1 [file microorganisms-10-01642-s001.zip › S17_IBU_PHMB_C.jpg]

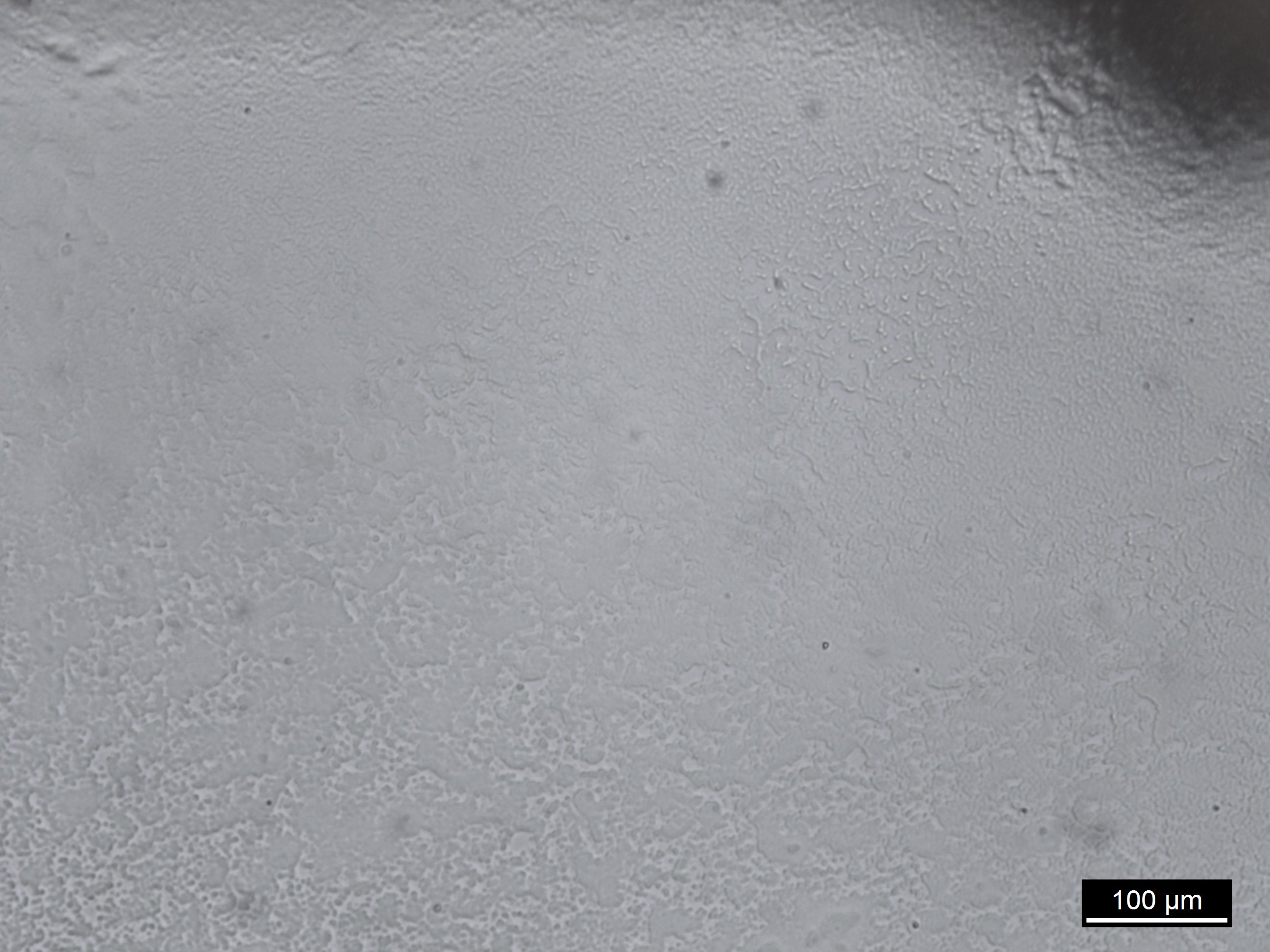

Supplement: Supplementary file 1 [file microorganisms-10-01642-s001.zip › S18_IBU_PHMB_P.jpg]

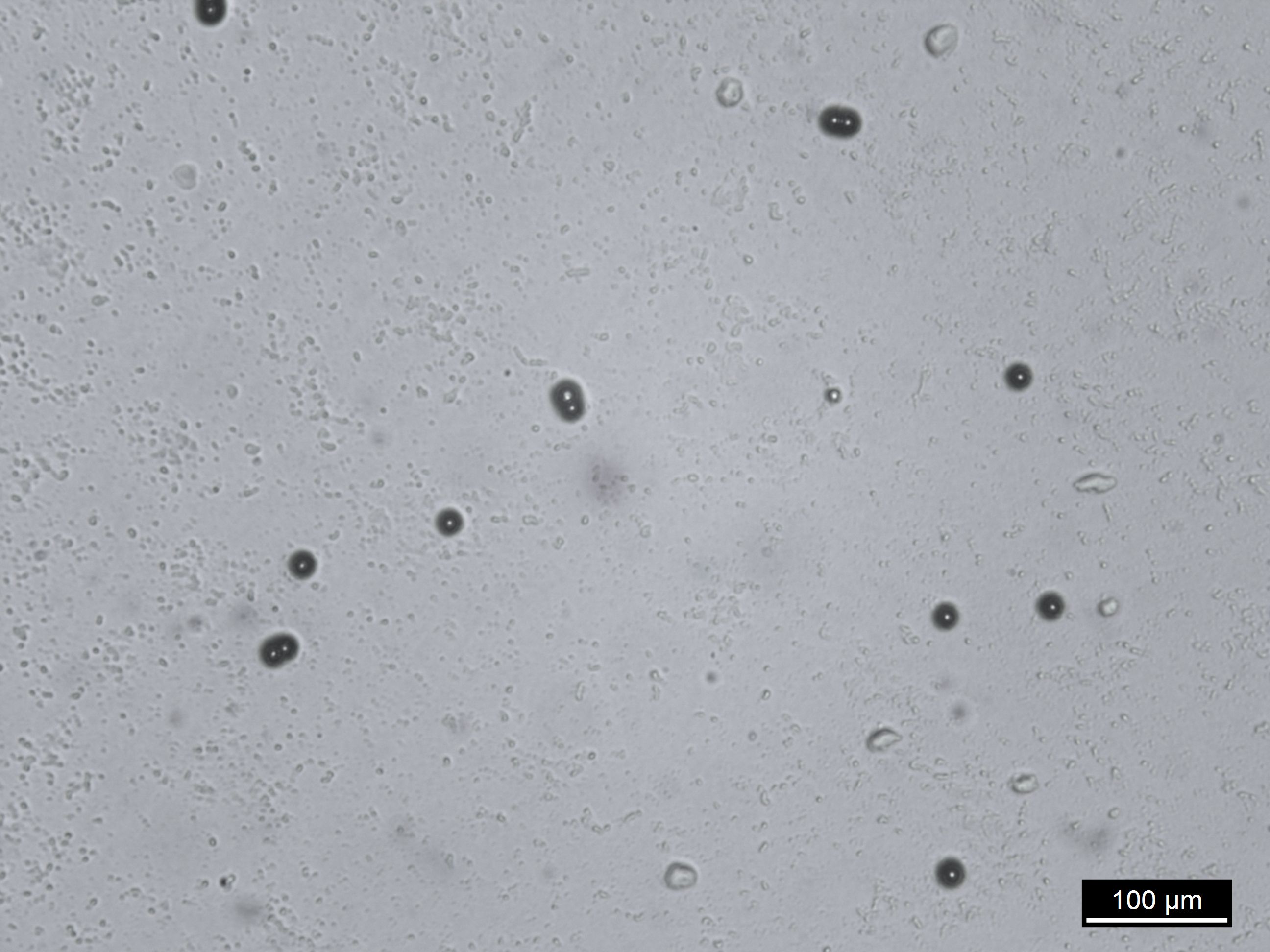

Supplement: Supplementary file 1 [file microorganisms-10-01642-s001.zip › S19_3ST_PHMB_C.jpg]

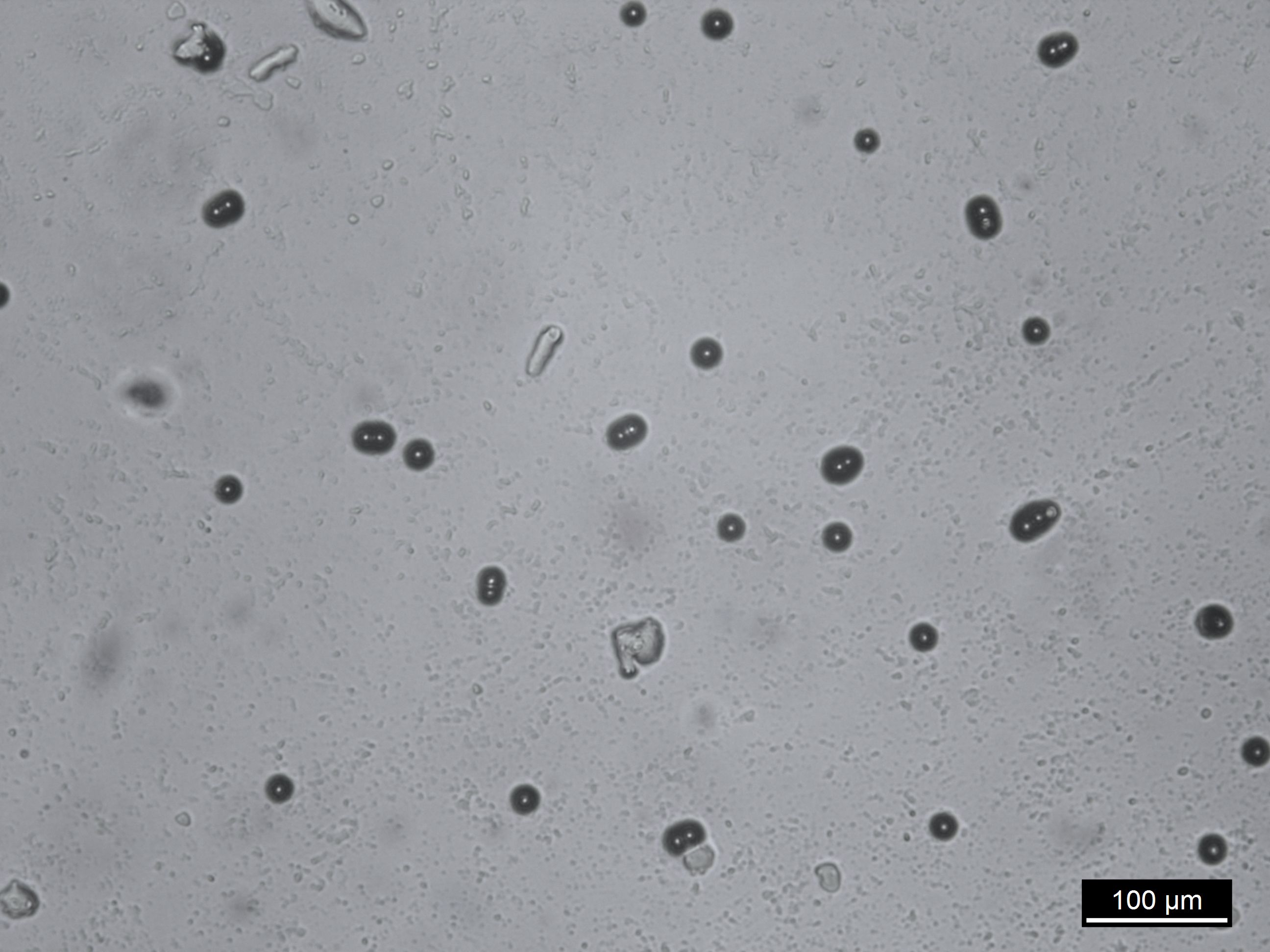

Supplement: Supplementary file 1 [file microorganisms-10-01642-s001.zip › S1_IBU_Control_C.jpg]

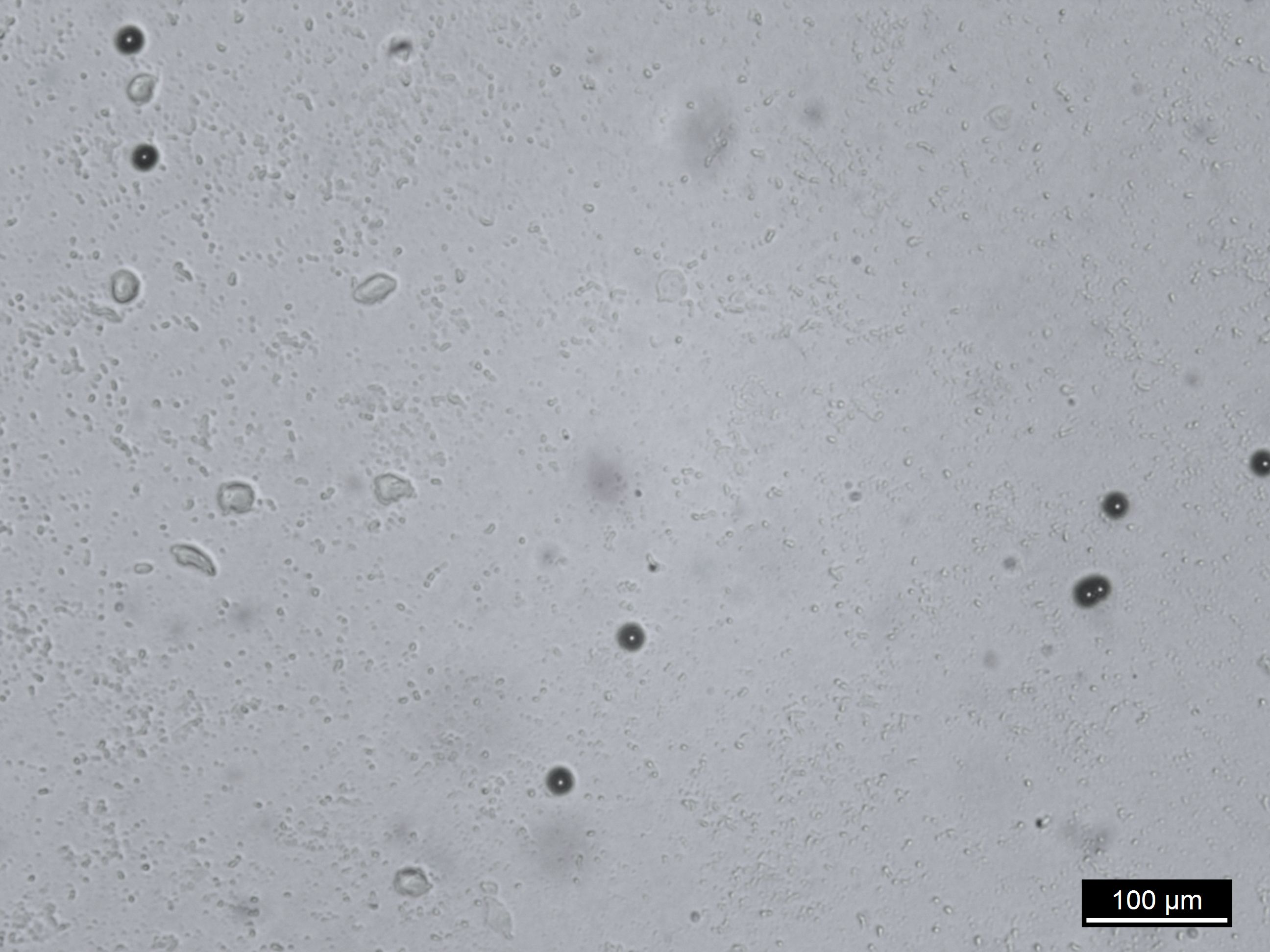

Supplement: Supplementary file 1 [file microorganisms-10-01642-s001.zip › S20_3ST_PHMB_P.jpg]

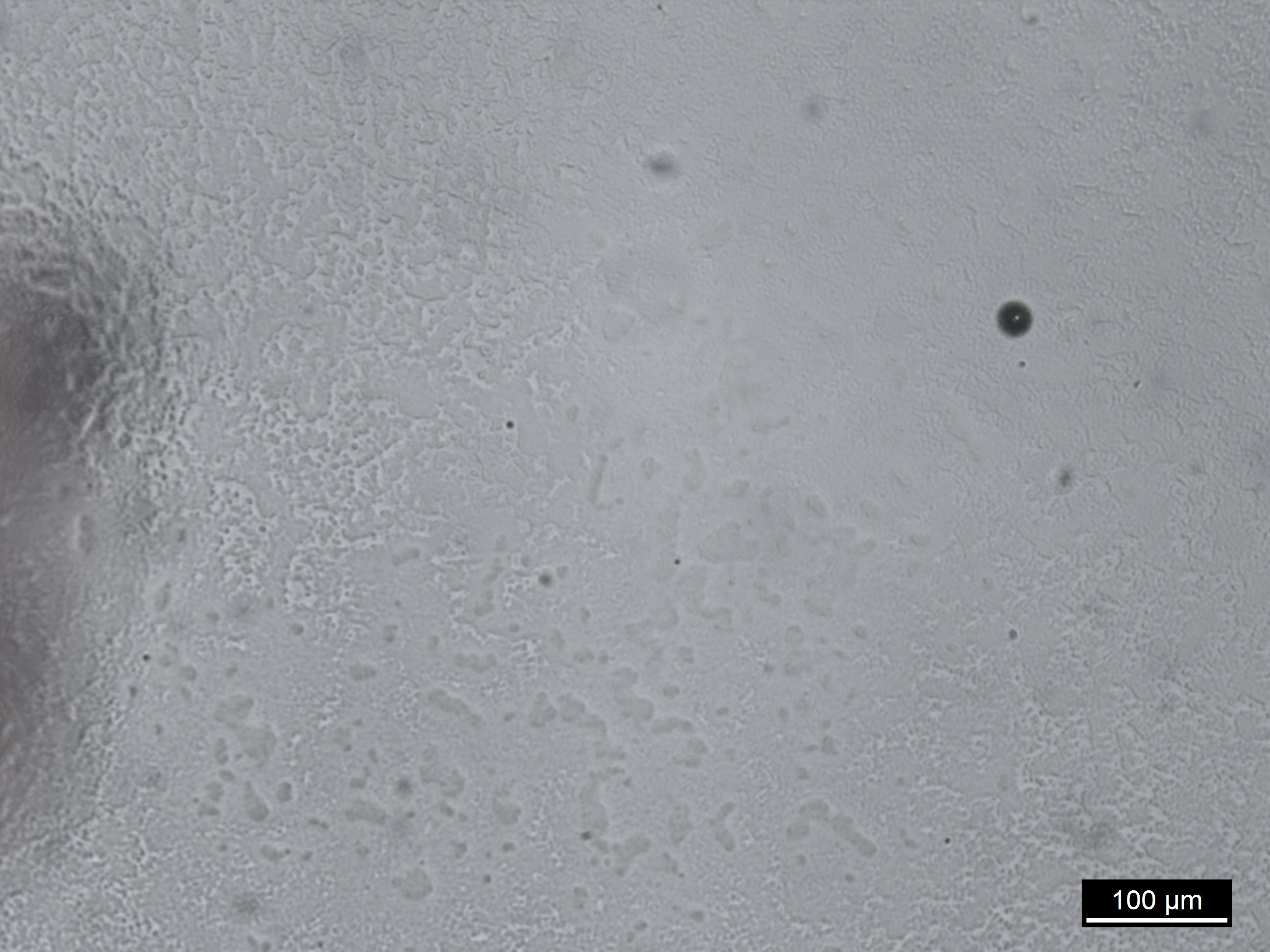

Supplement: Supplementary file 1 [file microorganisms-10-01642-s001.zip › S21_9GU_PHMB_C.jpg]

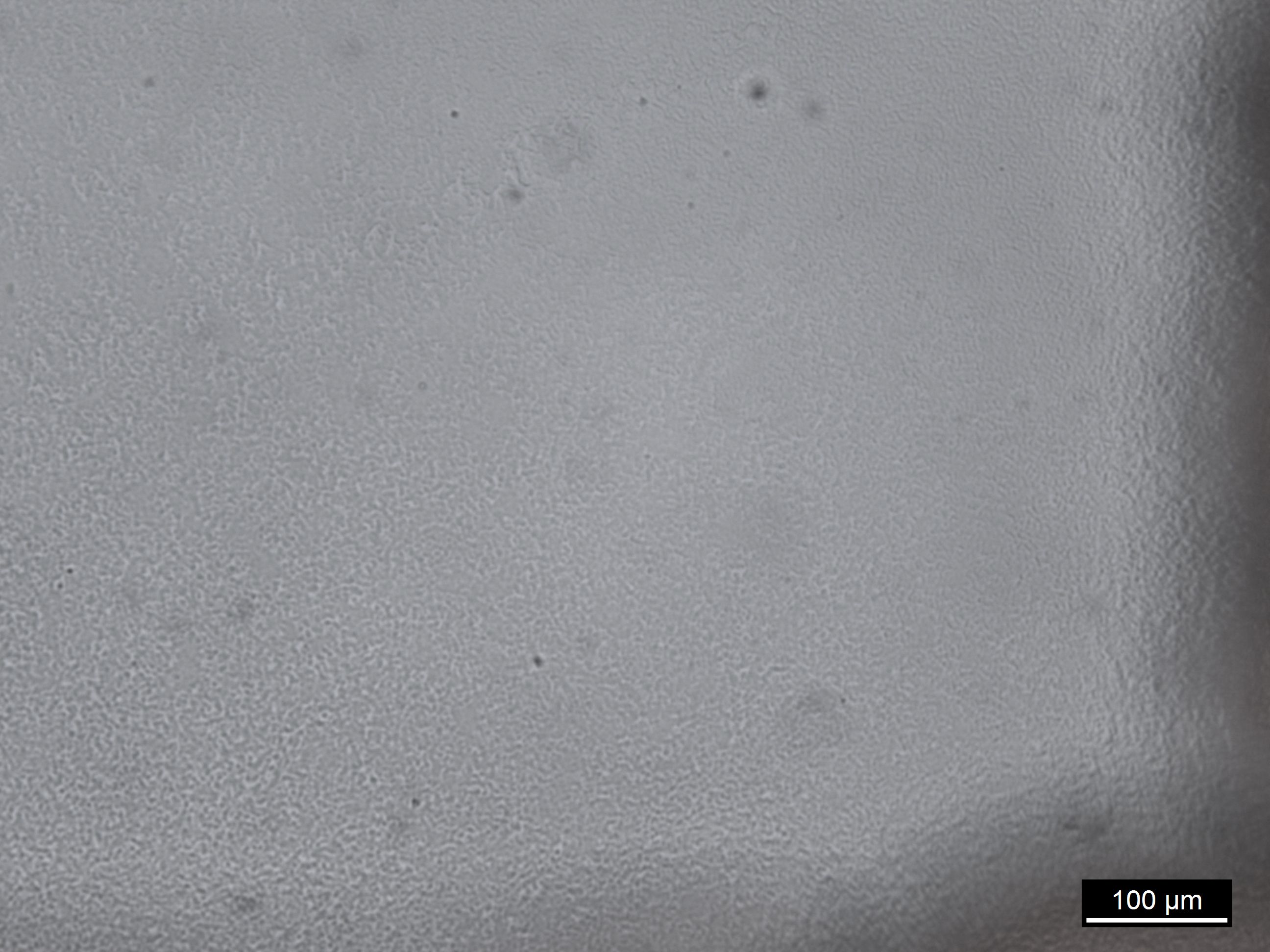

Supplement: Supplementary file 1 [file microorganisms-10-01642-s001.zip › S22_9GU_PHMB_P.jpg]

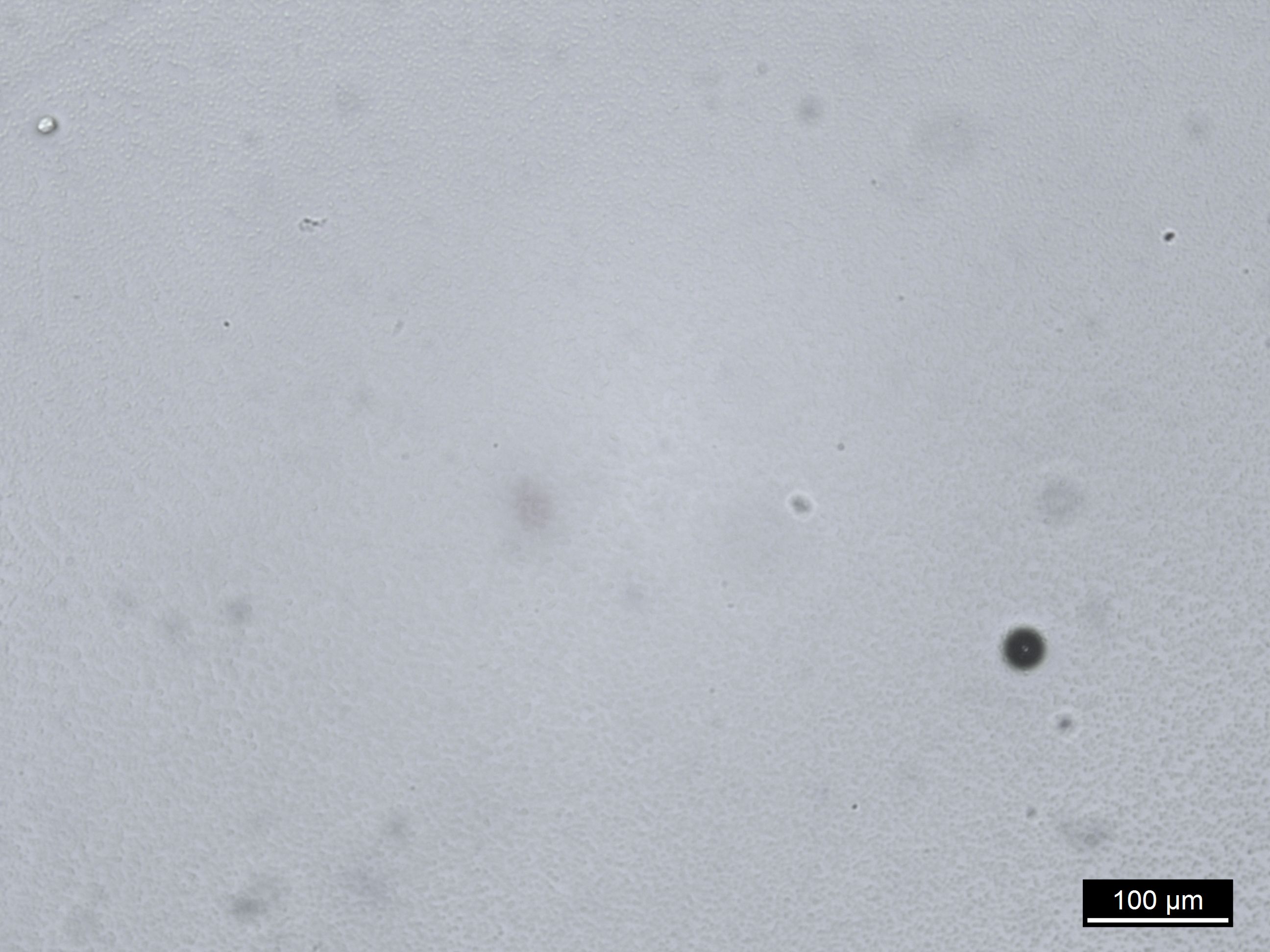

Supplement: Supplementary file 1 [file microorganisms-10-01642-s001.zip › S23_11DS_PHMB_C.jpg]

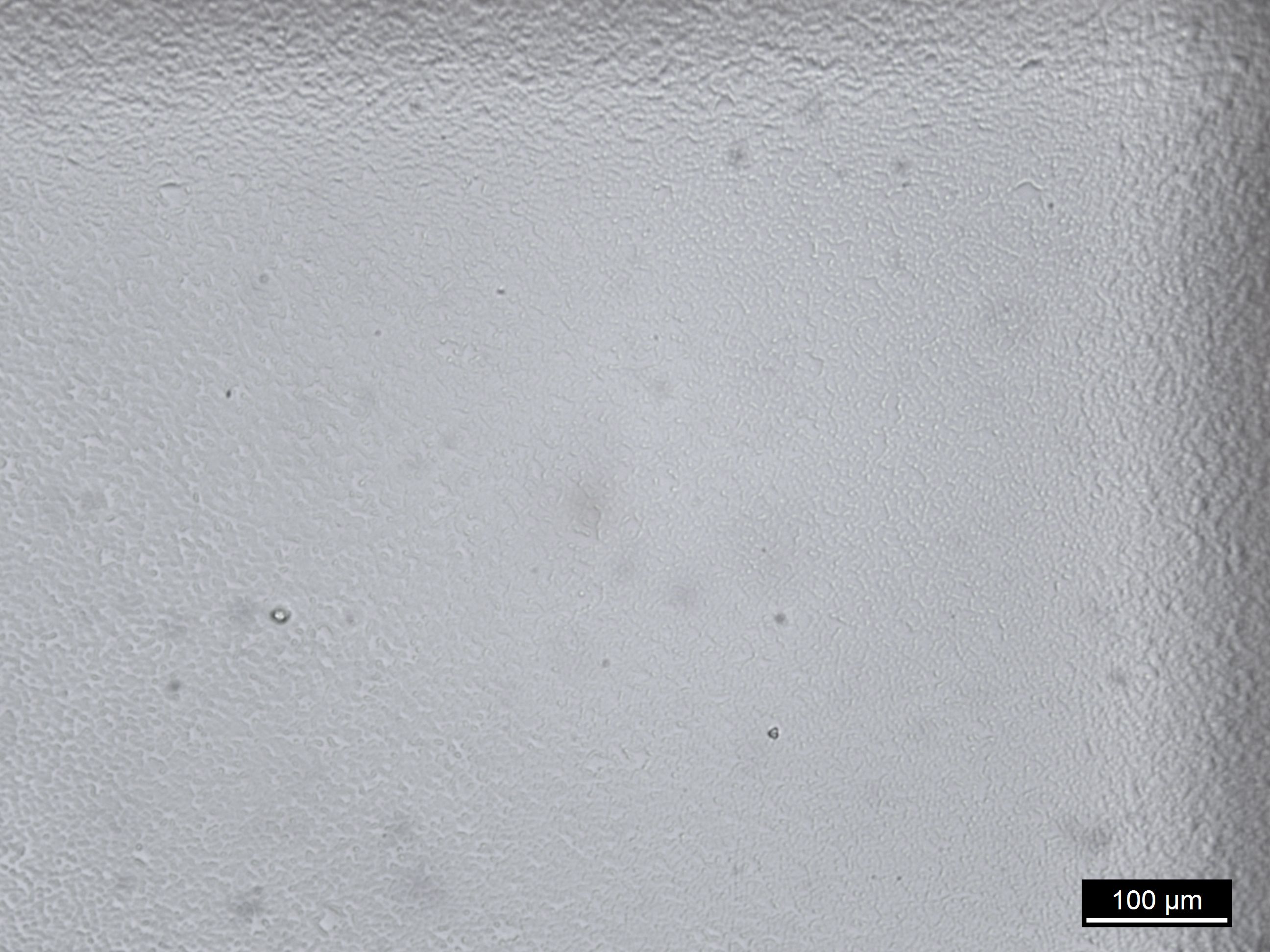

Supplement: Supplementary file 1 [file microorganisms-10-01642-s001.zip › S24_11DS_PHMB_P.jpg]

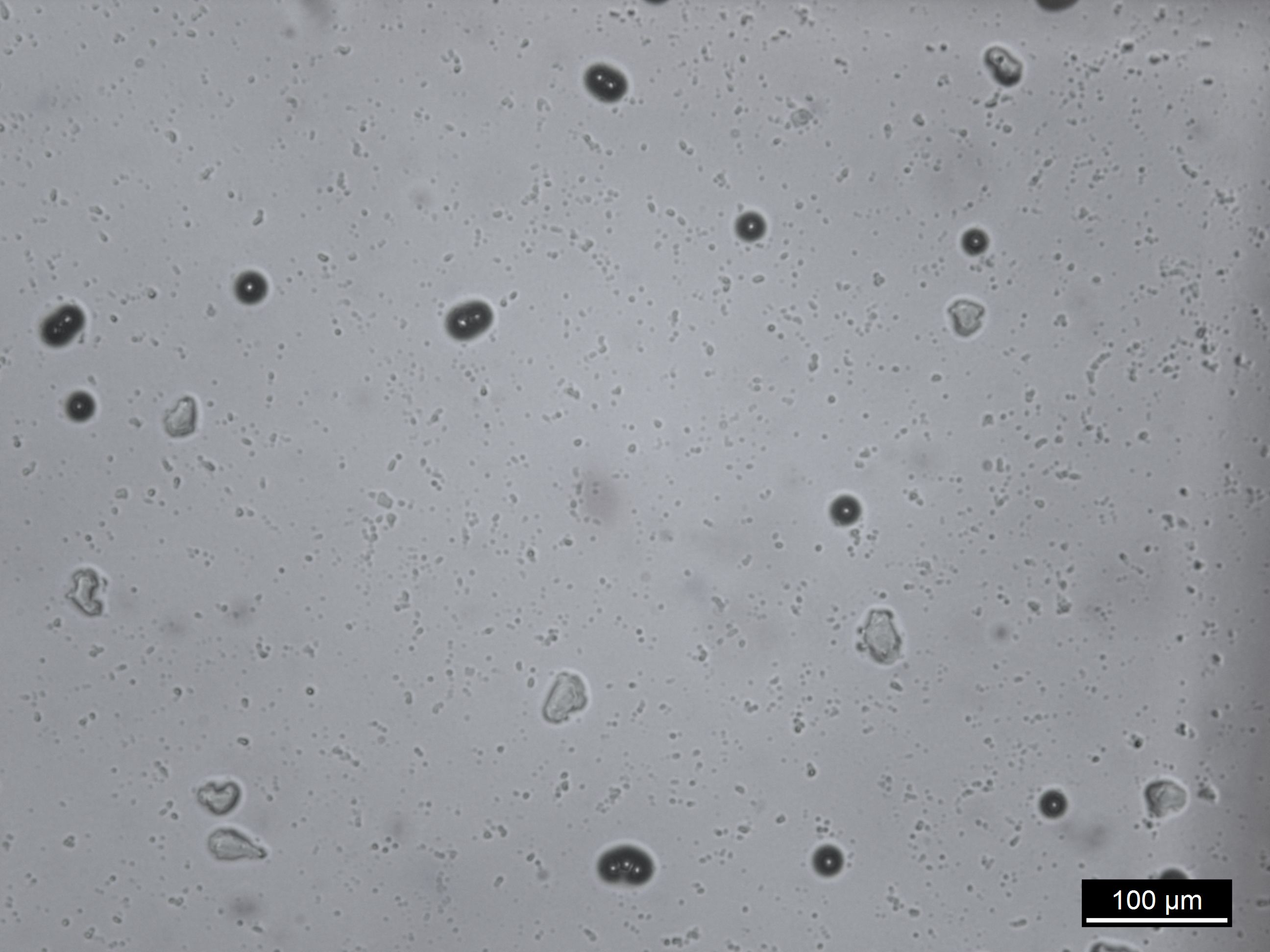

Supplement: Supplementary file 1 [file microorganisms-10-01642-s001.zip › S25_IBU_CH_C.jpg]

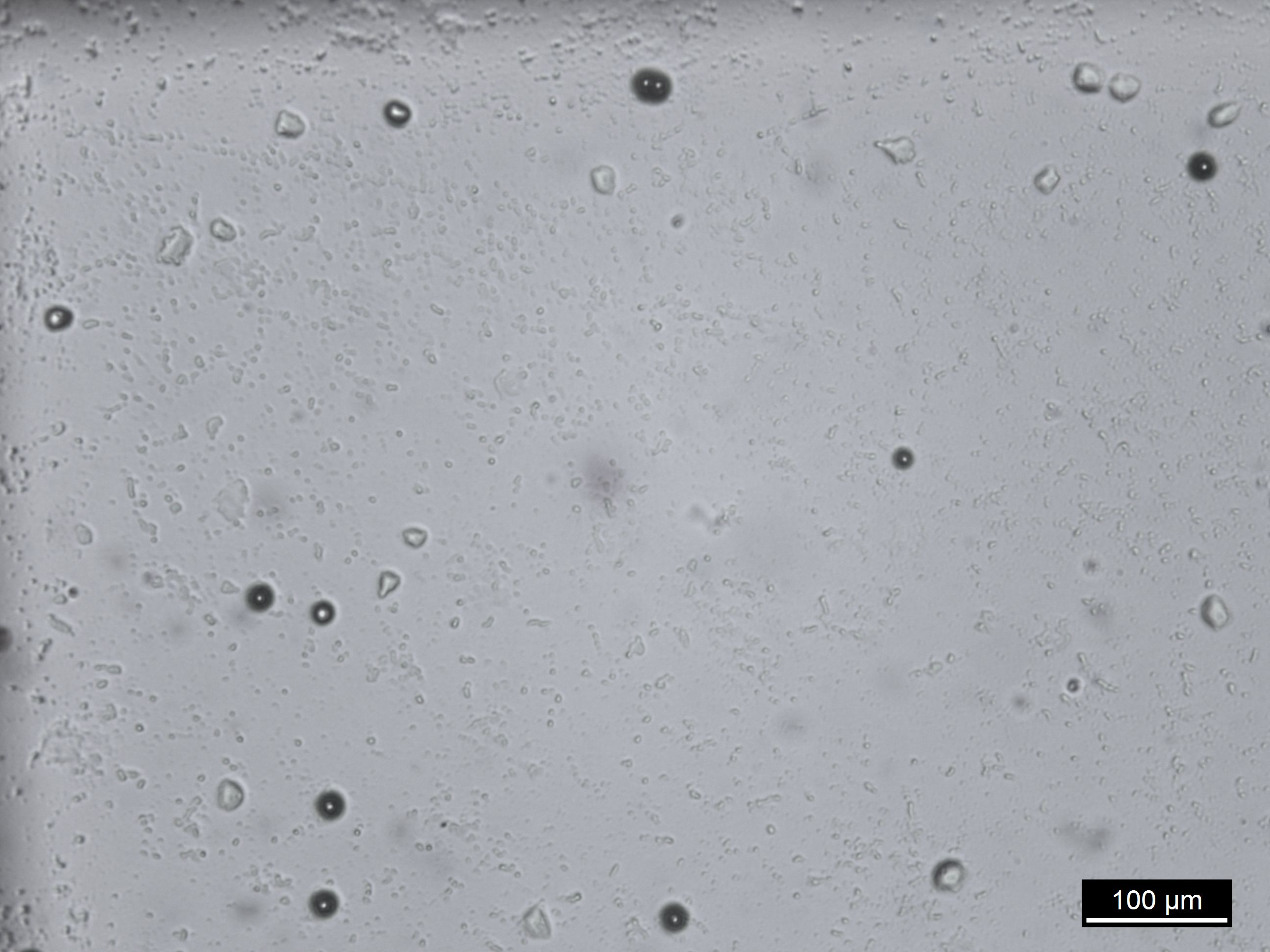

Supplement: Supplementary file 1 [file microorganisms-10-01642-s001.zip › S26_IBU_CH_P.jpg]

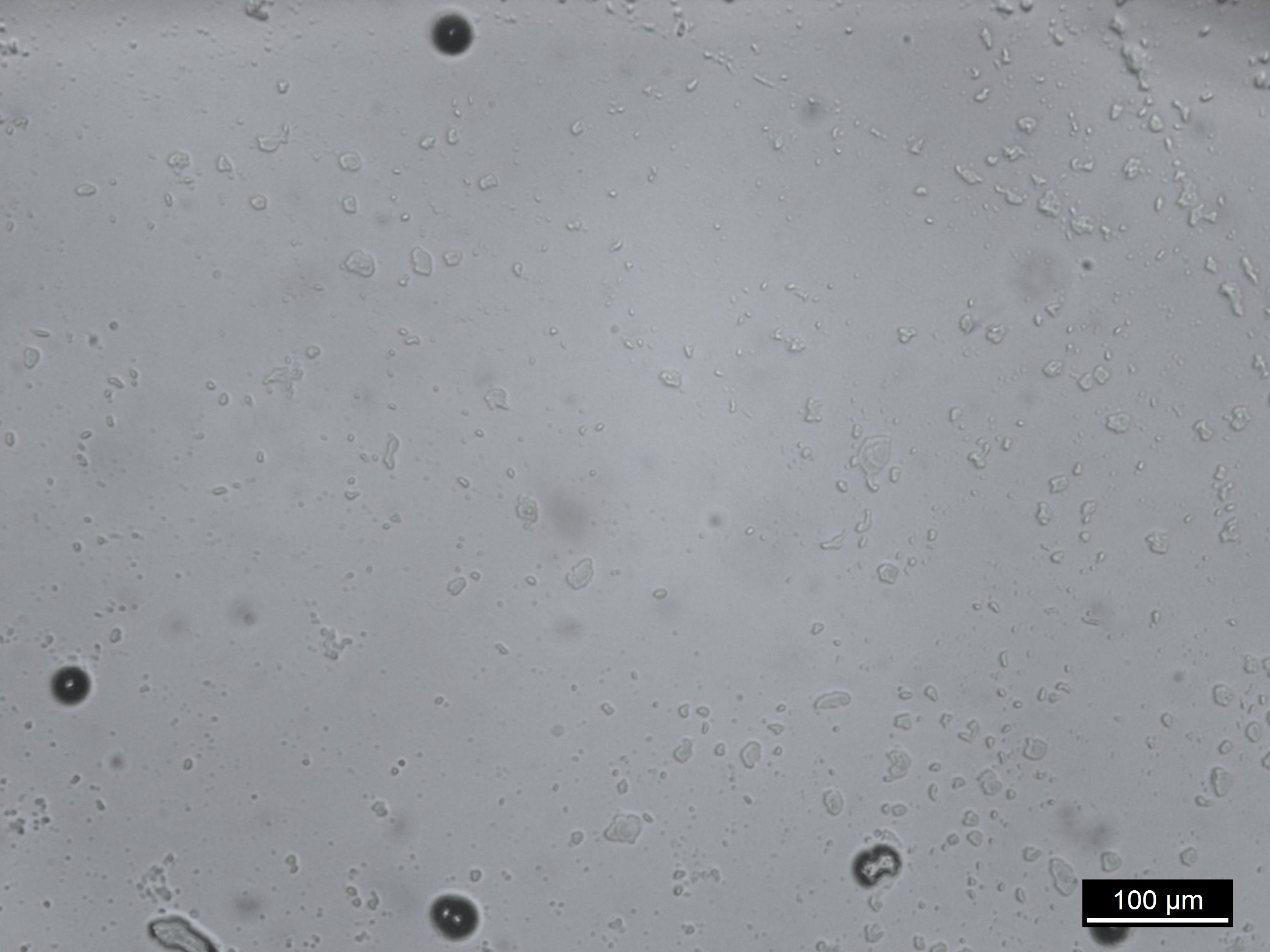

Supplement: Supplementary file 1 [file microorganisms-10-01642-s001.zip › S27_3ST_CH_C.jpg]

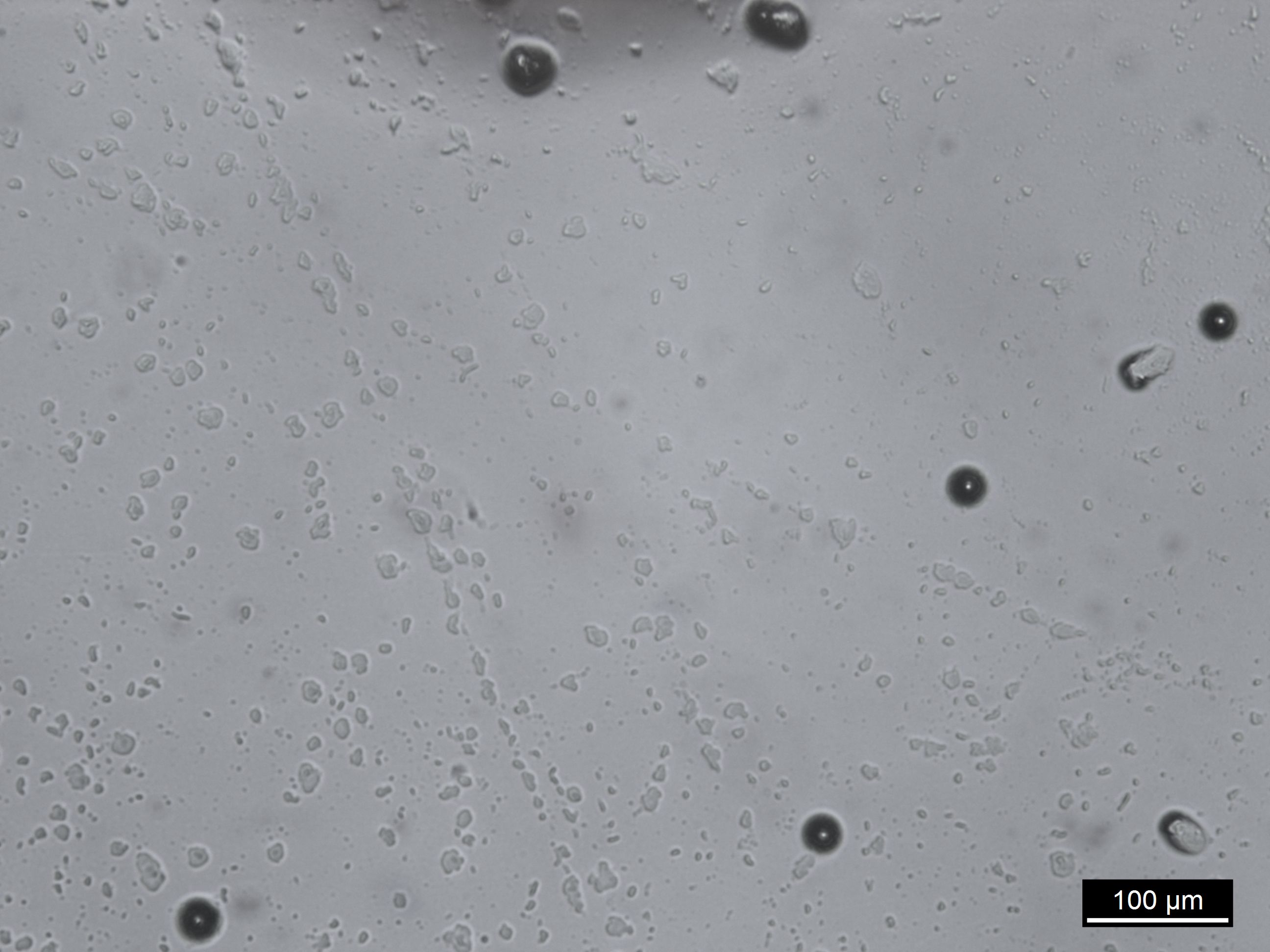

Supplement: Supplementary file 1 [file microorganisms-10-01642-s001.zip › S28_3ST_CH_P.jpg]

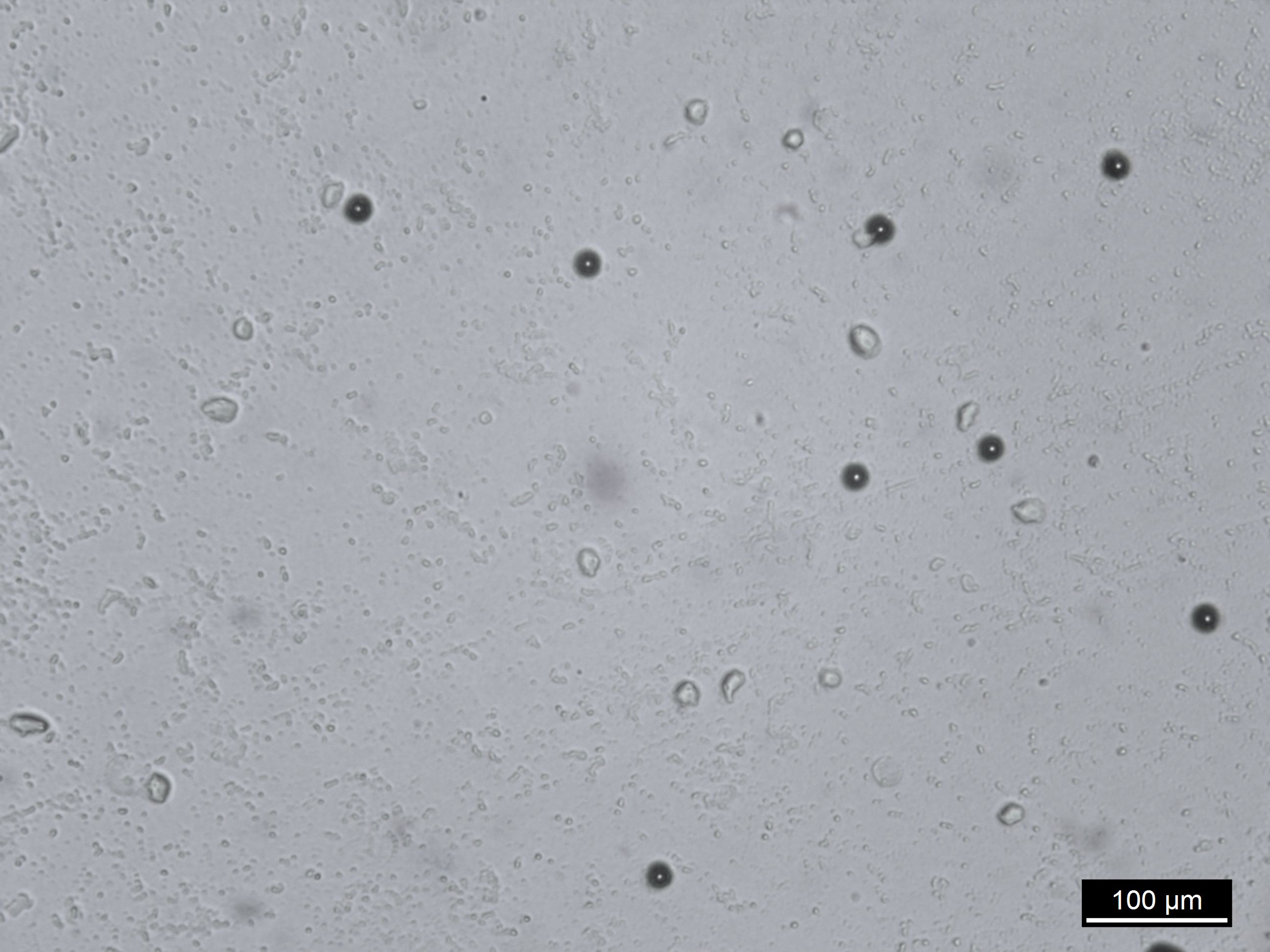

Supplement: Supplementary file 1 [file microorganisms-10-01642-s001.zip › S29_9GU_CH_C.jpg]

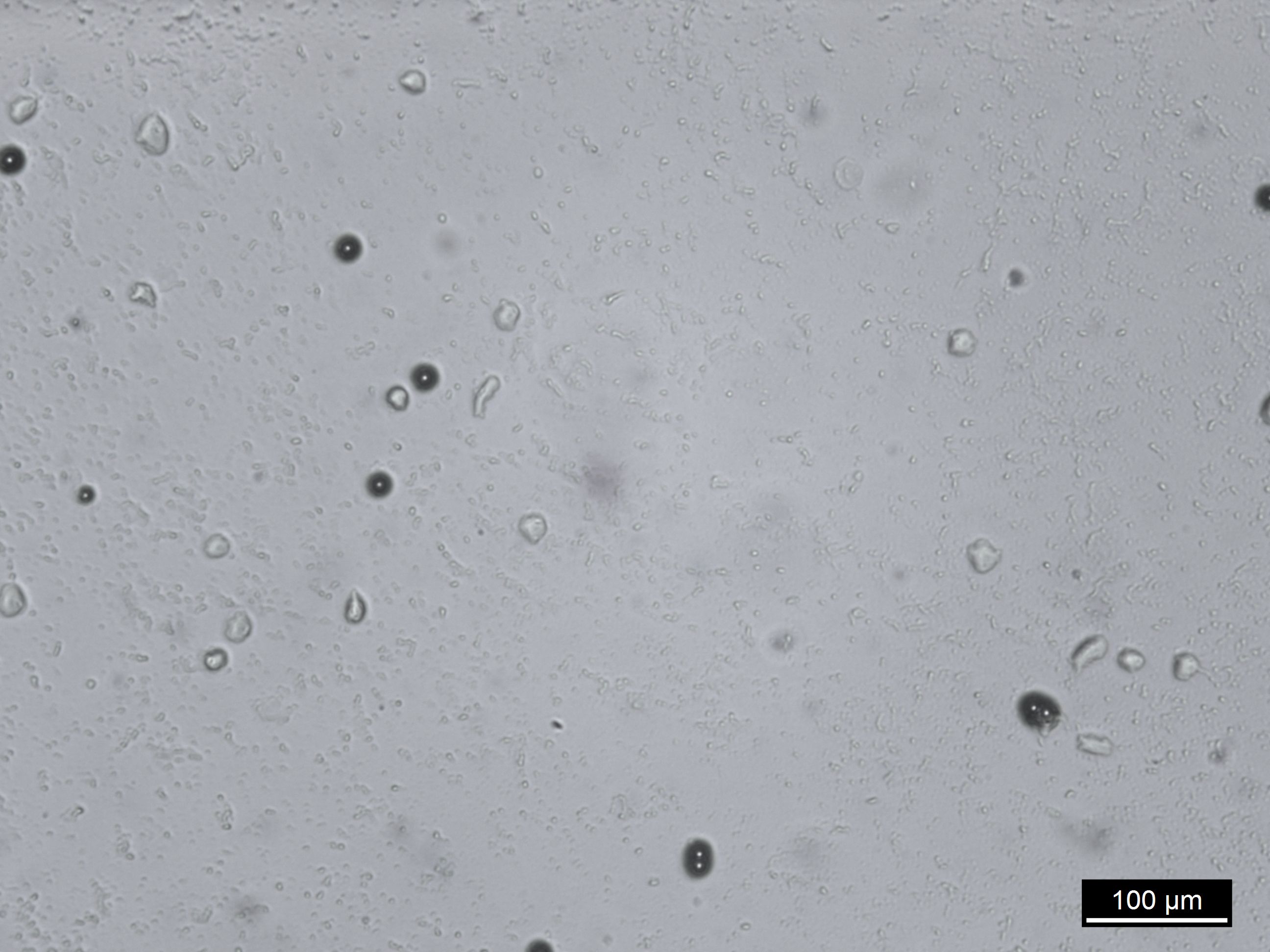

Supplement: Supplementary file 1 [file microorganisms-10-01642-s001.zip › S2_IBU_Control_P.jpg]

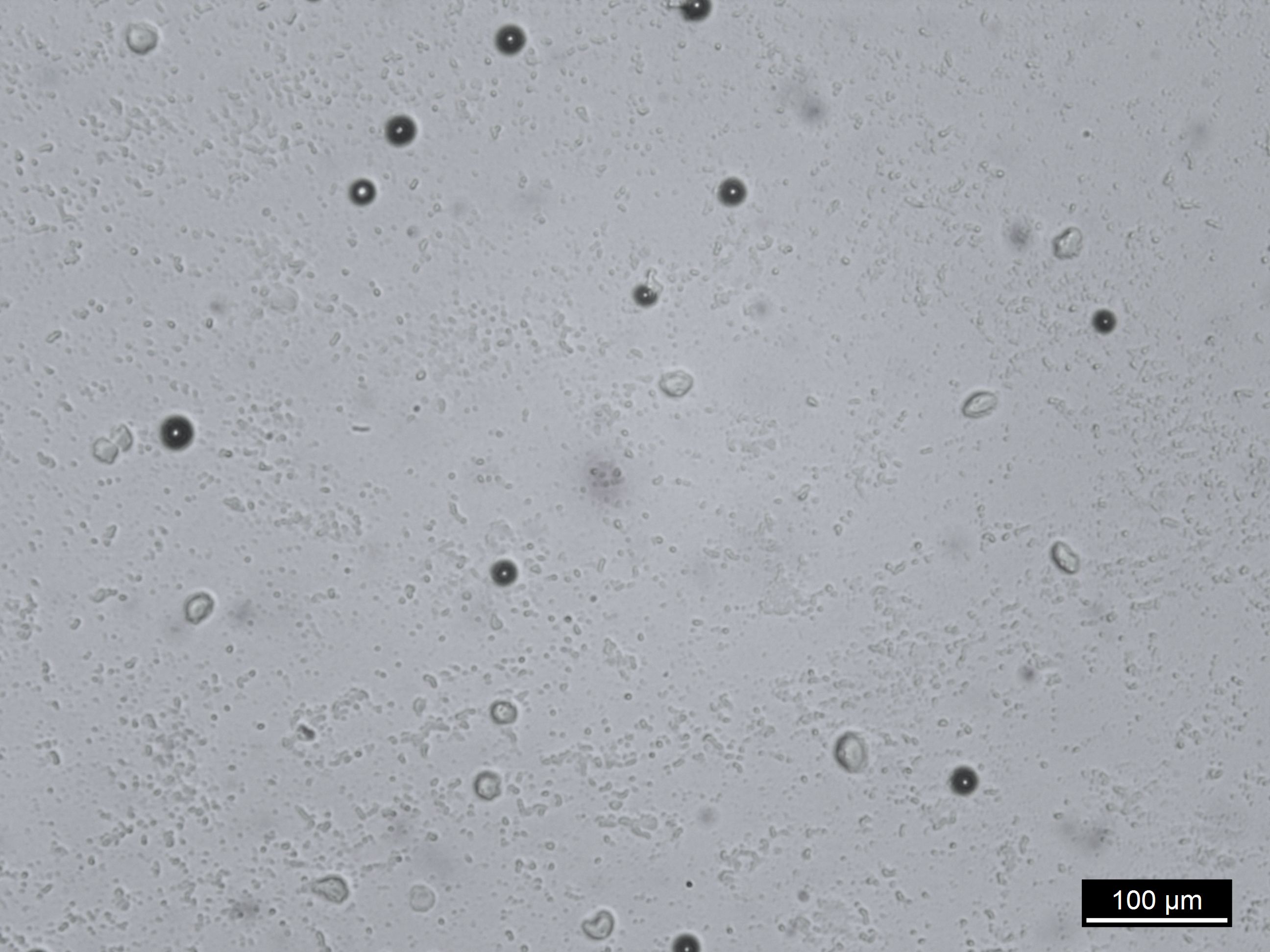

Supplement: Supplementary file 1 [file microorganisms-10-01642-s001.zip › S30_9GU_CH_P.jpg]

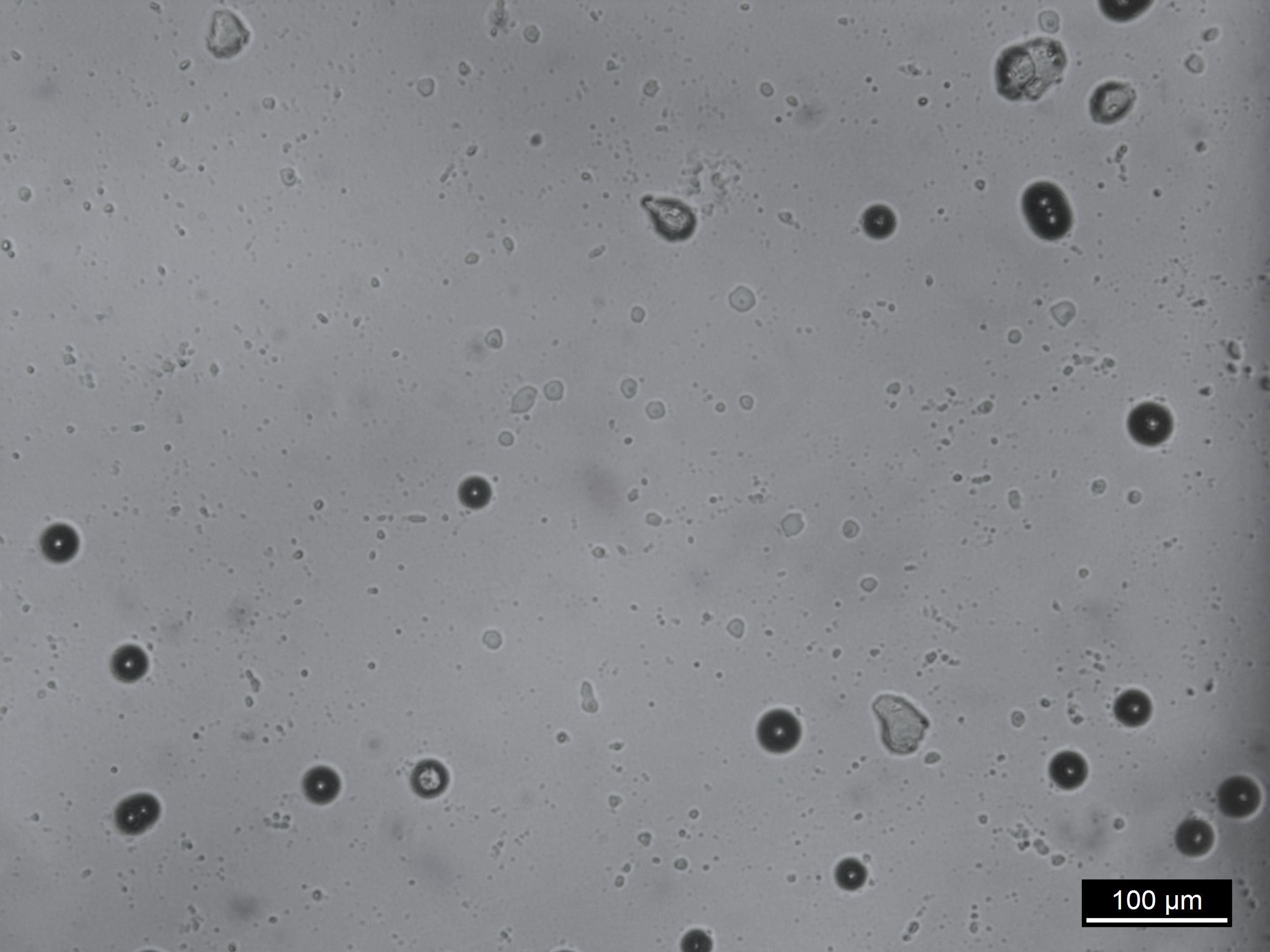

Supplement: Supplementary file 1 [file microorganisms-10-01642-s001.zip › S31_11DS_CH_C.jpg]

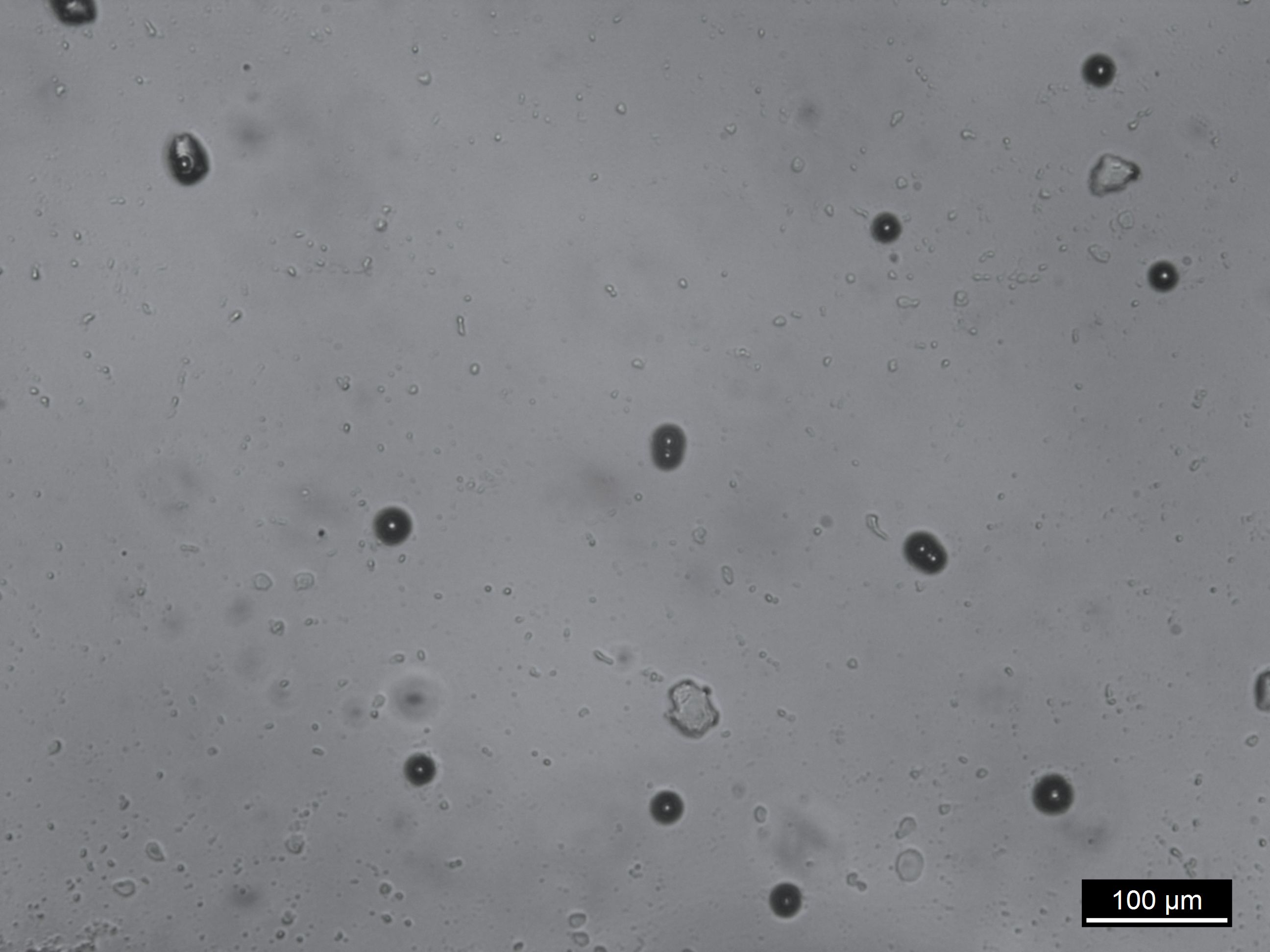

Supplement: Supplementary file 1 [file microorganisms-10-01642-s001.zip › S32_11DS_CH_P.jpg]

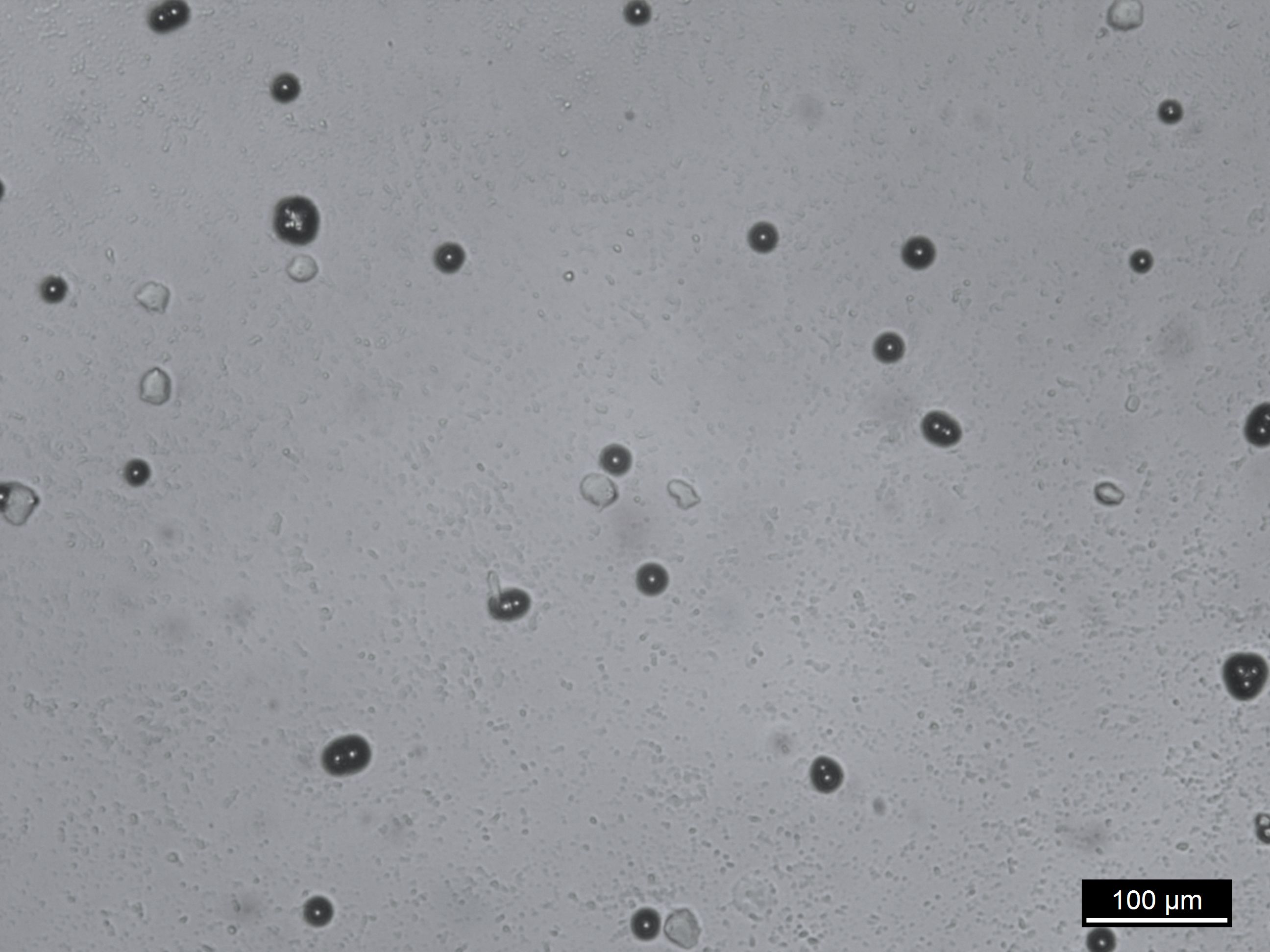

Supplement: Supplementary file 1 [file microorganisms-10-01642-s001.zip › S33_IBU_DD_C.jpg]

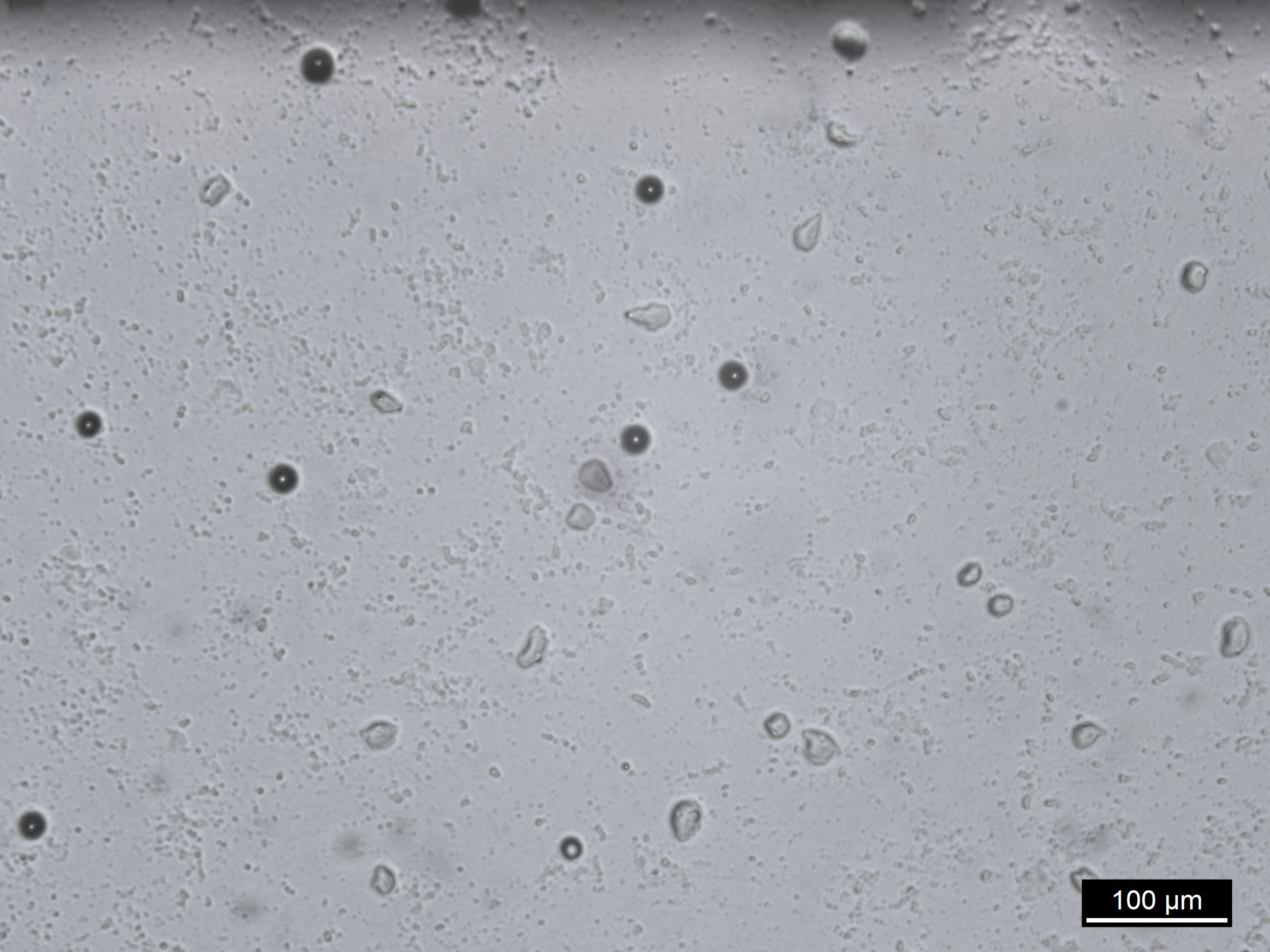

Supplement: Supplementary file 1 [file microorganisms-10-01642-s001.zip › S34_IBU_DD_P.jpg]

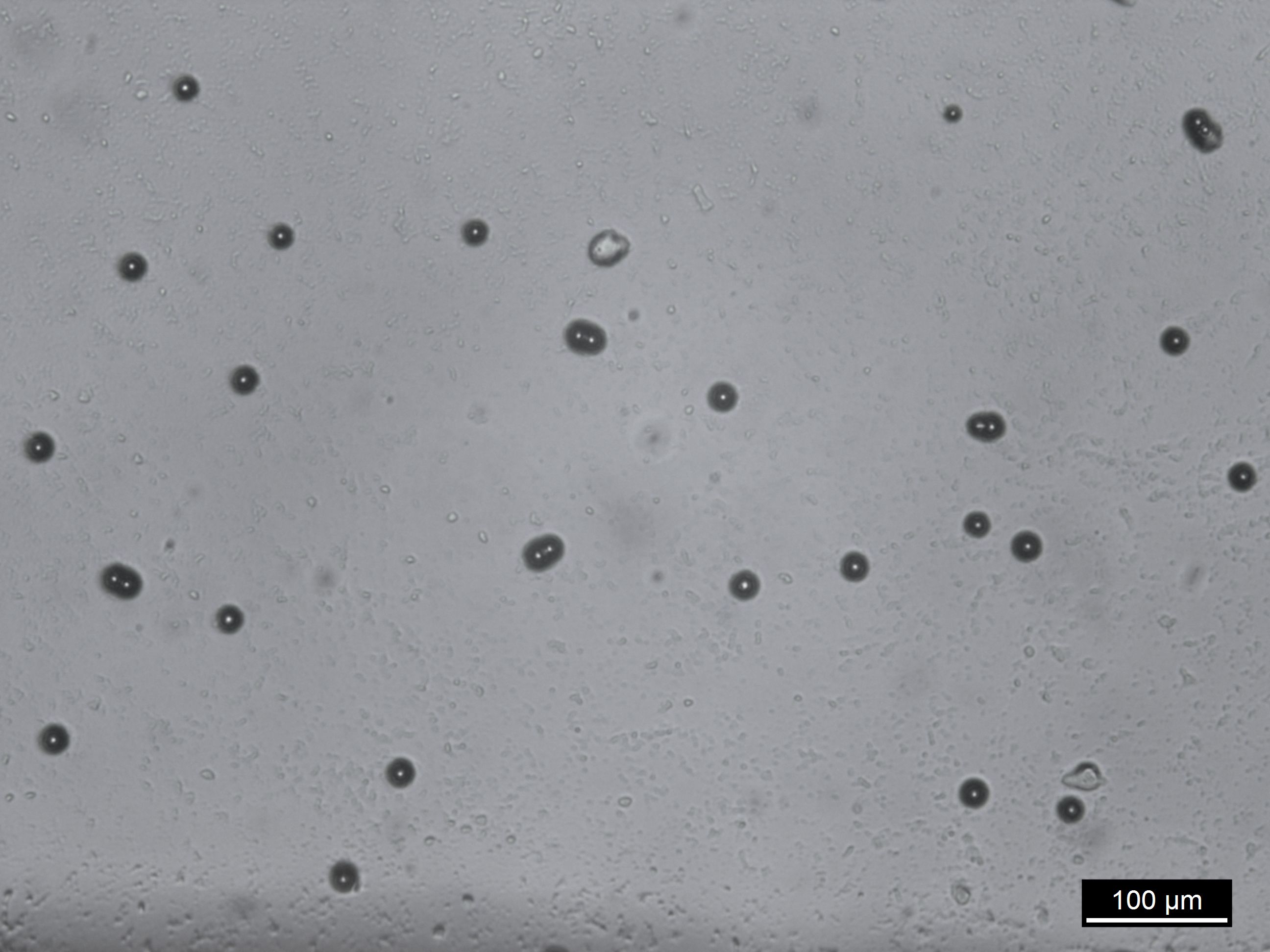

Supplement: Supplementary file 1 [file microorganisms-10-01642-s001.zip › S35_3ST_DD_C.jpg]

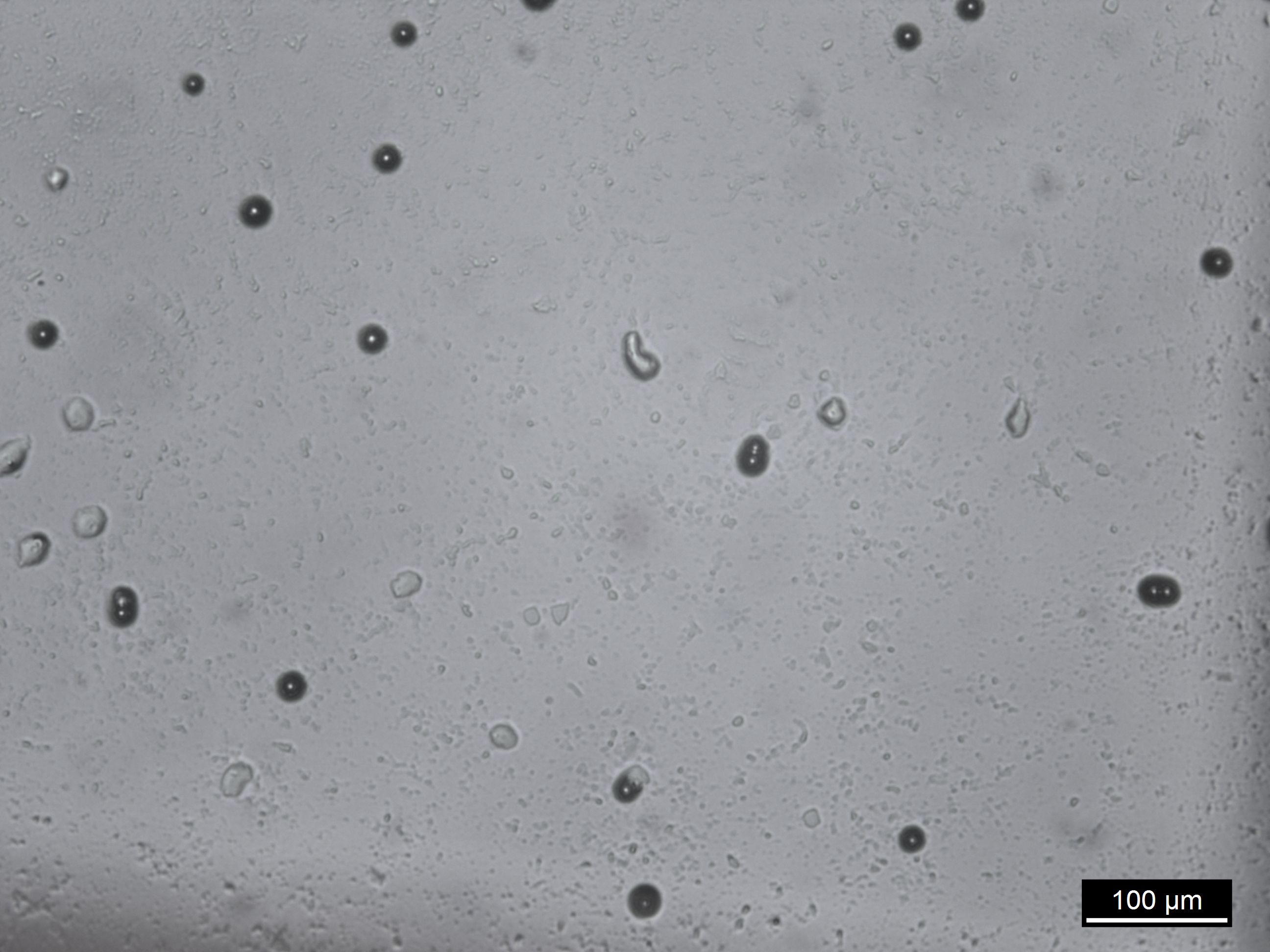

Supplement: Supplementary file 1 [file microorganisms-10-01642-s001.zip › S36_3ST_DD_P.jpg]

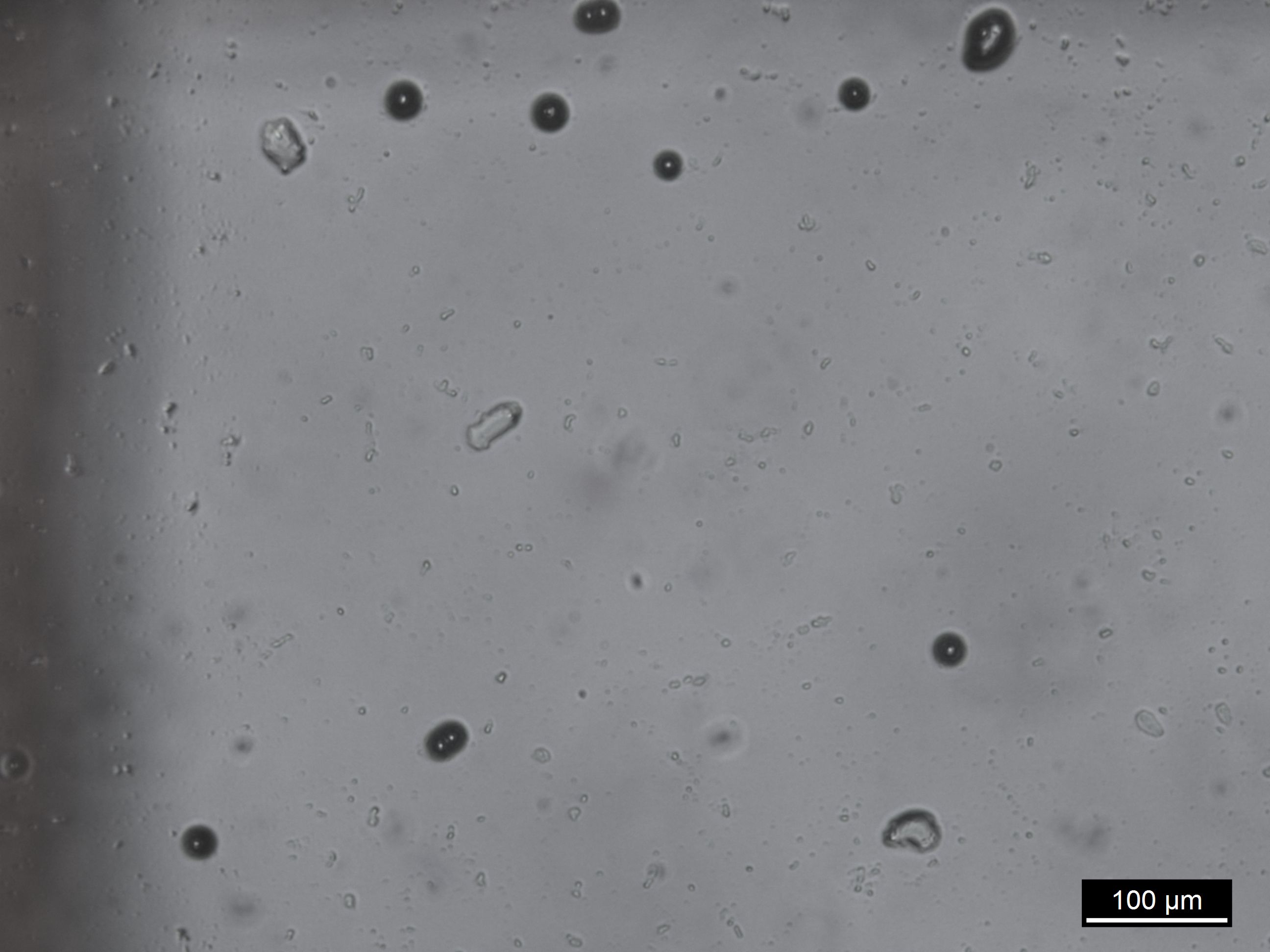

Supplement: Supplementary file 1 [file microorganisms-10-01642-s001.zip › S37_9GU_DD_C.jpg]

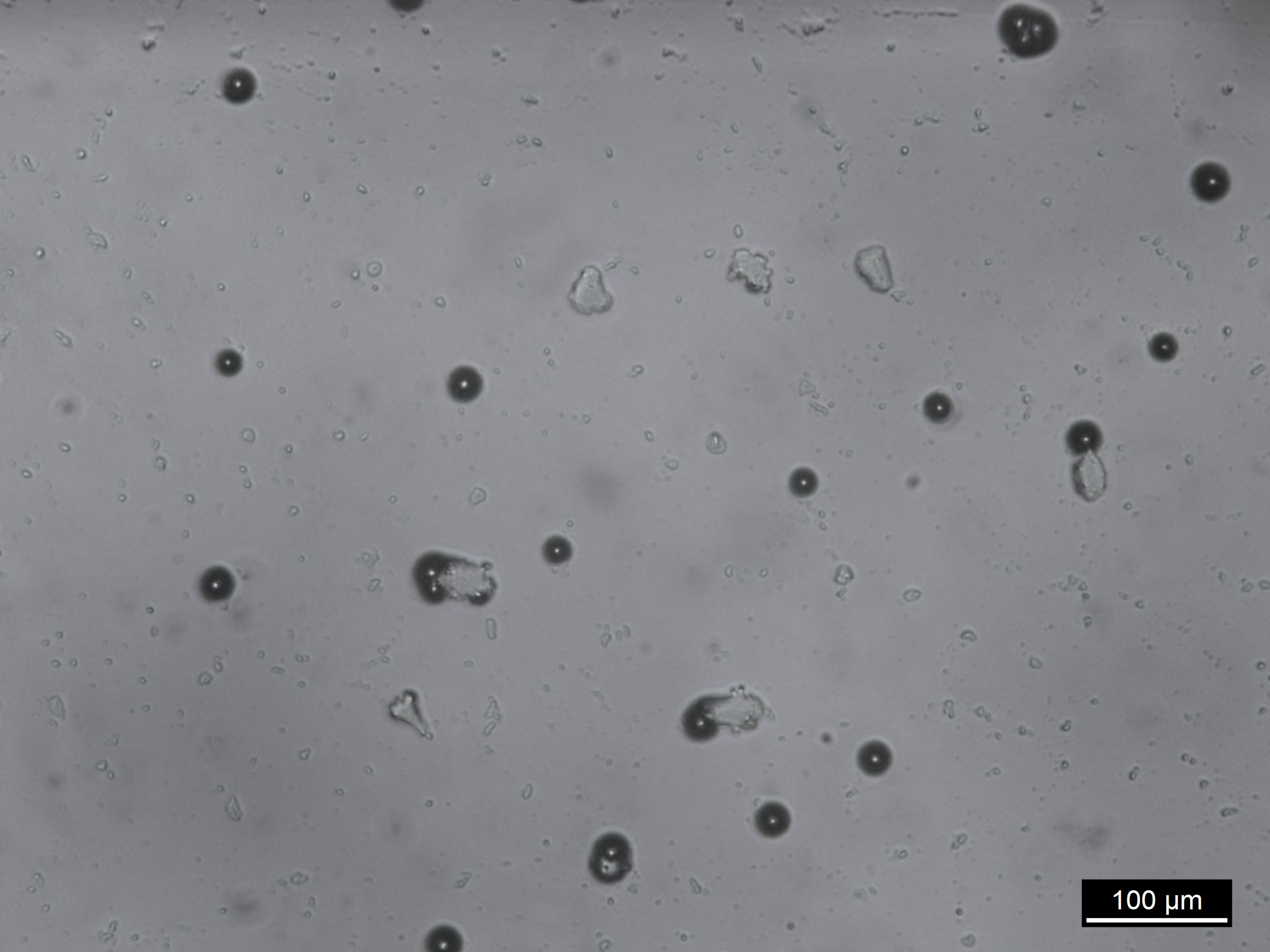

Supplement: Supplementary file 1 [file microorganisms-10-01642-s001.zip › S38_9GU_DD_P.jpg]

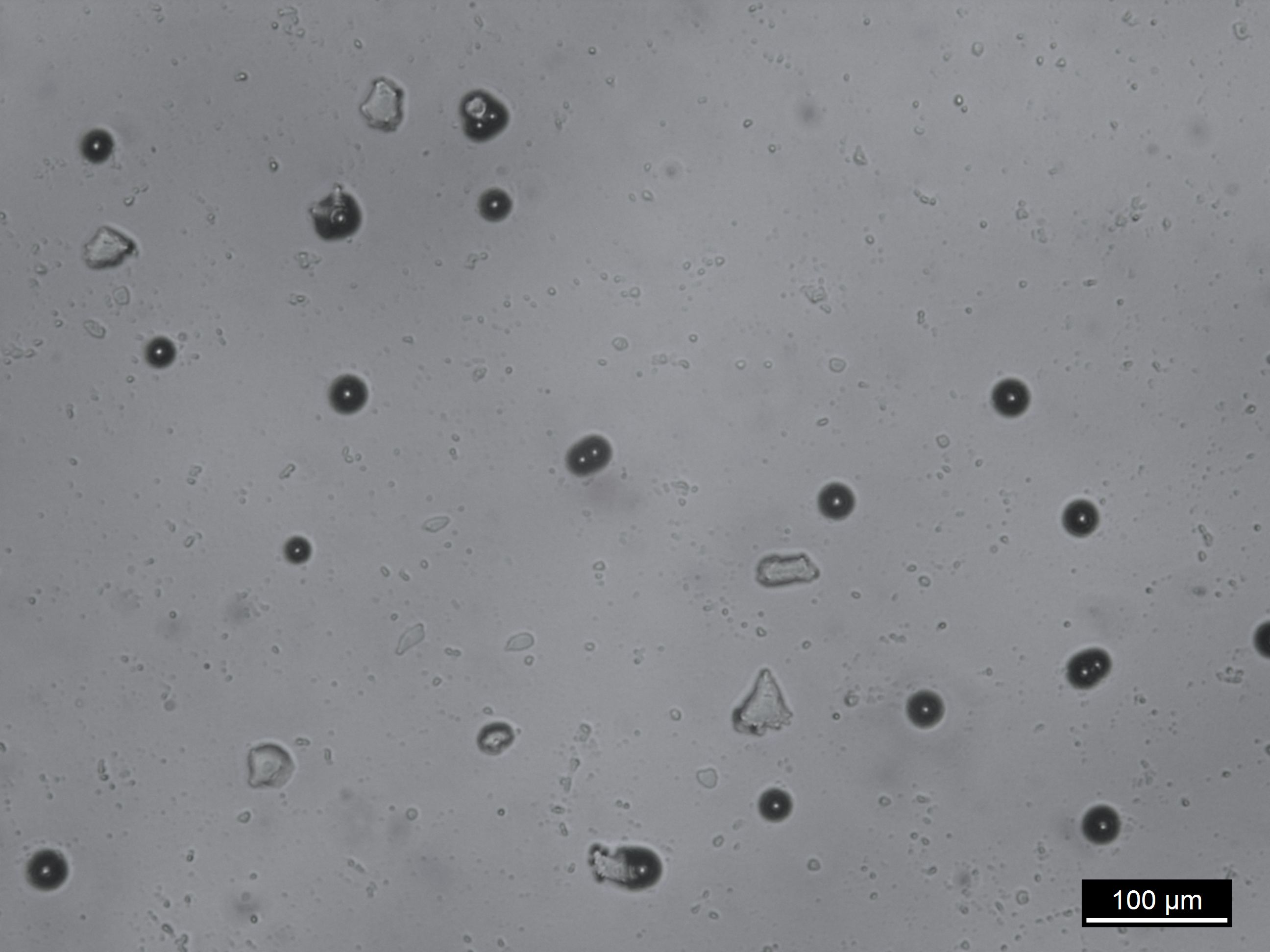

Supplement: Supplementary file 1 [file microorganisms-10-01642-s001.zip › S39_11DS_DD_C.jpg]

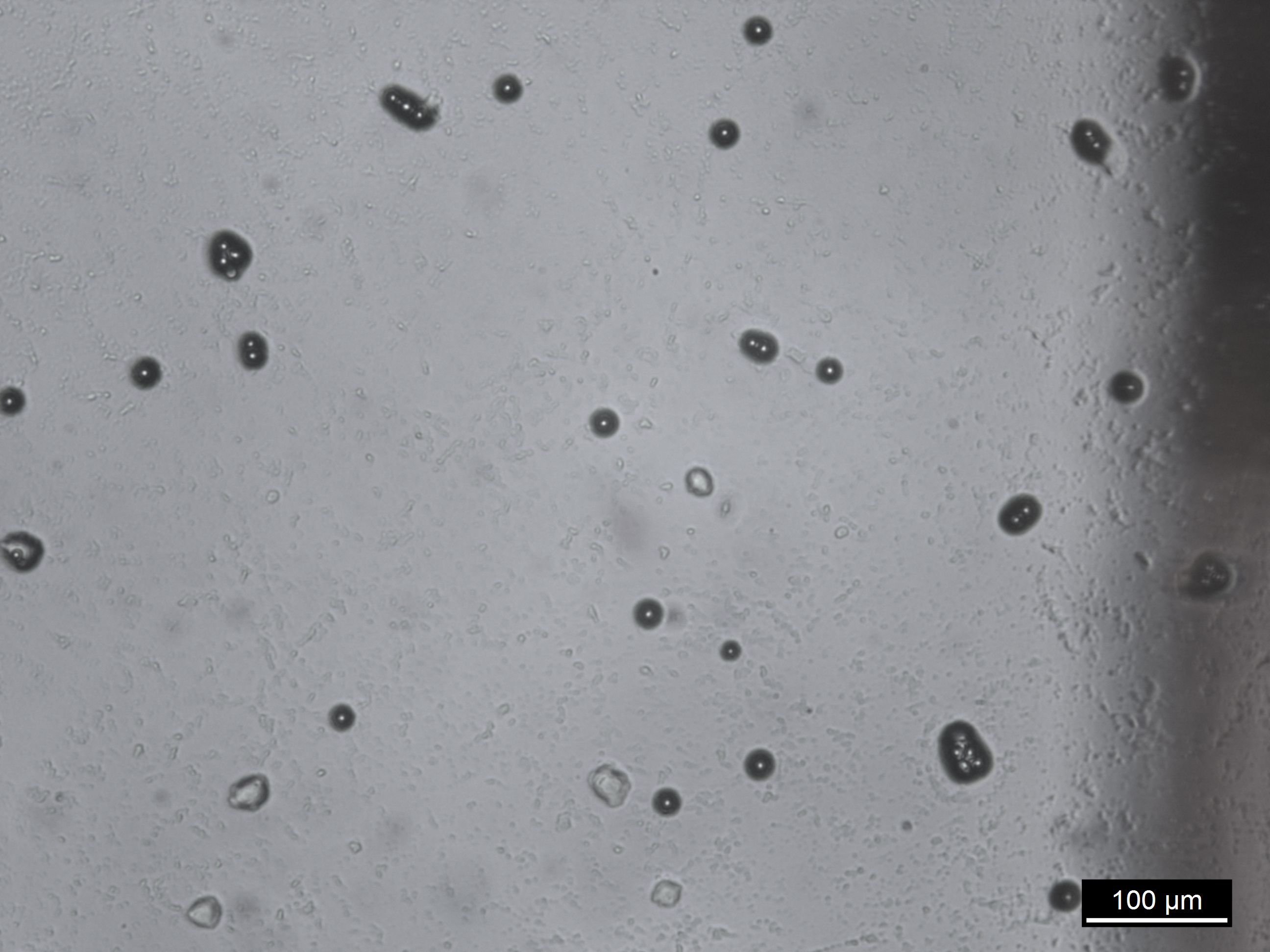

Supplement: Supplementary file 1 [file microorganisms-10-01642-s001.zip › S3_3ST_Control_C.jpg]

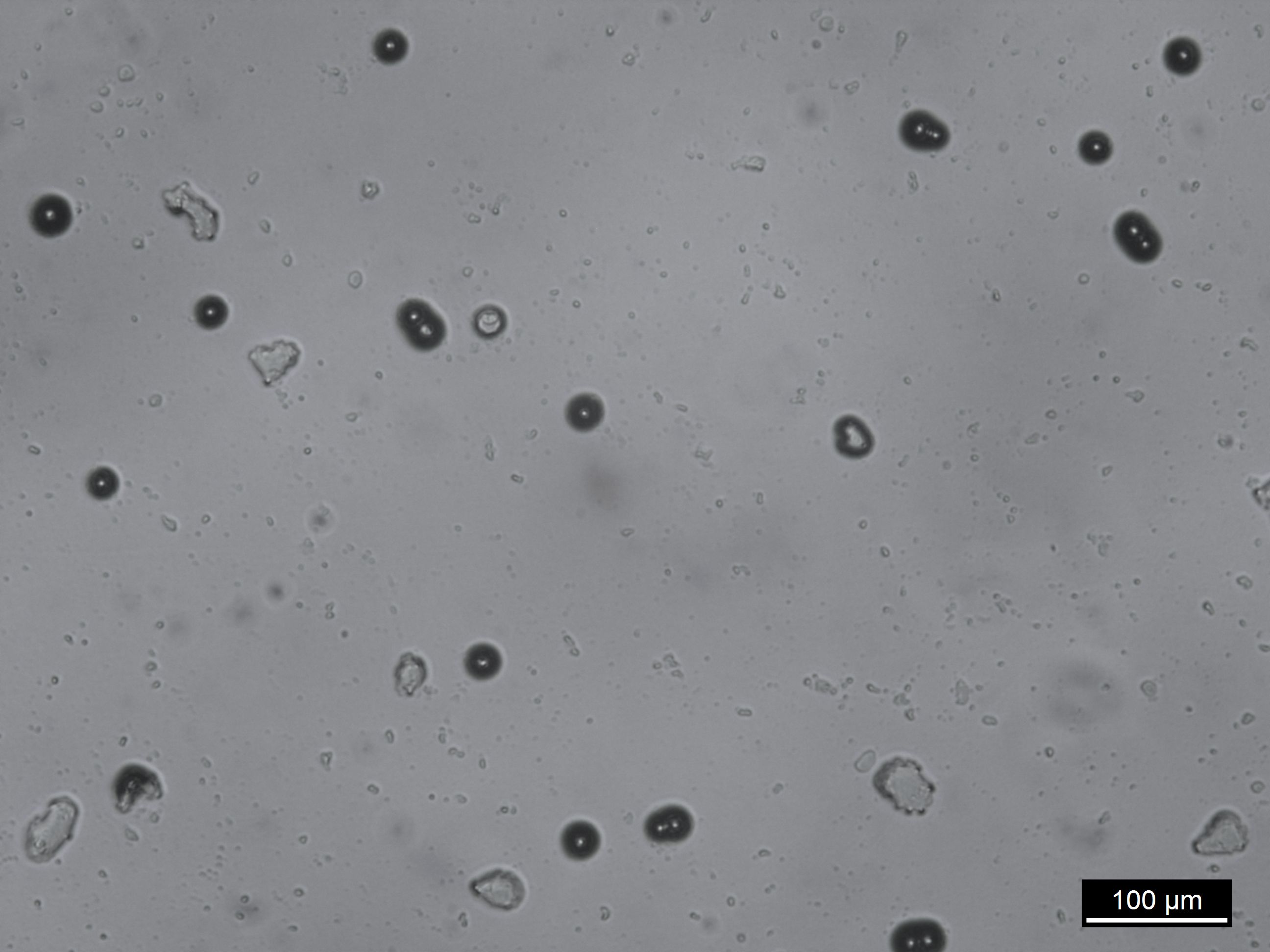

Supplement: Supplementary file 1 [file microorganisms-10-01642-s001.zip › S40_11DS_DD_P.jpg]

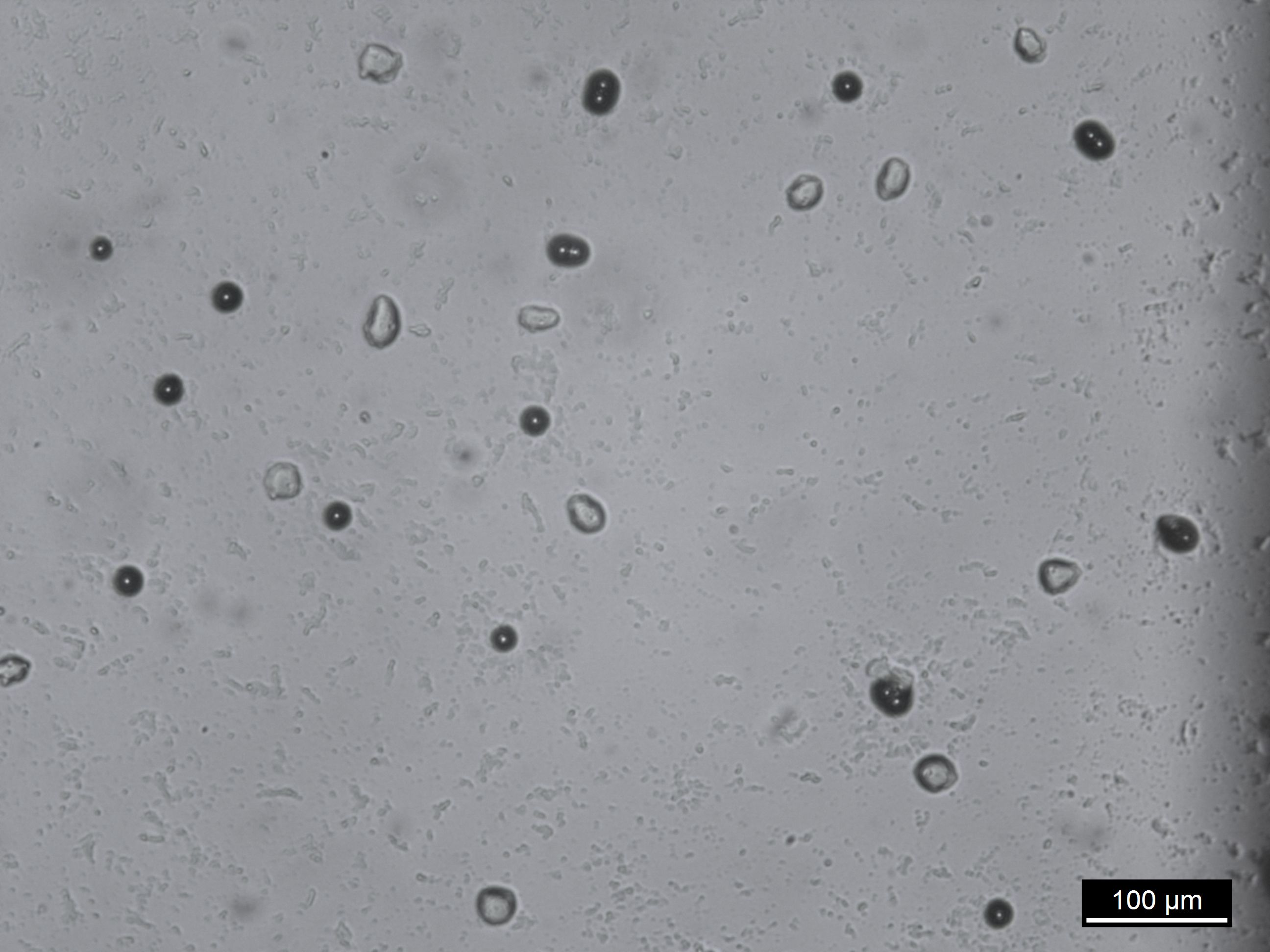

Supplement: Supplementary file 1 [file microorganisms-10-01642-s001.zip › S41_IBU_HD_C.jpg]

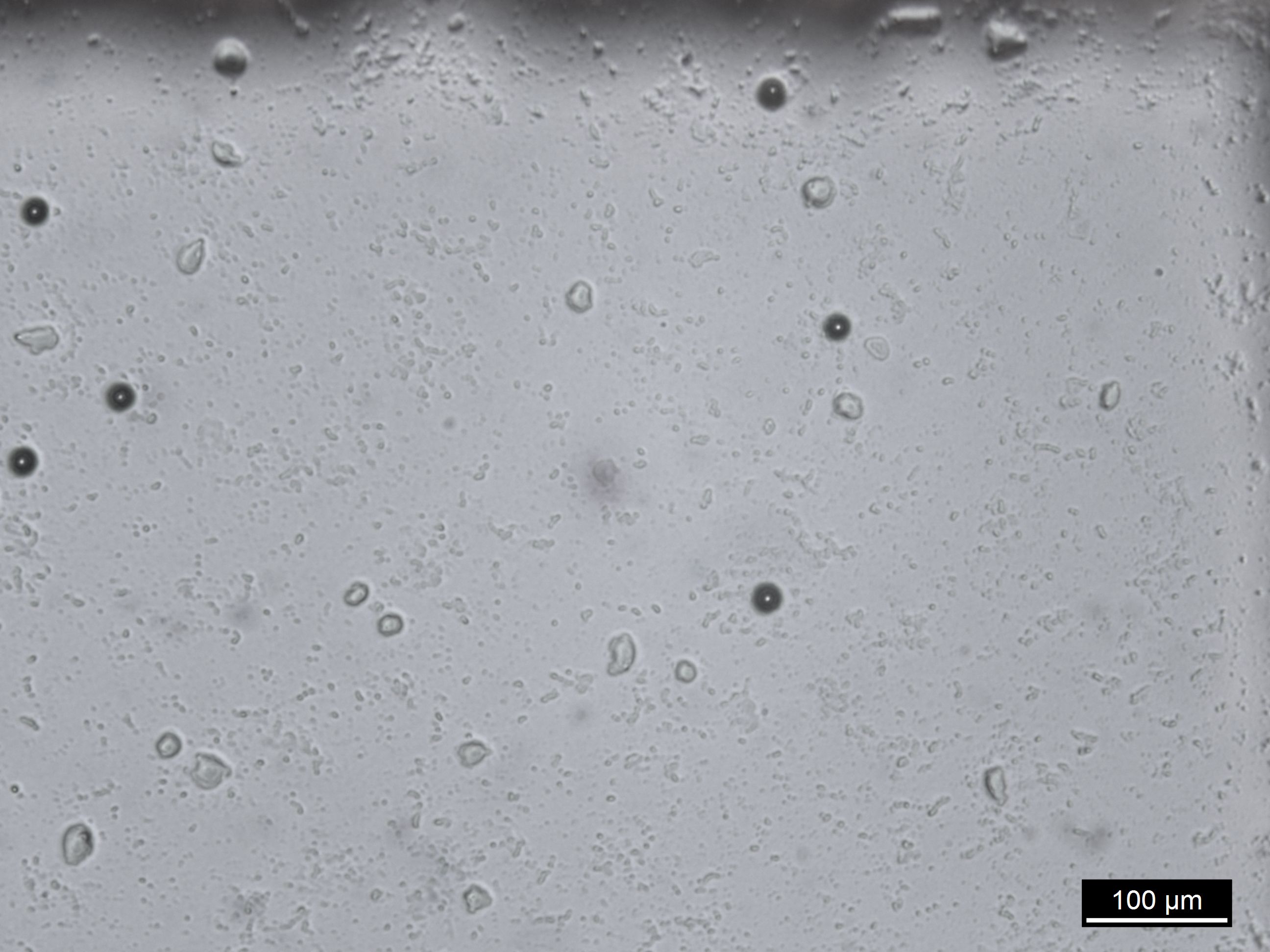

Supplement: Supplementary file 1 [file microorganisms-10-01642-s001.zip › S42_IBU_HD_P.jpg]

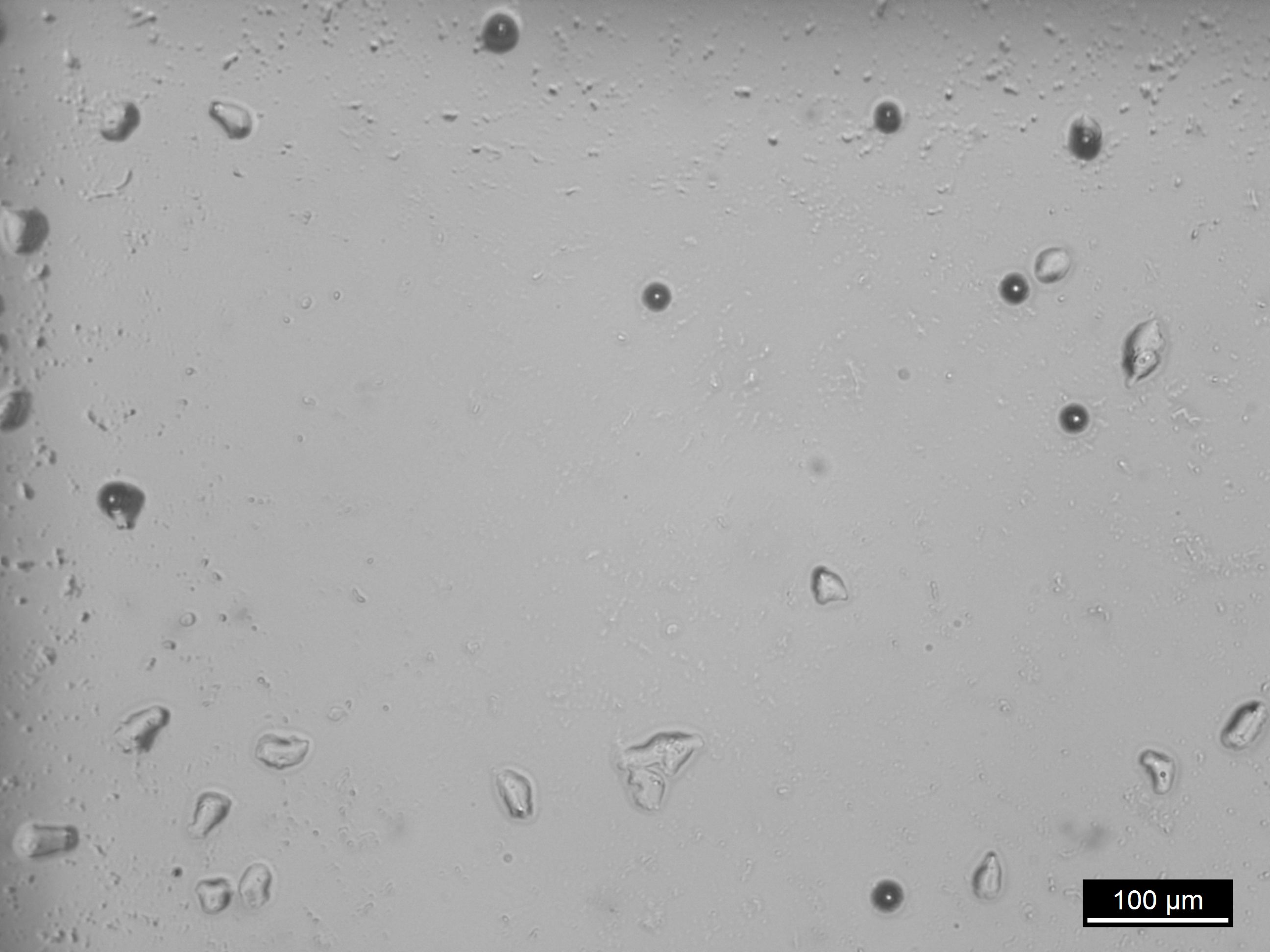

Supplement: Supplementary file 1 [file microorganisms-10-01642-s001.zip › S43_3ST_HD_C.jpg]

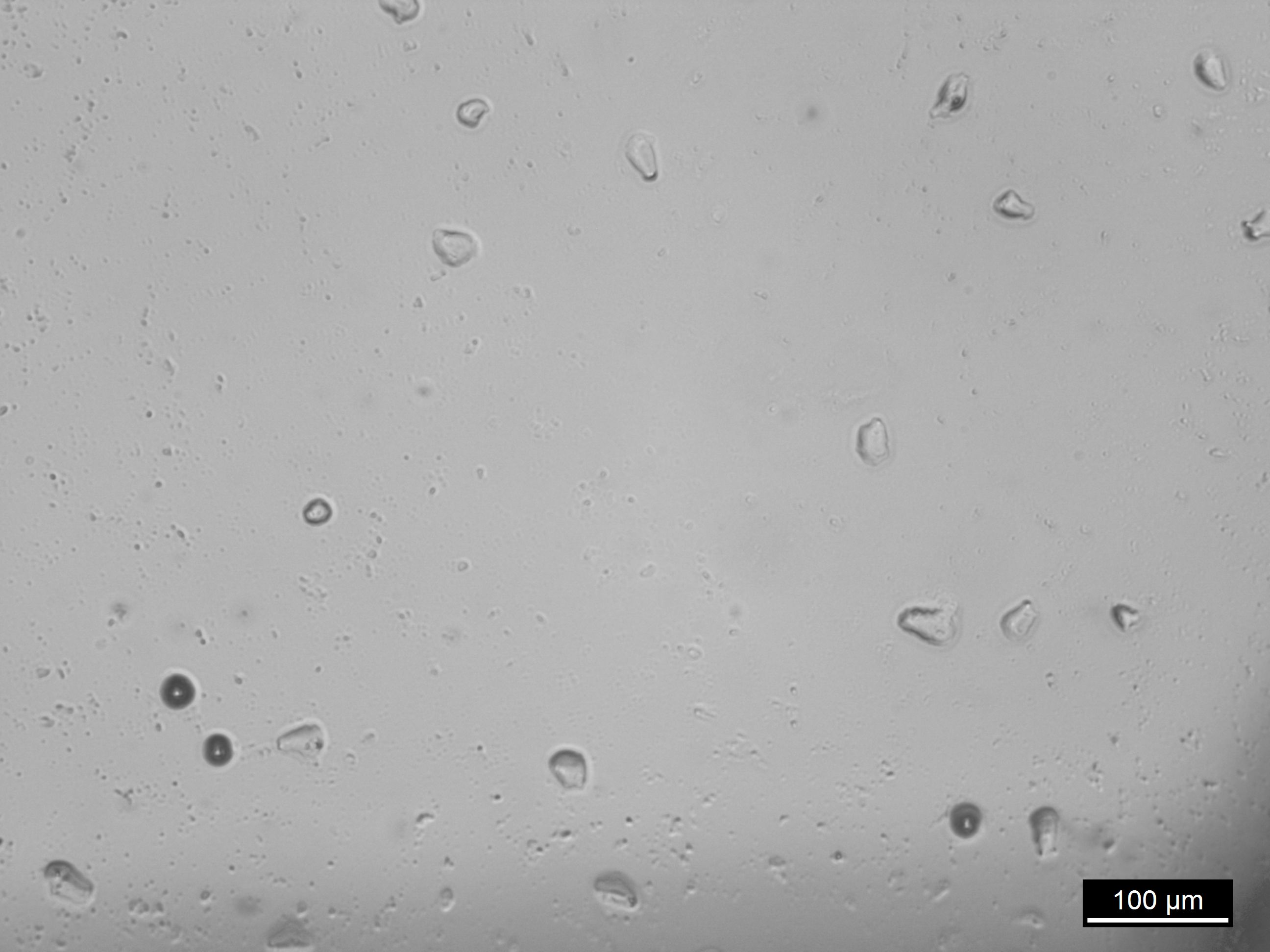

Supplement: Supplementary file 1 [file microorganisms-10-01642-s001.zip › S44_3ST_HD_P.jpg]

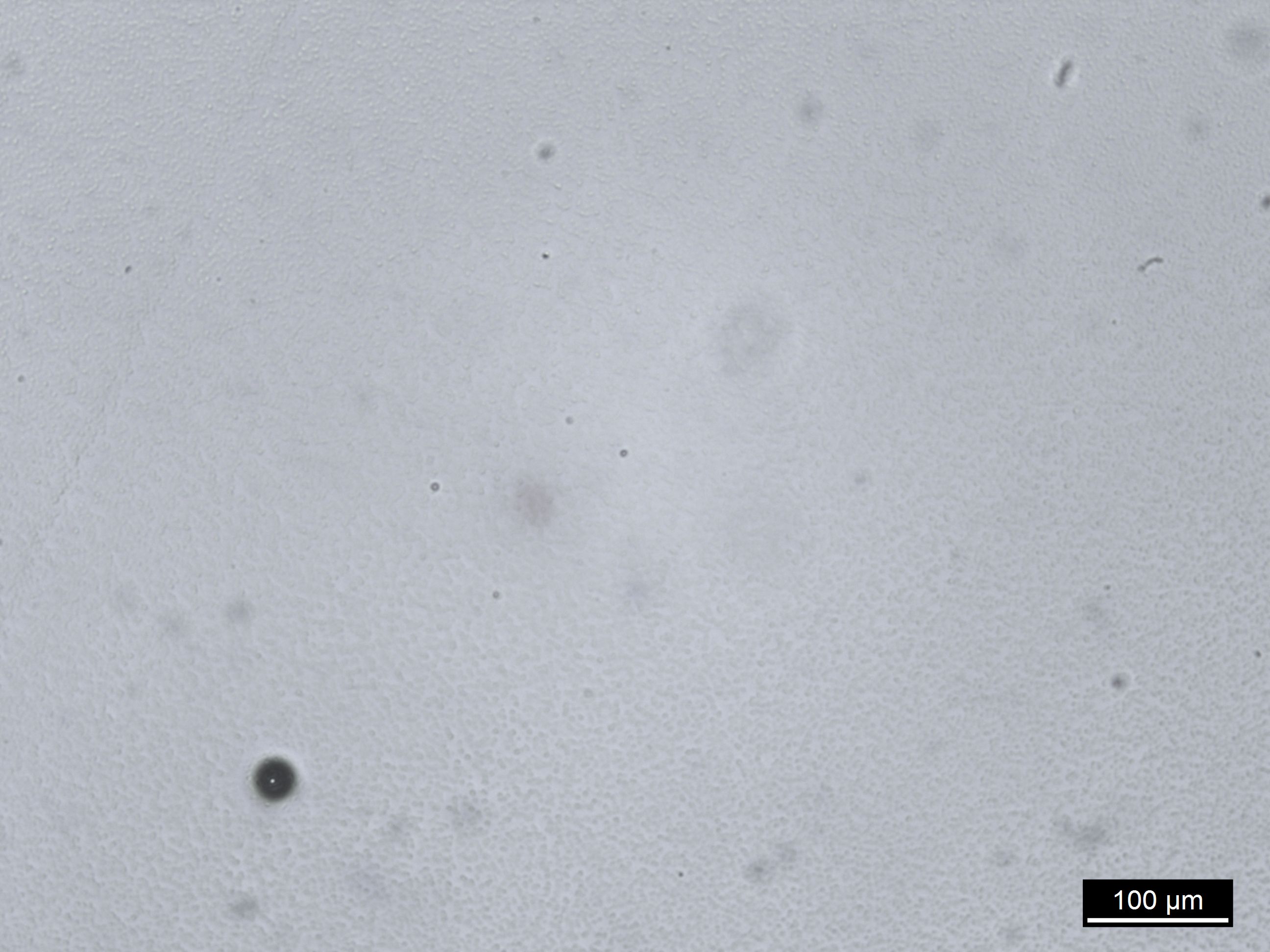

Supplement: Supplementary file 1 [file microorganisms-10-01642-s001.zip › S45_9GU_HD_C.jpg]

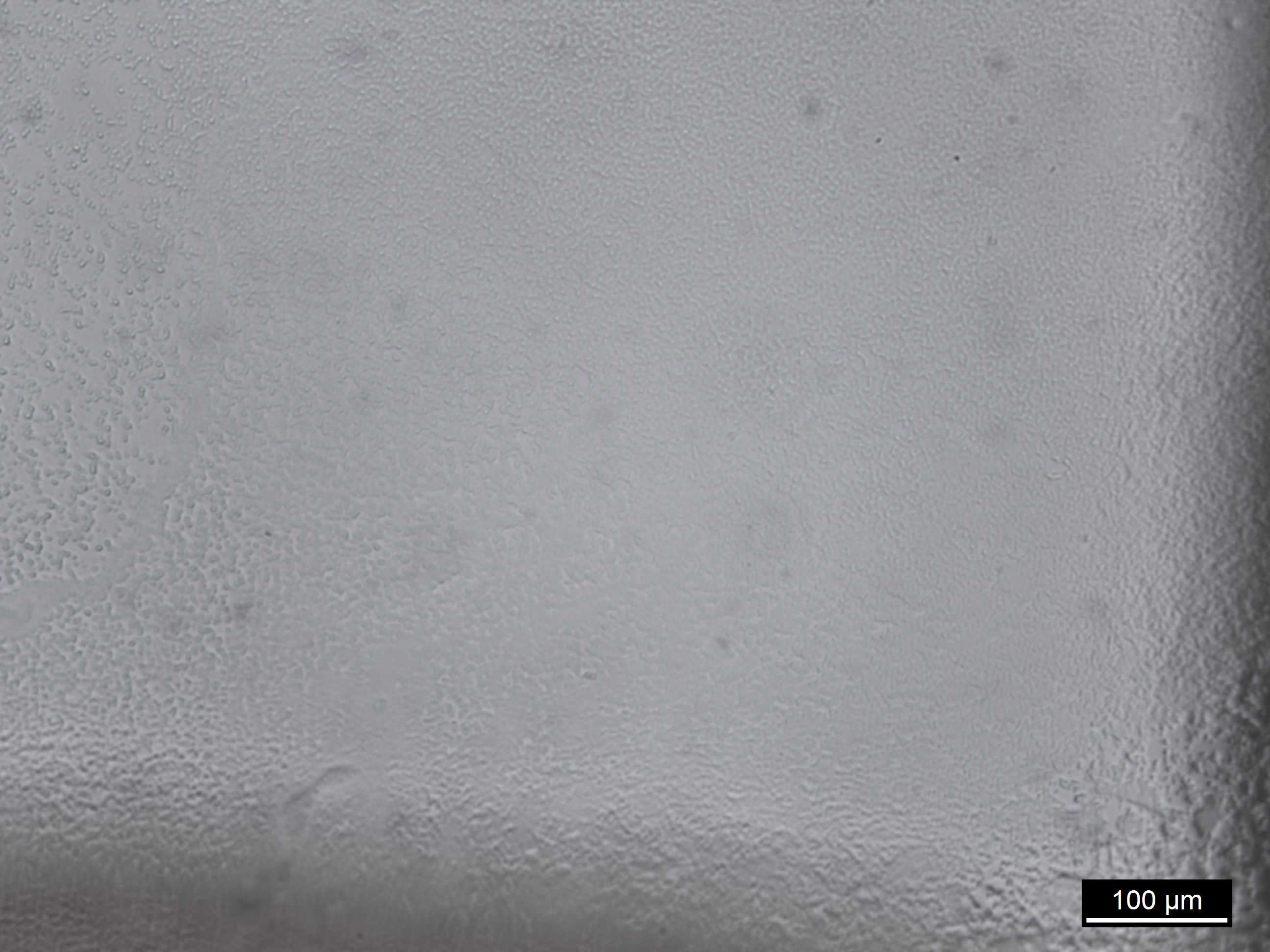

Supplement: Supplementary file 1 [file microorganisms-10-01642-s001.zip › S46_9GU_HD_P.jpg]

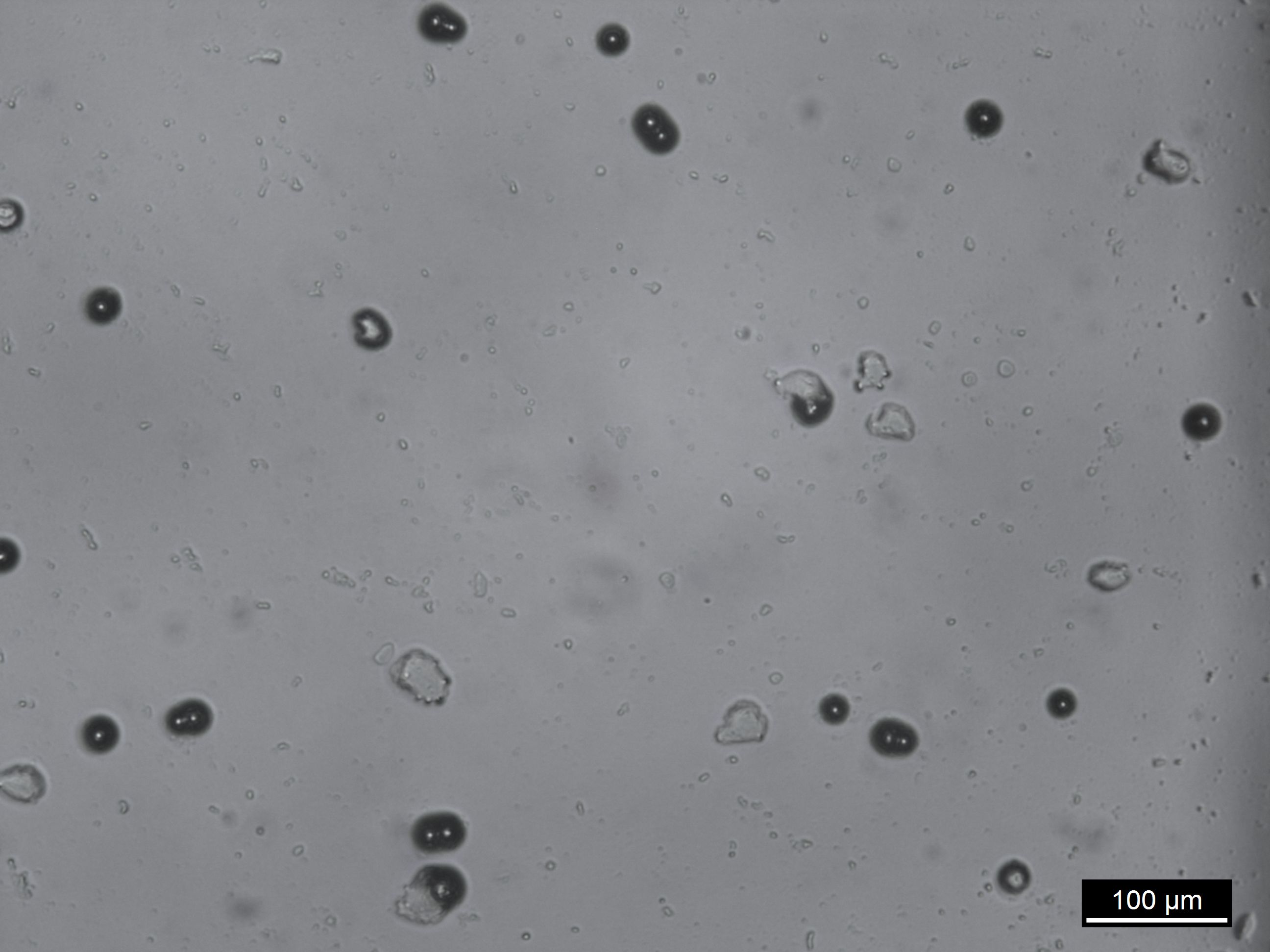

Supplement: Supplementary file 1 [file microorganisms-10-01642-s001.zip › S47_11DS_HD_C.jpg]

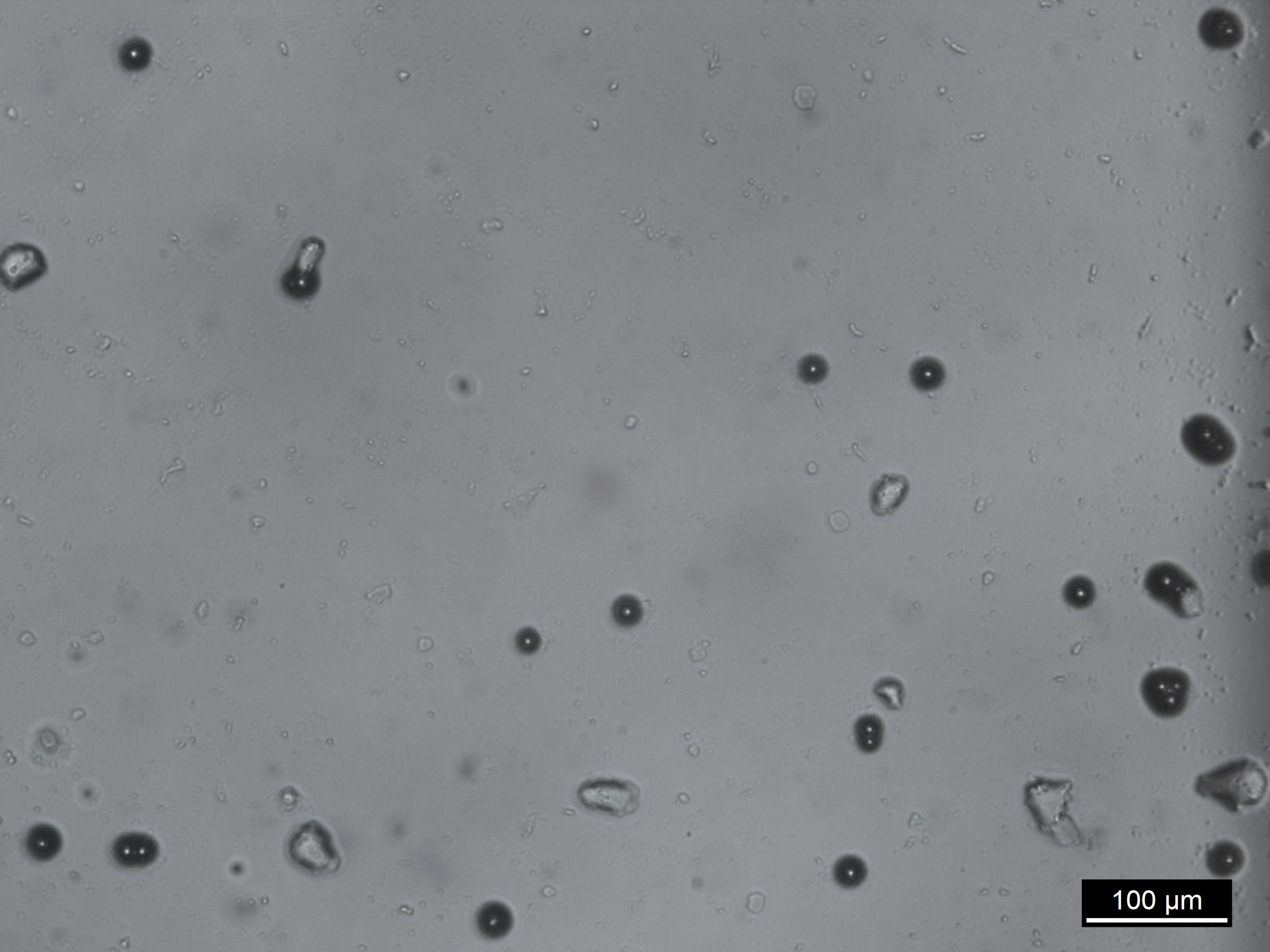

Supplement: Supplementary file 1 [file microorganisms-10-01642-s001.zip › S48_11DS_HD_P.jpg]

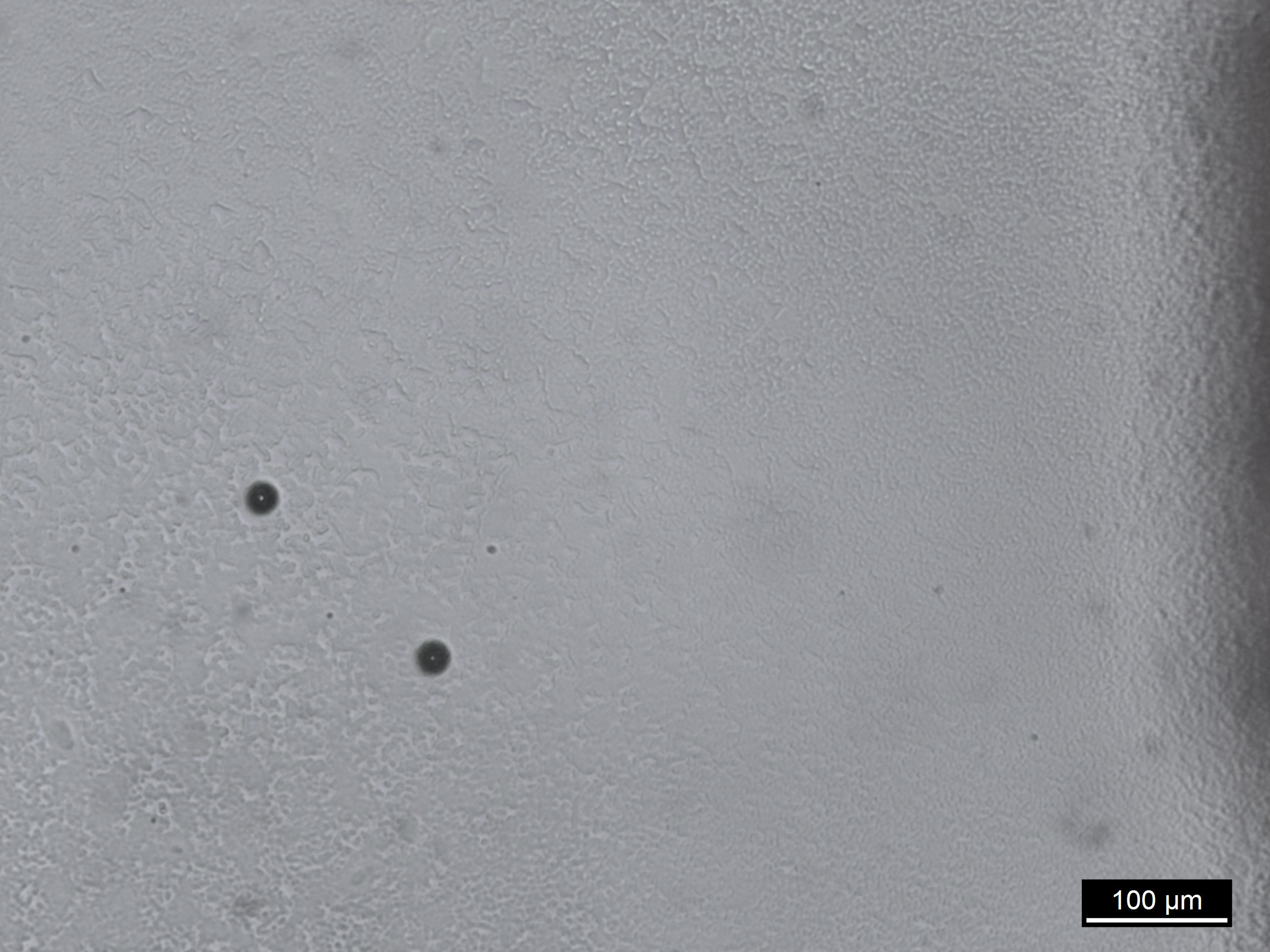

Supplement: Supplementary file 1 [file microorganisms-10-01642-s001.zip › S49_IBU_PD_C.jpg]

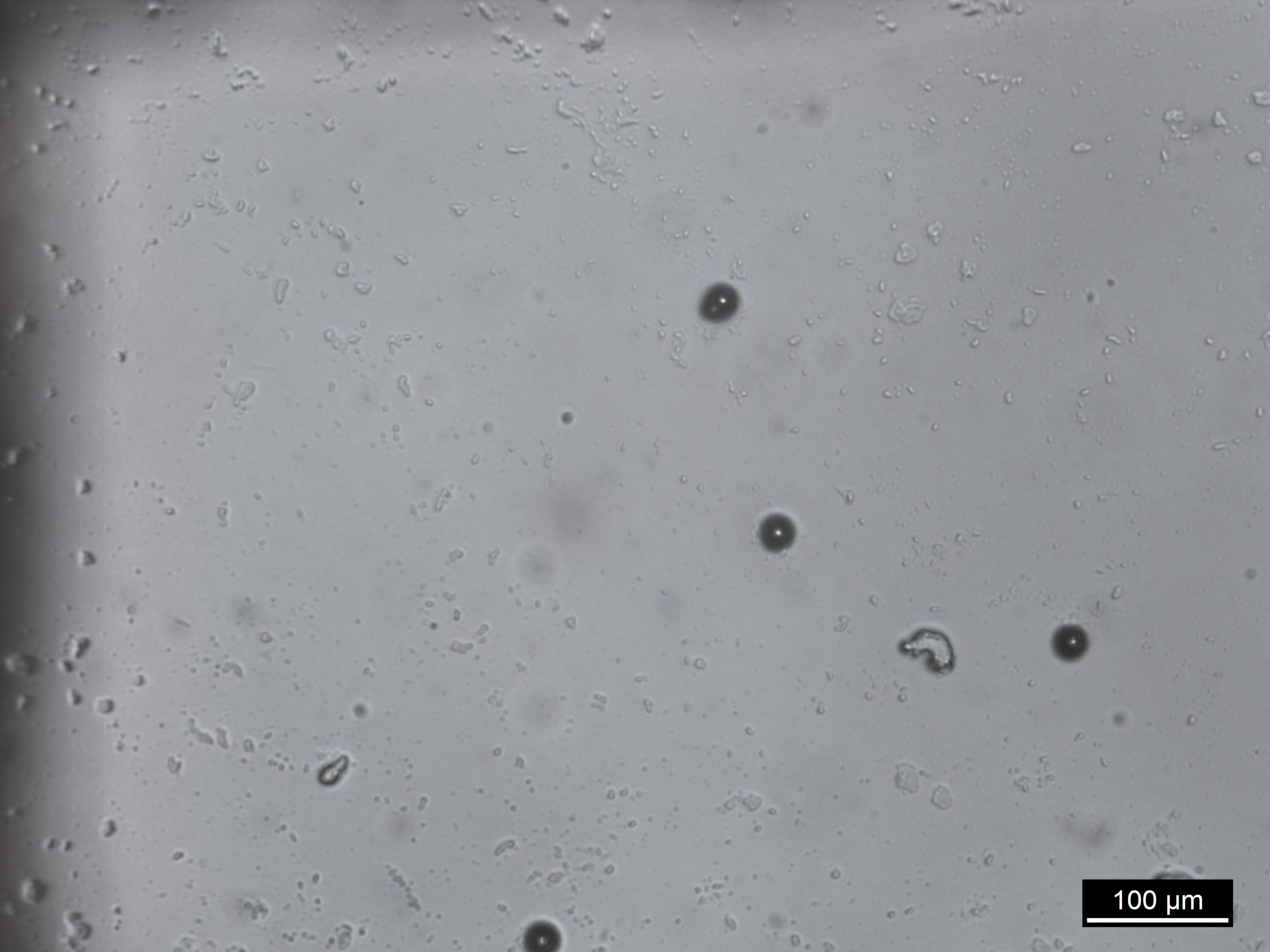

Supplement: Supplementary file 1 [file microorganisms-10-01642-s001.zip › S4_3ST_Control_P.jpg]

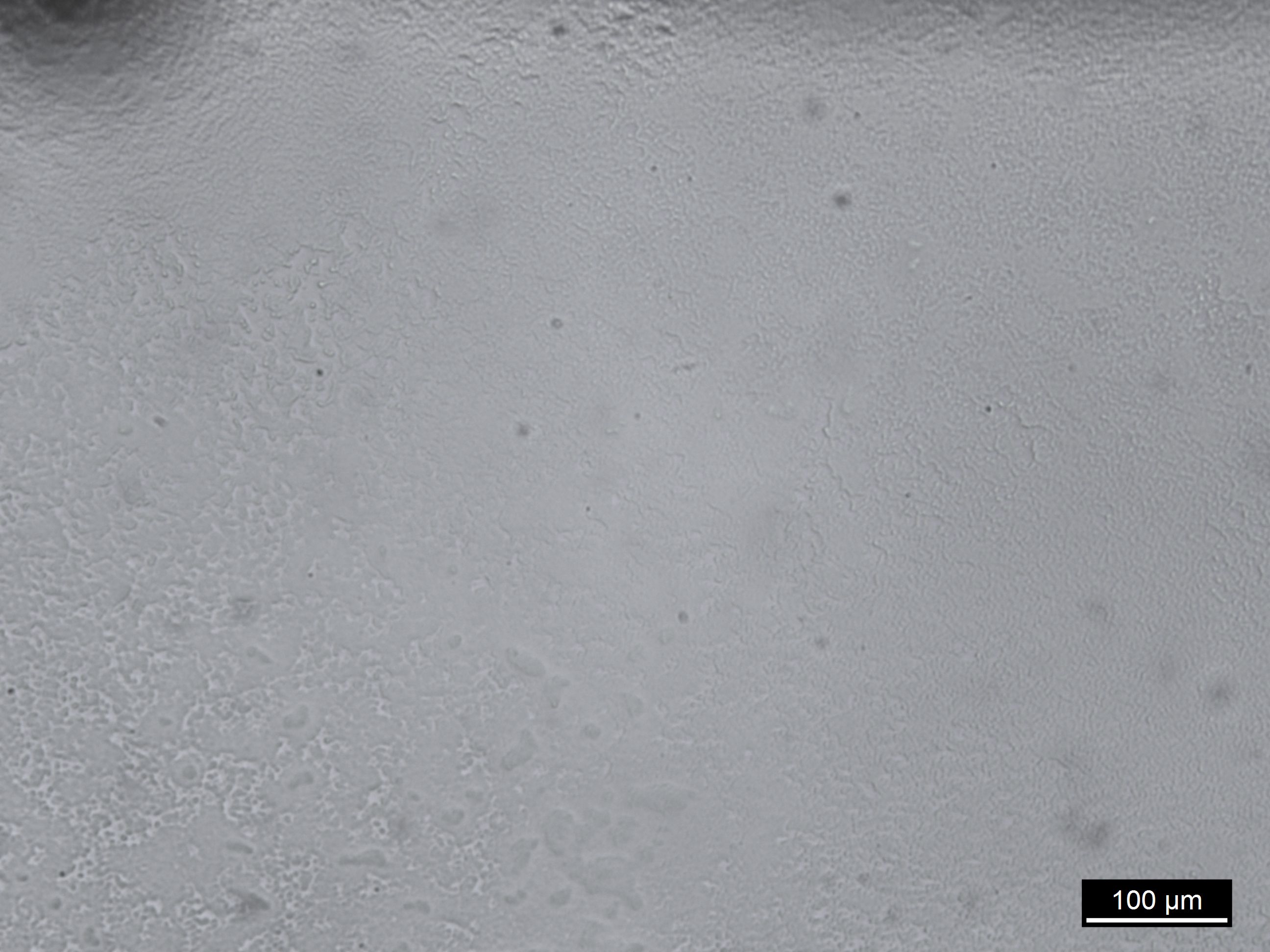

Supplement: Supplementary file 1 [file microorganisms-10-01642-s001.zip › S50_IBU_PD_P.jpg]

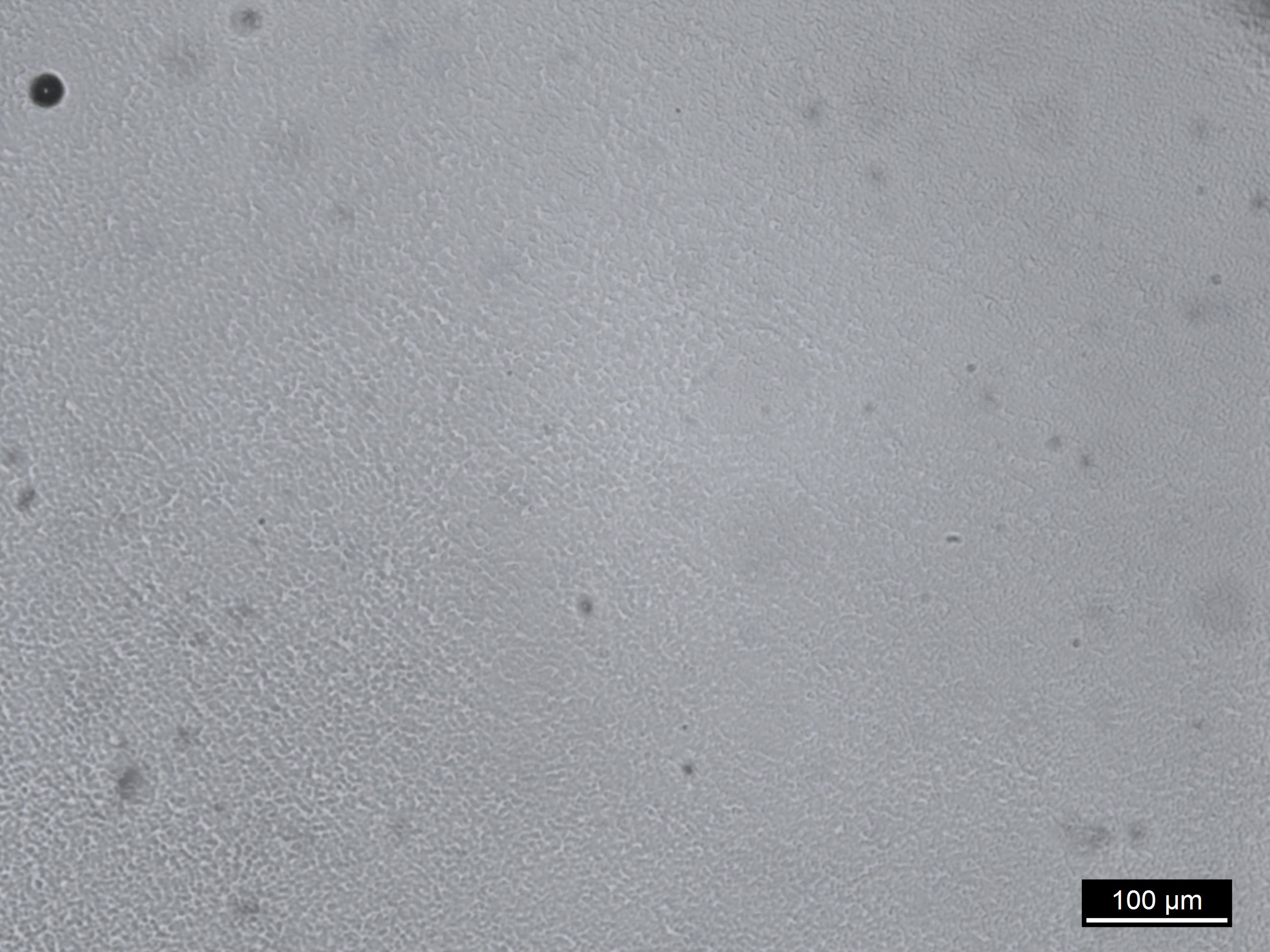

Supplement: Supplementary file 1 [file microorganisms-10-01642-s001.zip › S51_3ST_PD_C.jpg]

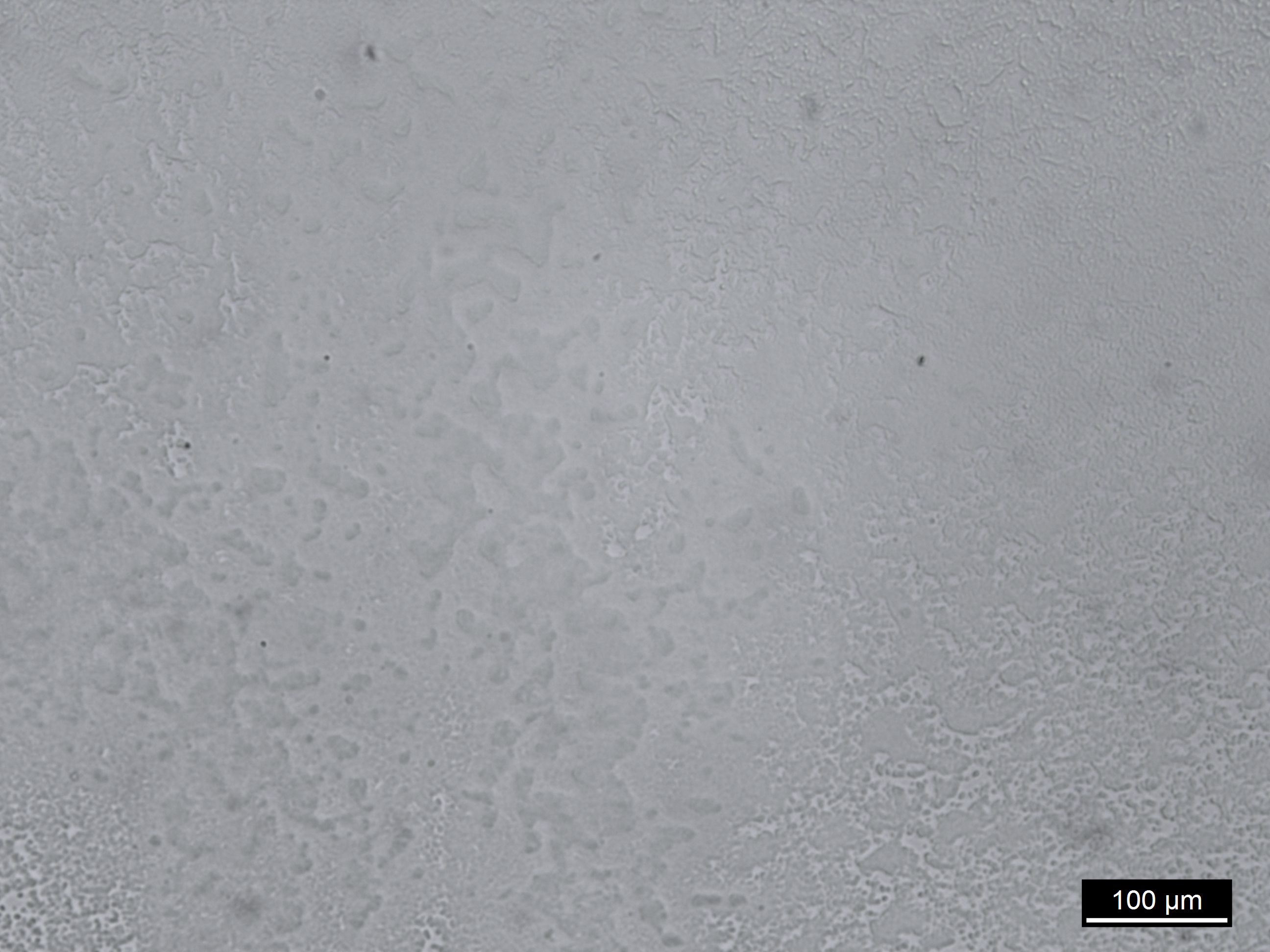

Supplement: Supplementary file 1 [file microorganisms-10-01642-s001.zip › S52_3ST_PD_P.jpg]

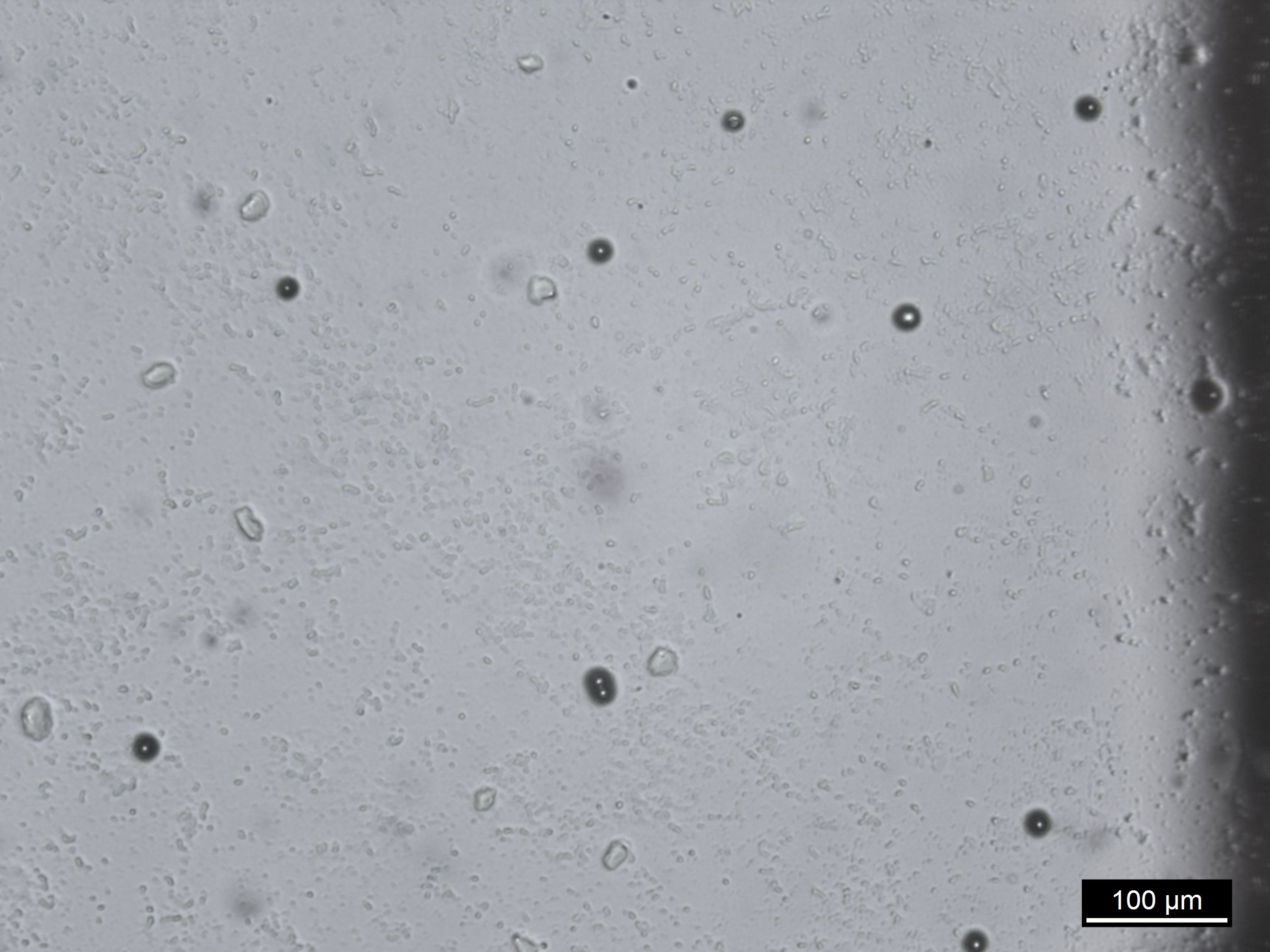

Supplement: Supplementary file 1 [file microorganisms-10-01642-s001.zip › S53_9GU_PD_C.jpg]

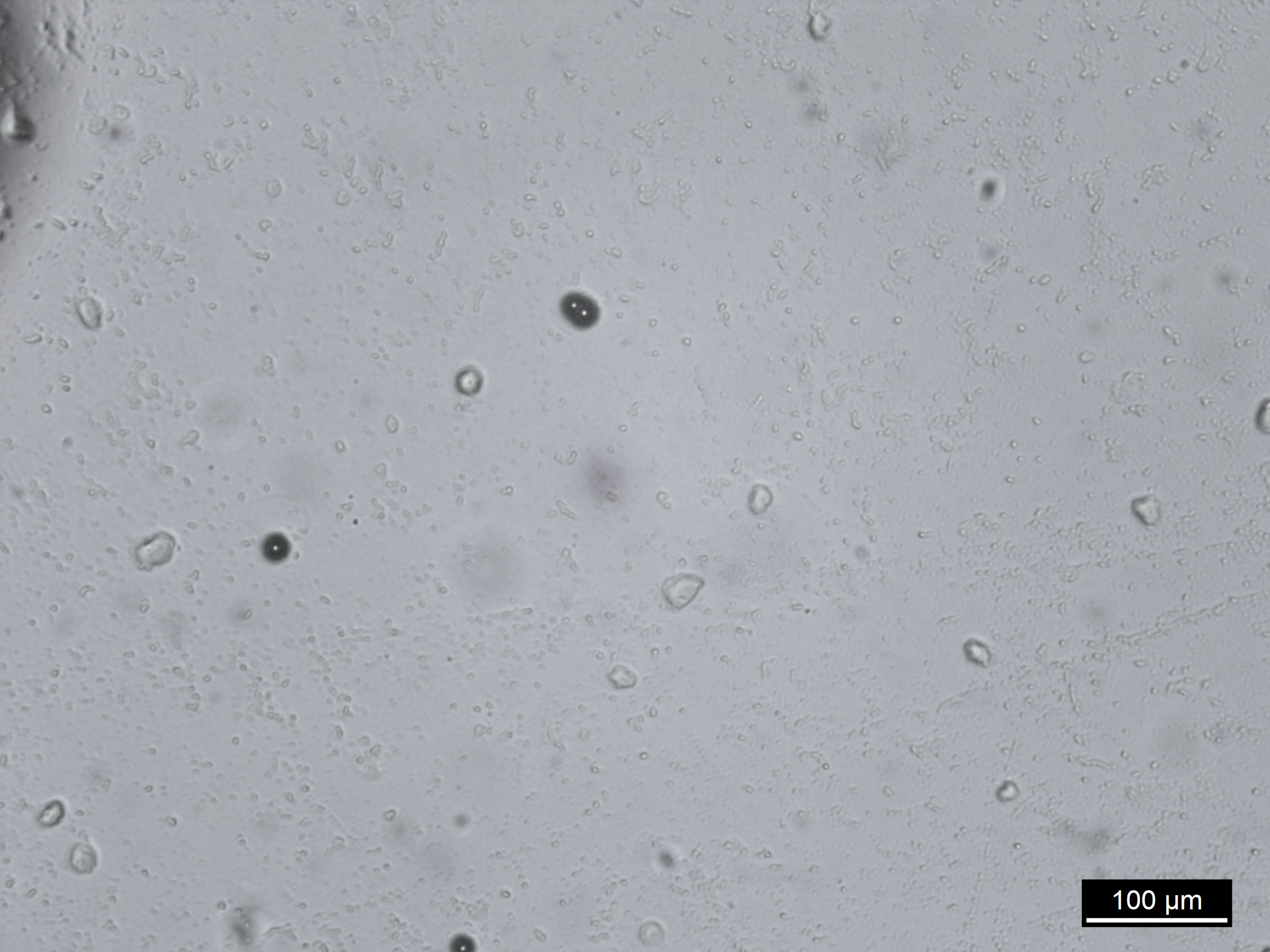

Supplement: Supplementary file 1 [file microorganisms-10-01642-s001.zip › S54_9GU_PD_P.jpg]

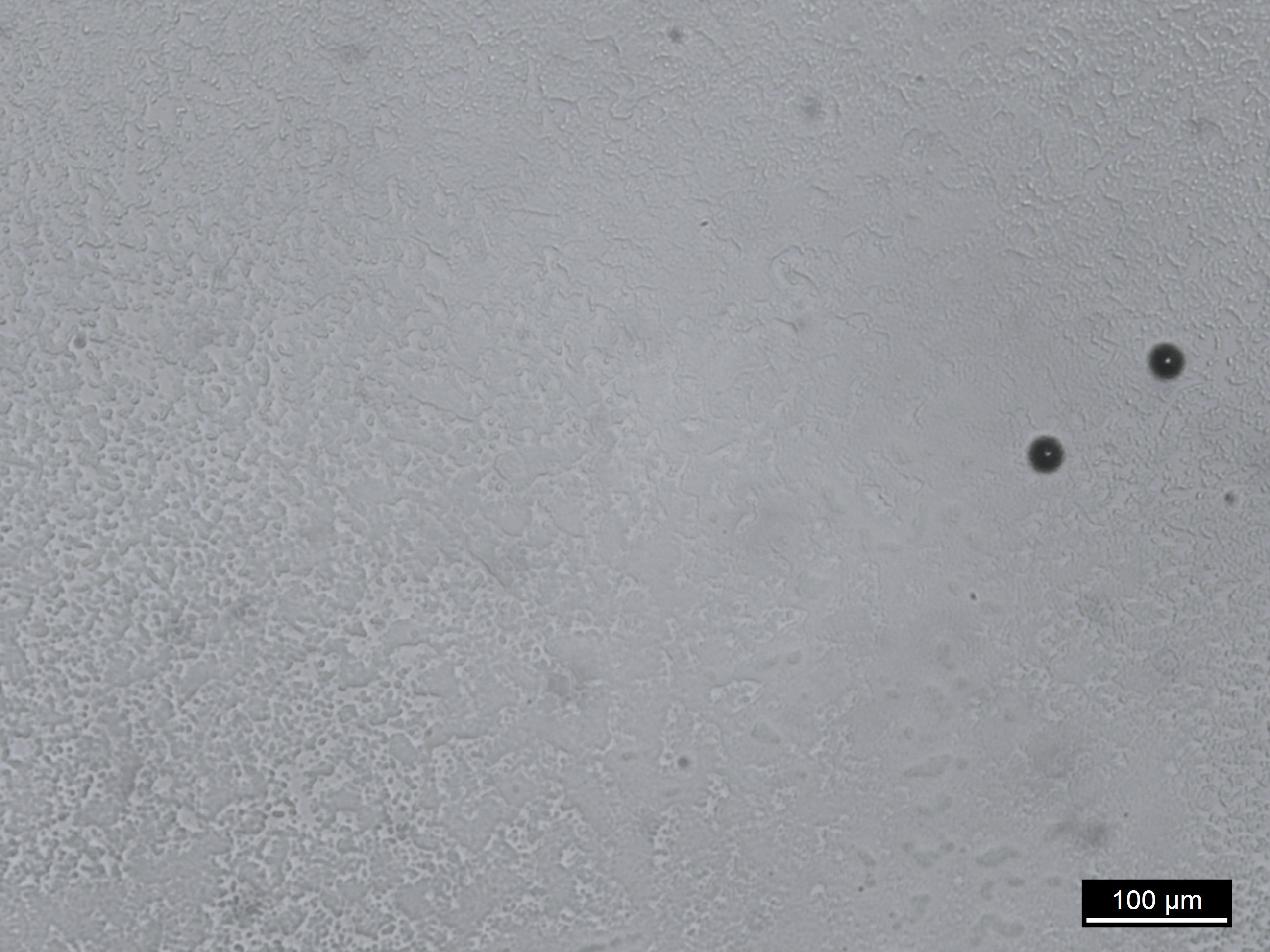

Supplement: Supplementary file 1 [file microorganisms-10-01642-s001.zip › S55_11DS_PD_C.jpg]

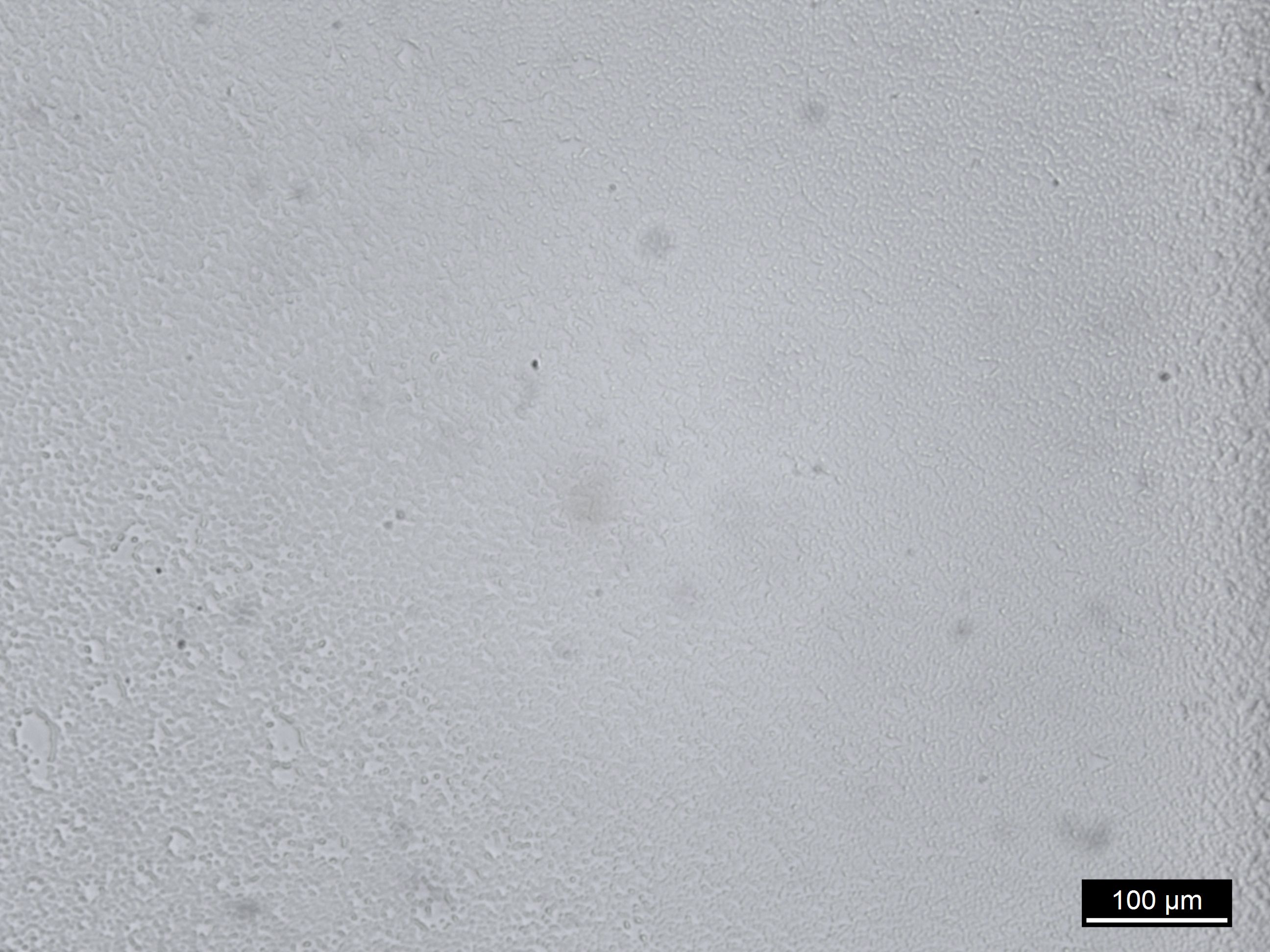

Supplement: Supplementary file 1 [file microorganisms-10-01642-s001.zip › S56_11DS_PD_P.jpg]

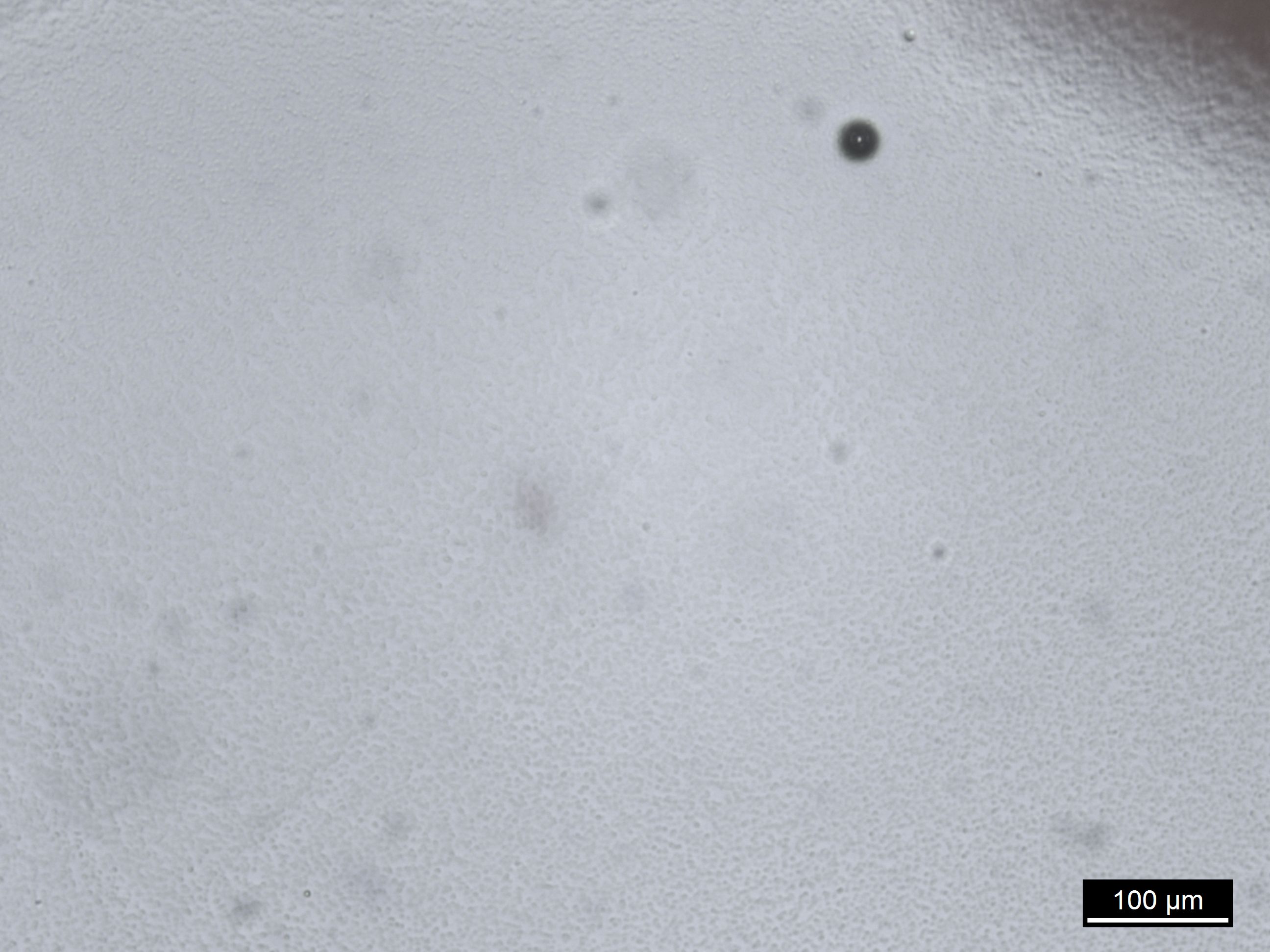

Supplement: Supplementary file 1 [file microorganisms-10-01642-s001.zip › S57_IBU_NM_C.jpg]

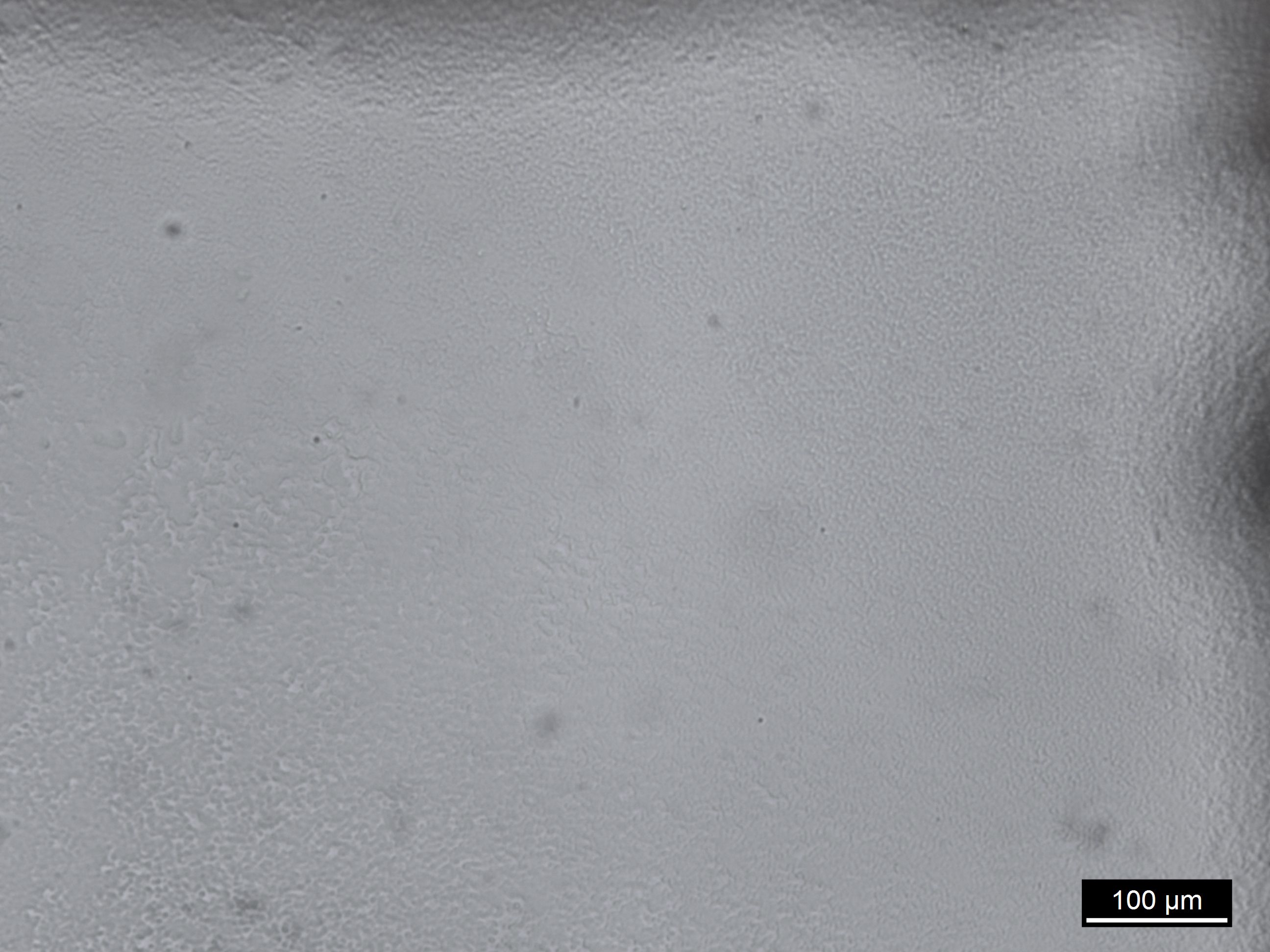

Supplement: Supplementary file 1 [file microorganisms-10-01642-s001.zip › S58_IBU_NM_P.jpg]

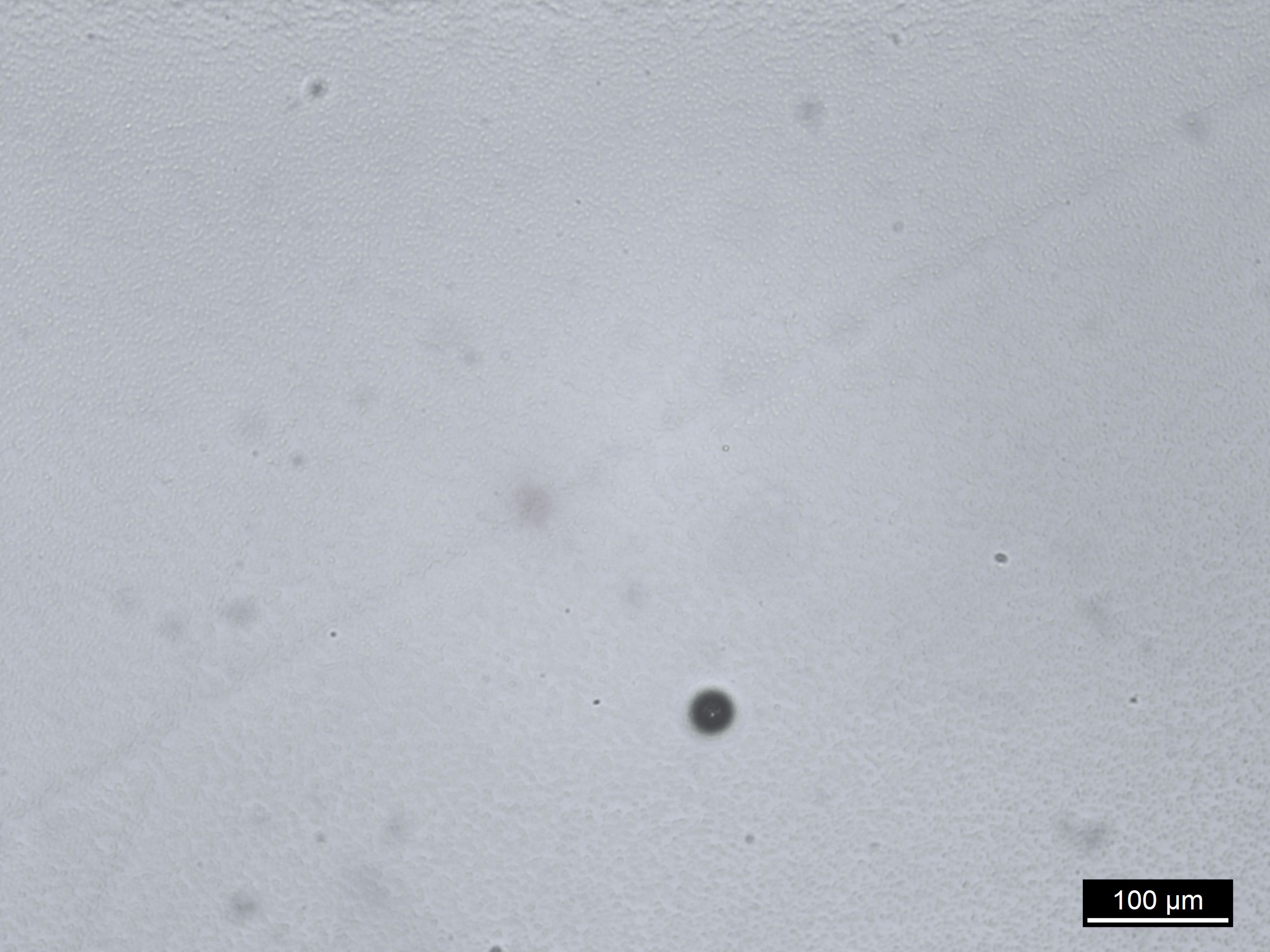

Supplement: Supplementary file 1 [file microorganisms-10-01642-s001.zip › S59_3ST_NM_C.jpg]

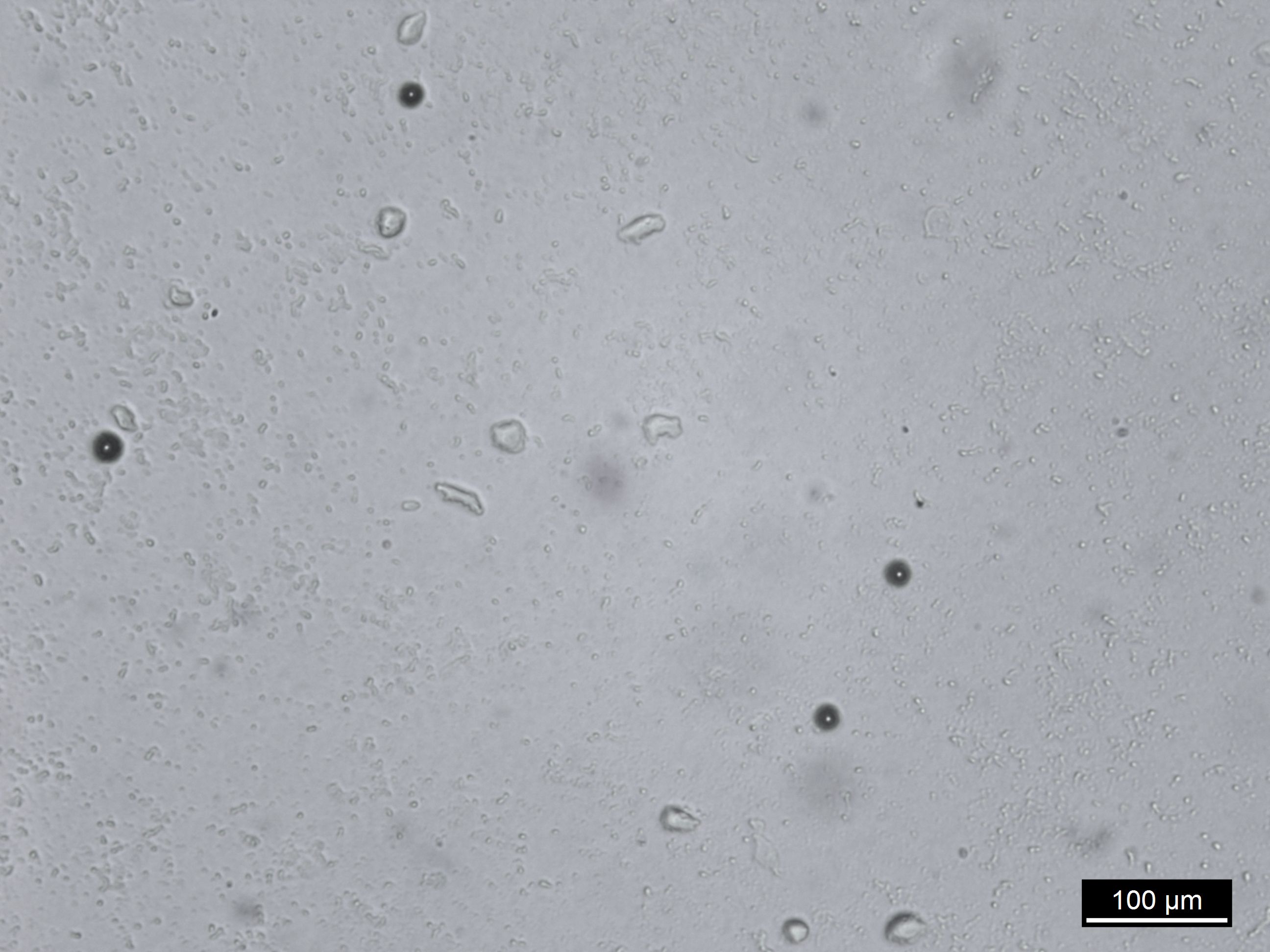

Supplement: Supplementary file 1 [file microorganisms-10-01642-s001.zip › S5_9GU_Control_C.jpg]

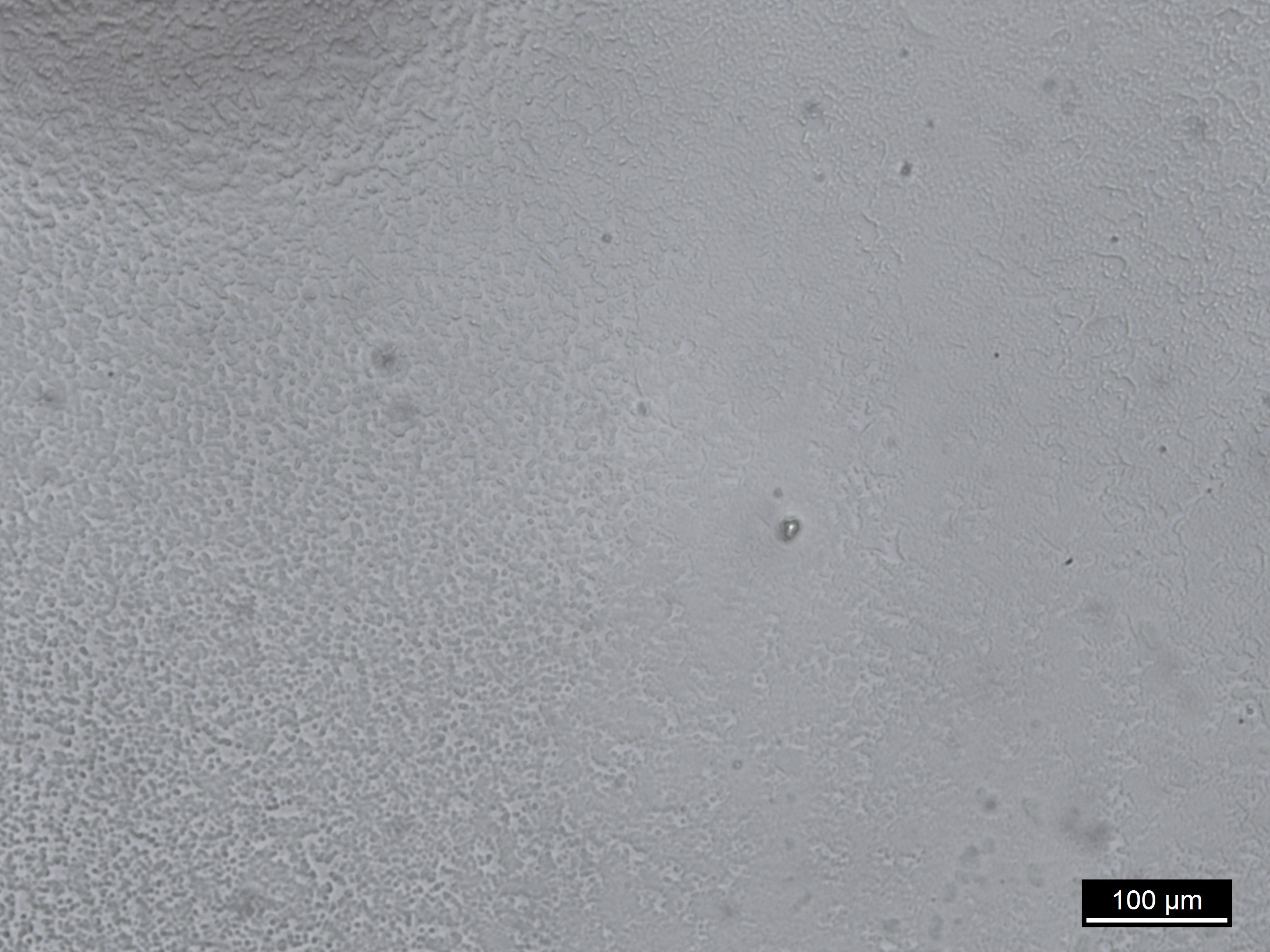

Supplement: Supplementary file 1 [file microorganisms-10-01642-s001.zip › S60_3ST_NM_P.jpg]

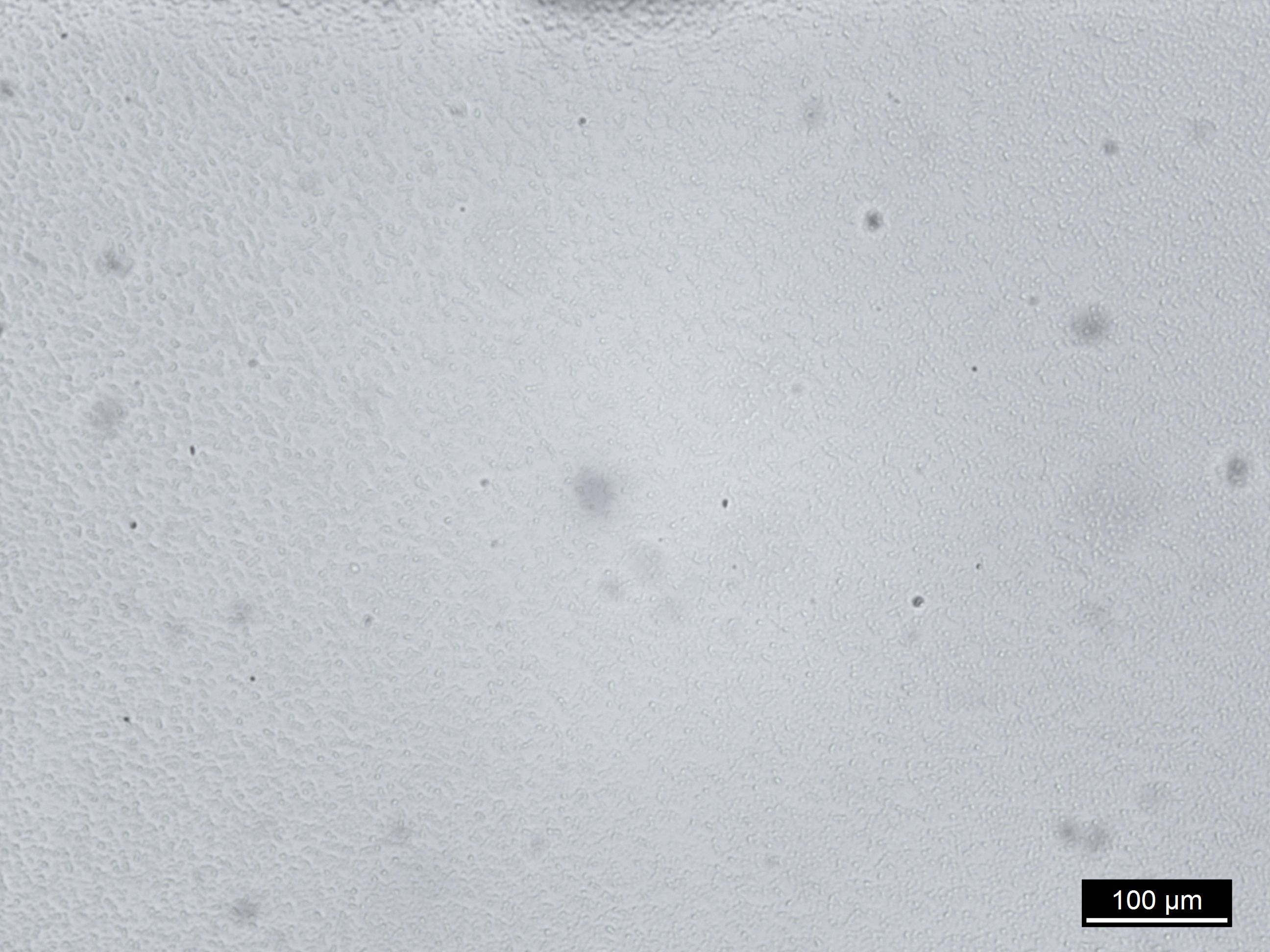

Supplement: Supplementary file 1 [file microorganisms-10-01642-s001.zip › S62_9GU_NM_P.jpg]

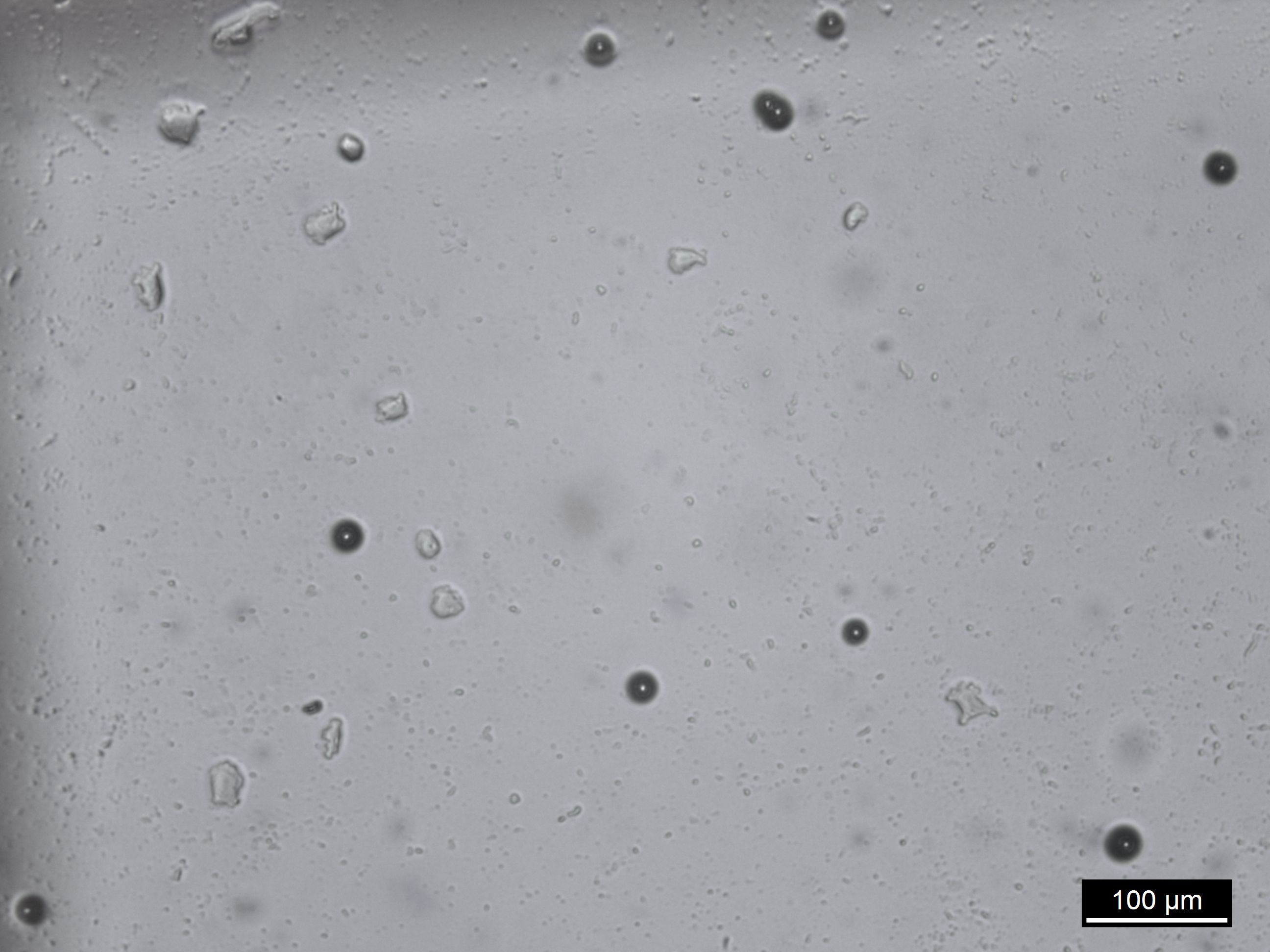

Supplement: Supplementary file 1 [file microorganisms-10-01642-s001.zip › S63_11DS_NM_C.jpg]

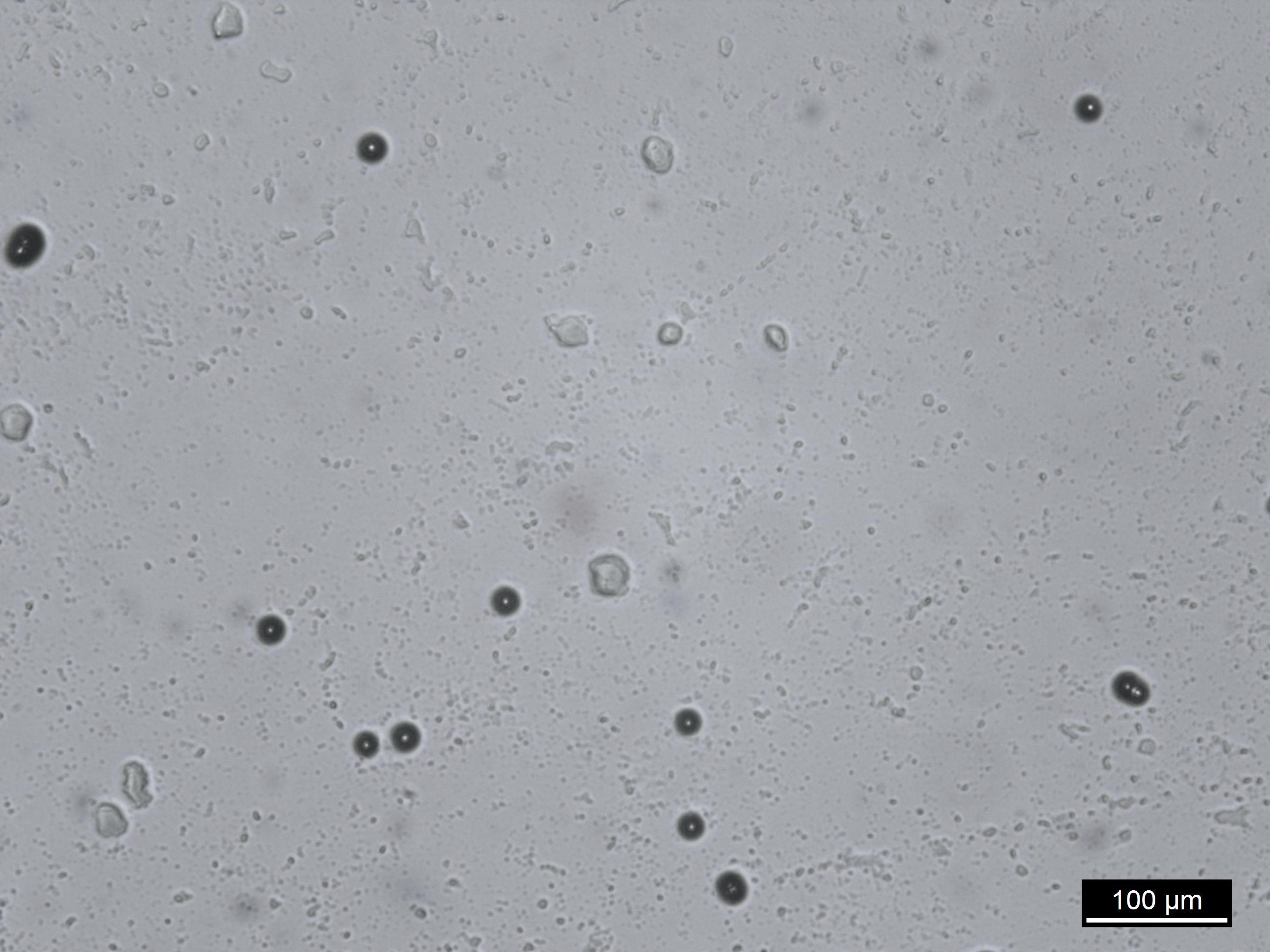

Supplement: Supplementary file 1 [file microorganisms-10-01642-s001.zip › S64_11DS_NM_P.jpg]

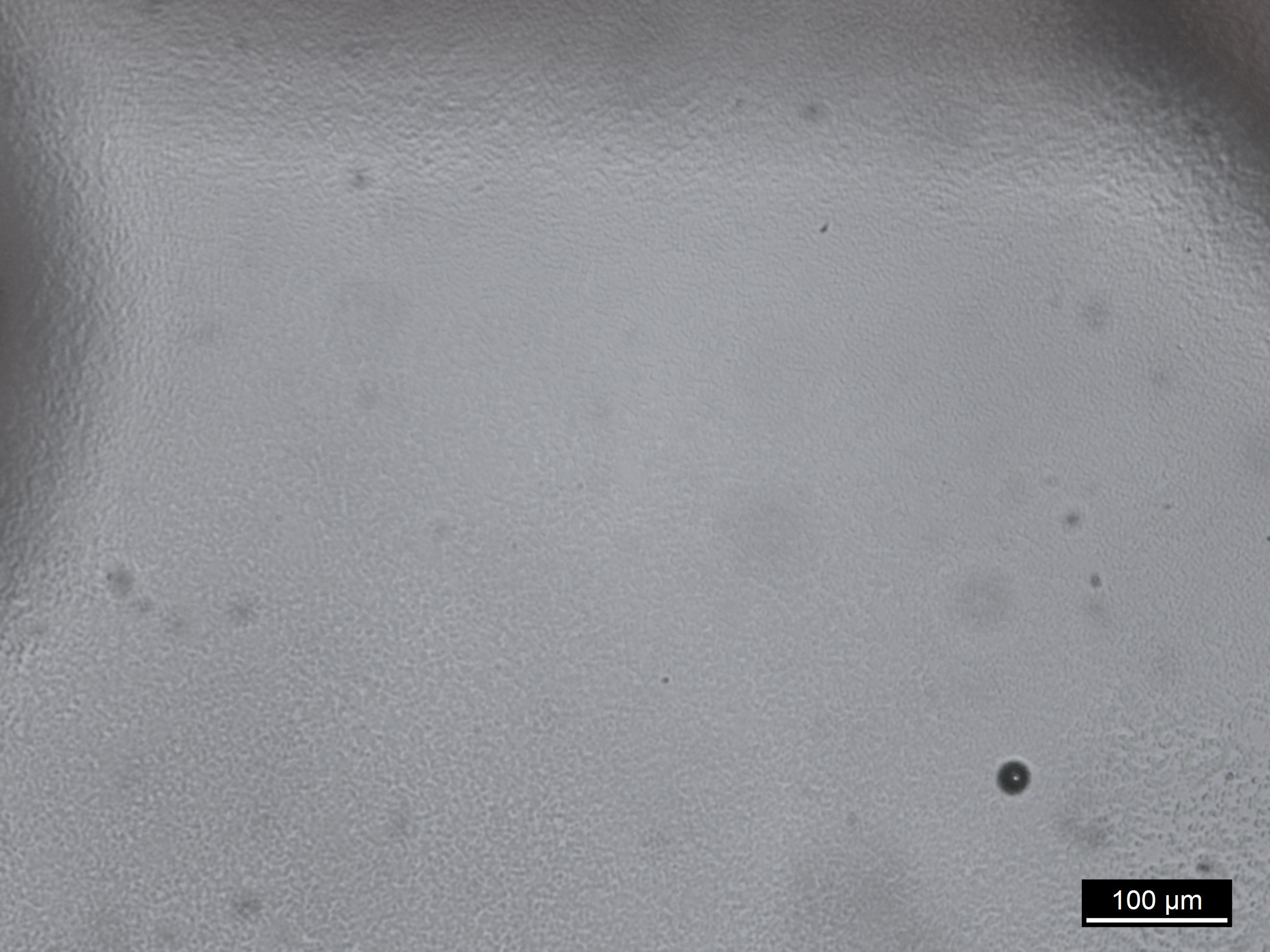

Supplement: Supplementary file 1 [file microorganisms-10-01642-s001.zip › S65_IBU_PVPI_C.jpg]

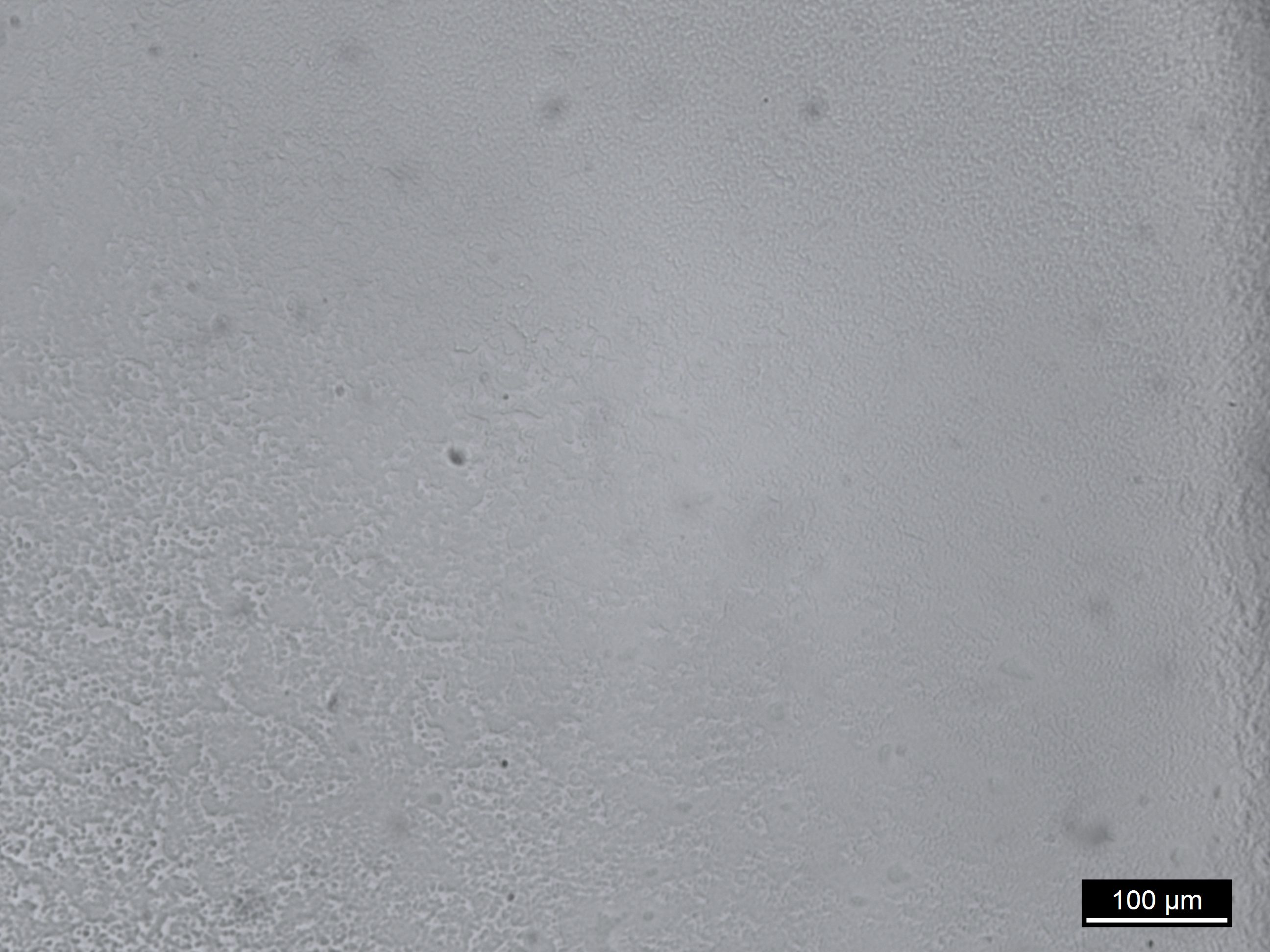

Supplement: Supplementary file 1 [file microorganisms-10-01642-s001.zip › S66_IBU_PVPI_P.jpg]

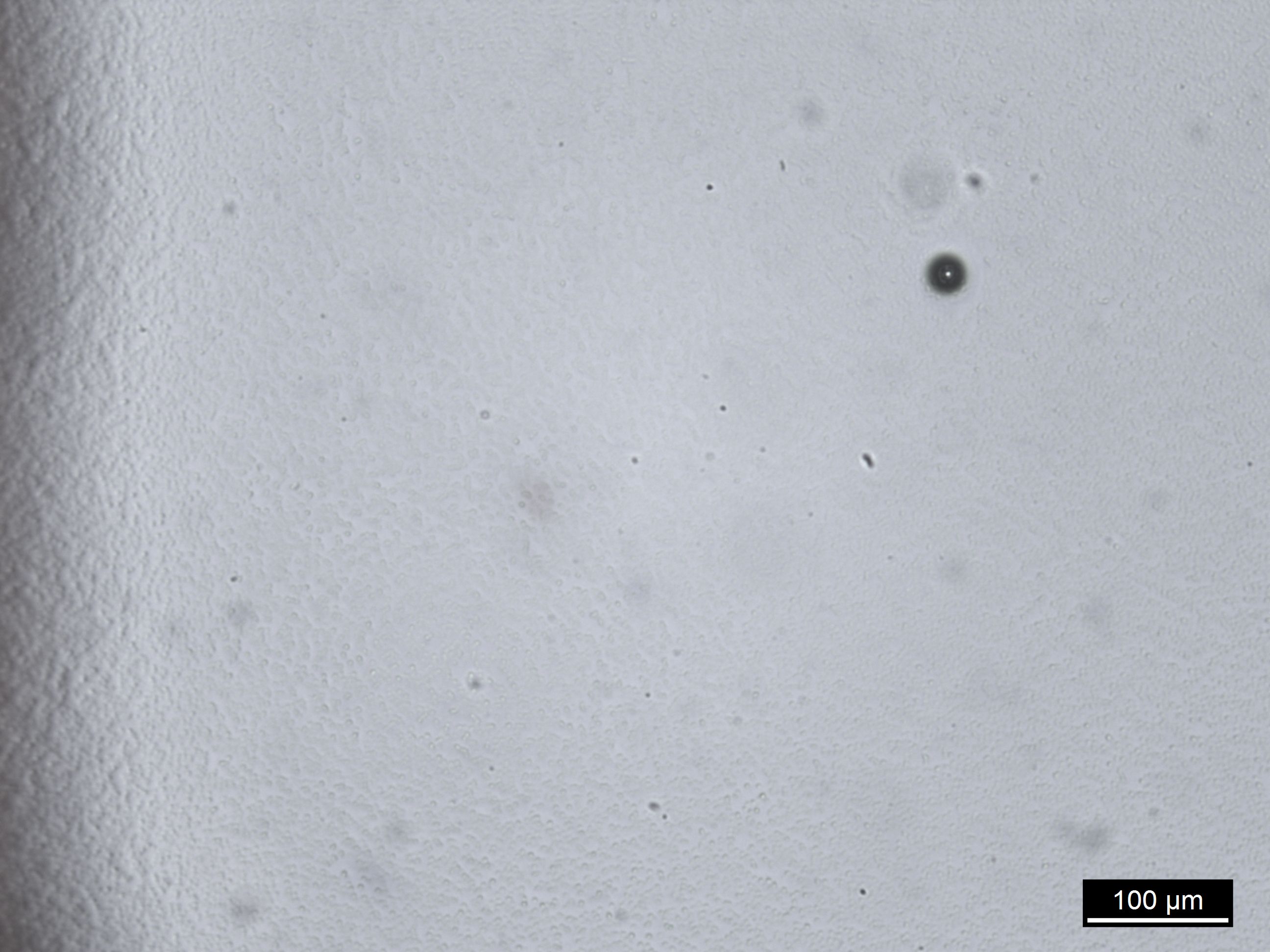

Supplement: Supplementary file 1 [file microorganisms-10-01642-s001.zip › S67_3ST_PVPI_C.jpg]

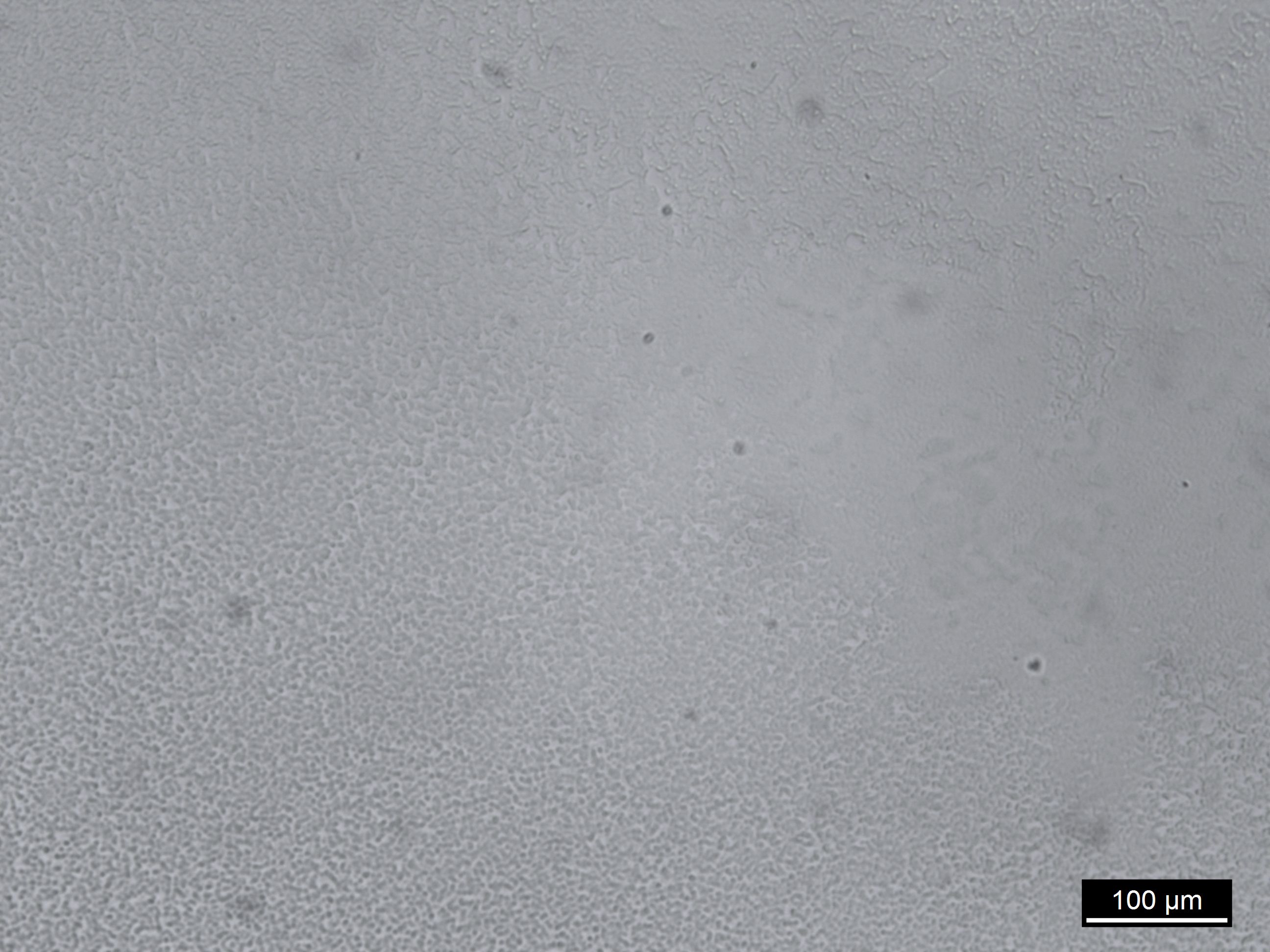

Supplement: Supplementary file 1 [file microorganisms-10-01642-s001.zip › S68_3ST_PVPI_P.jpg]

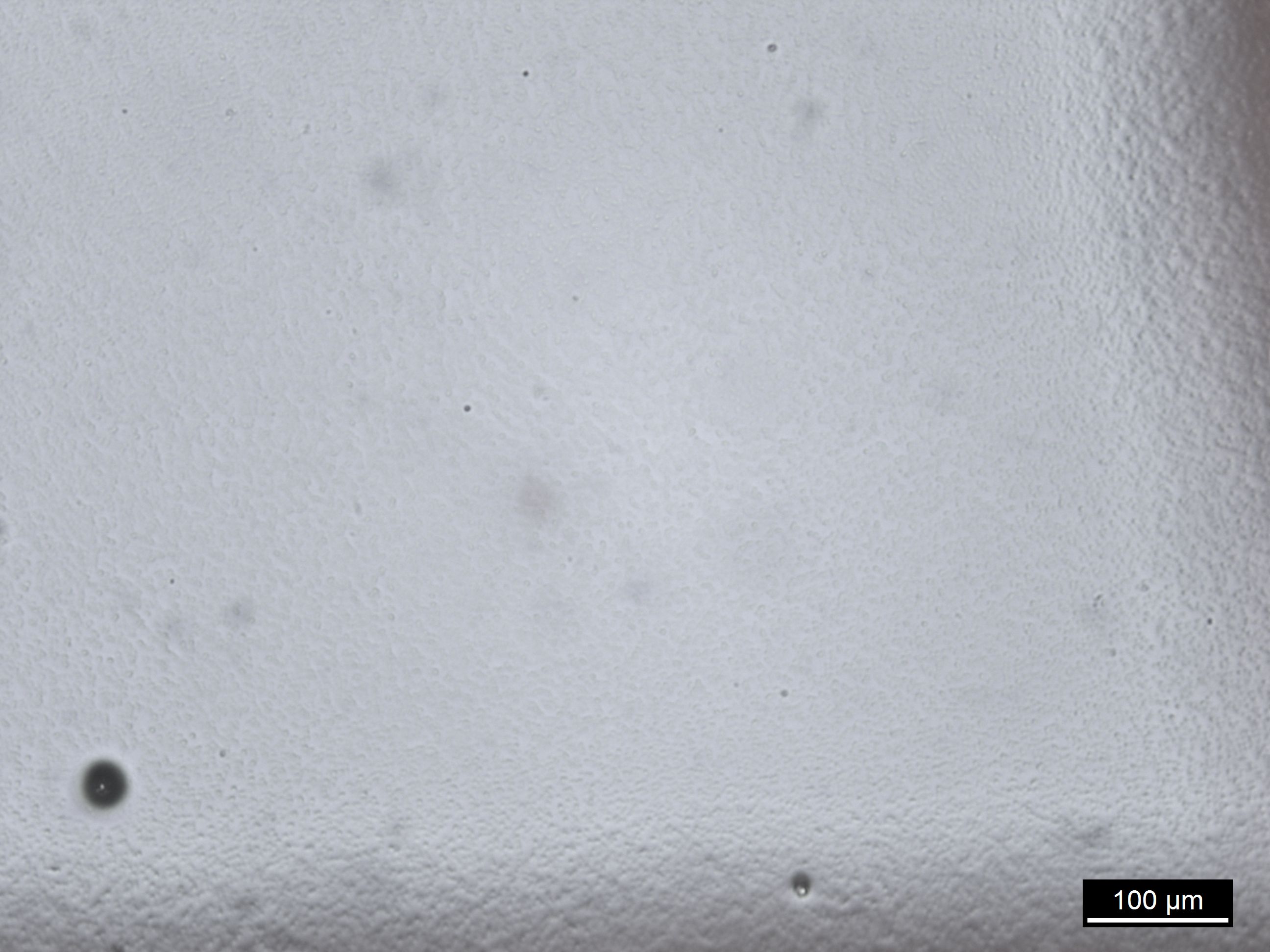

Supplement: Supplementary file 1 [file microorganisms-10-01642-s001.zip › S69_9GU_PVPI_C.jpg]

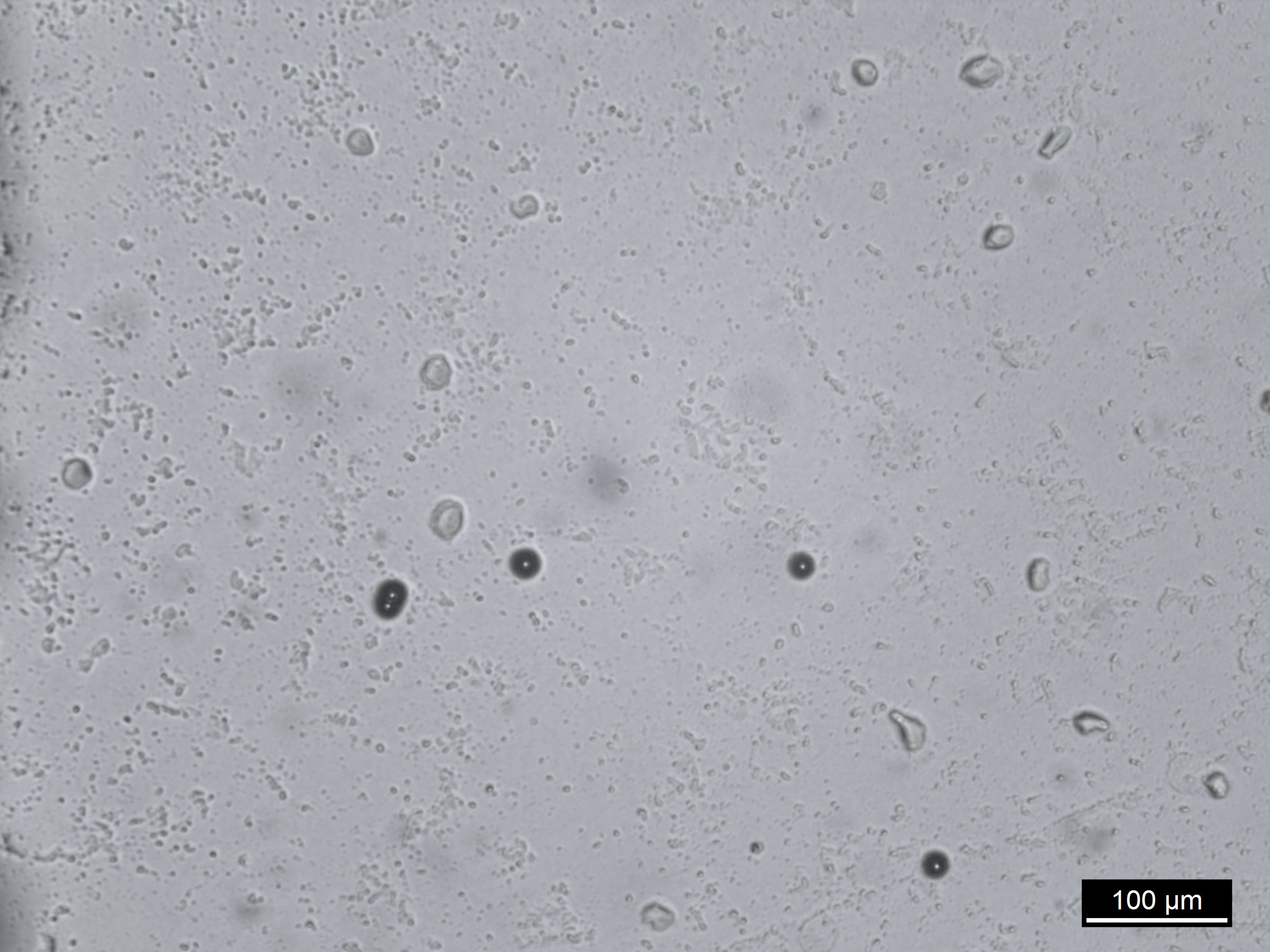

Supplement: Supplementary file 1 [file microorganisms-10-01642-s001.zip › S6_9GU_Control_P.jpg]

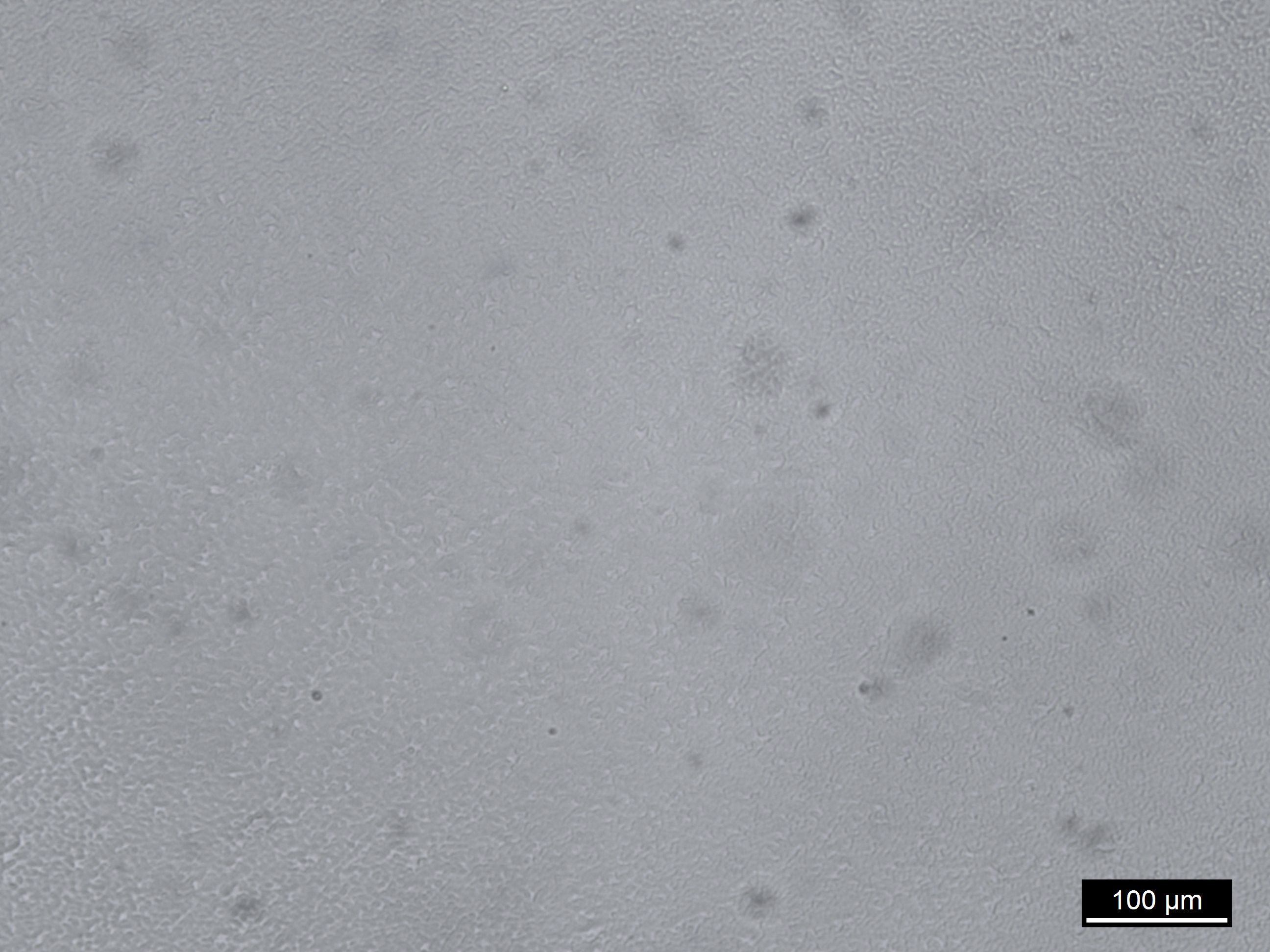

Supplement: Supplementary file 1 [file microorganisms-10-01642-s001.zip › S70_9GU_PVPI_P.jpg]

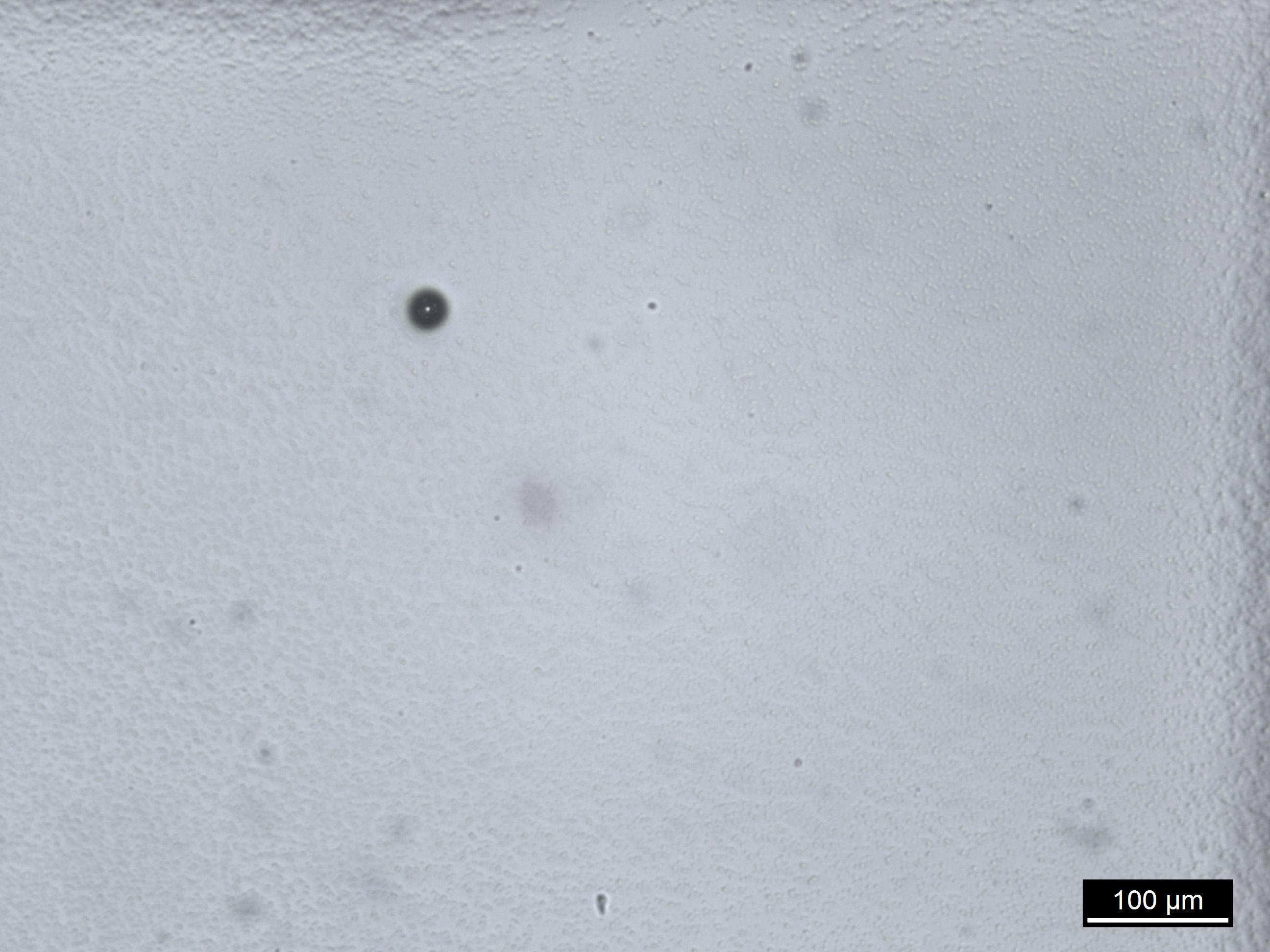

Supplement: Supplementary file 1 [file microorganisms-10-01642-s001.zip › S71_11DS_PVPI_C.jpg]

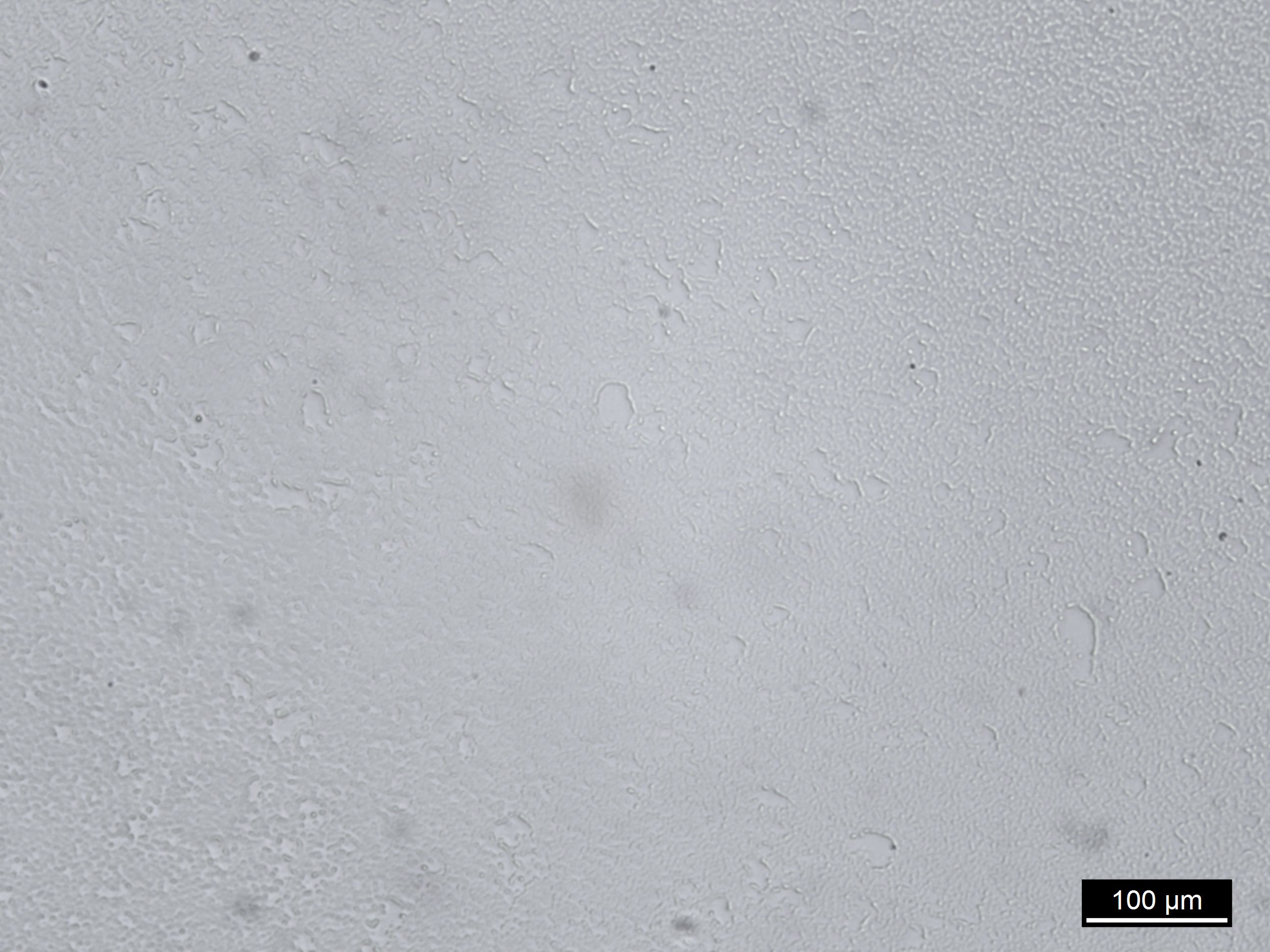

Supplement: Supplementary file 1 [file microorganisms-10-01642-s001.zip › S72_11DS_PVPI_P.jpg]

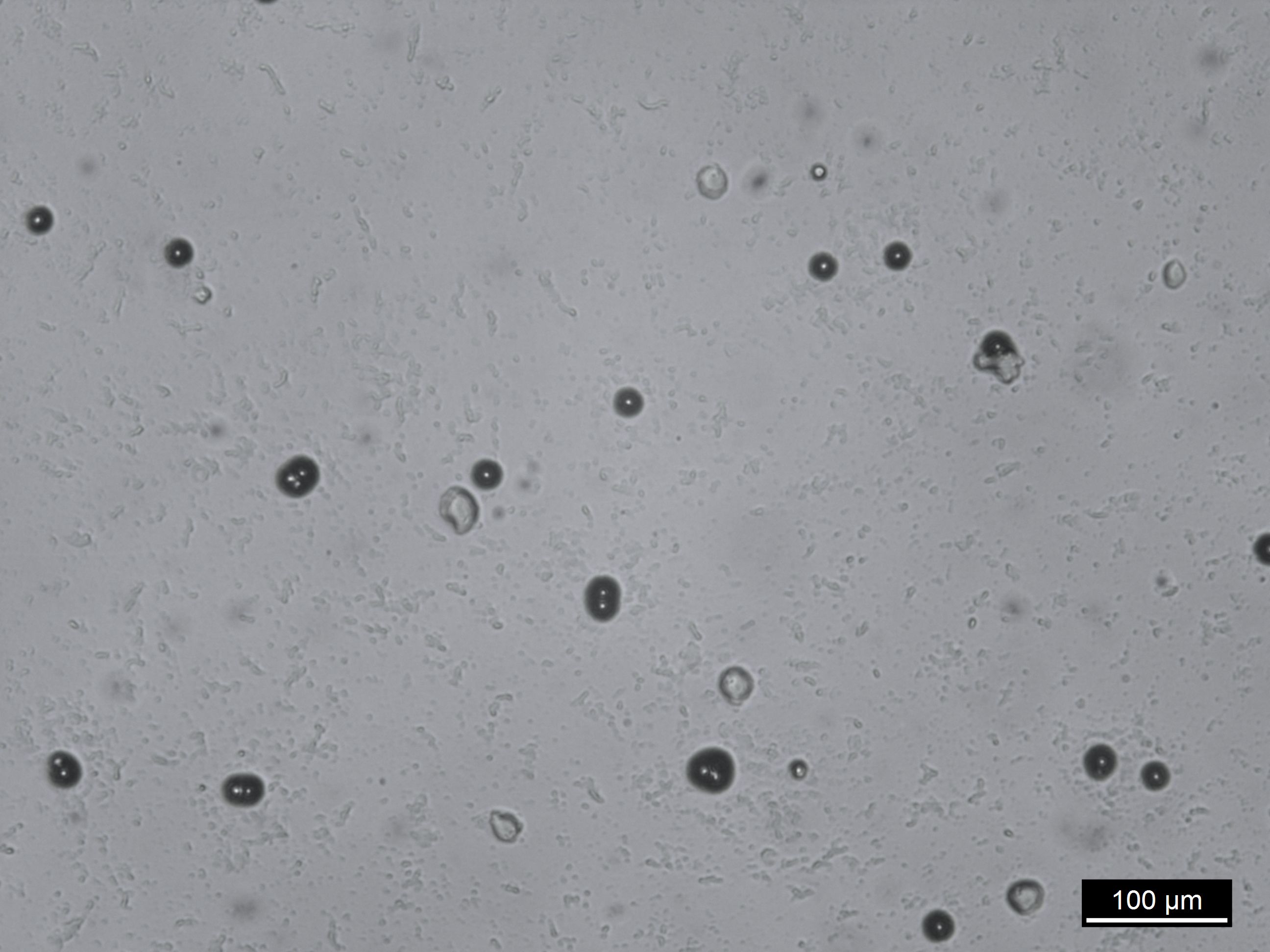

Supplement: Supplementary file 1 [file microorganisms-10-01642-s001.zip › S73_IBU_MF_C.jpg]

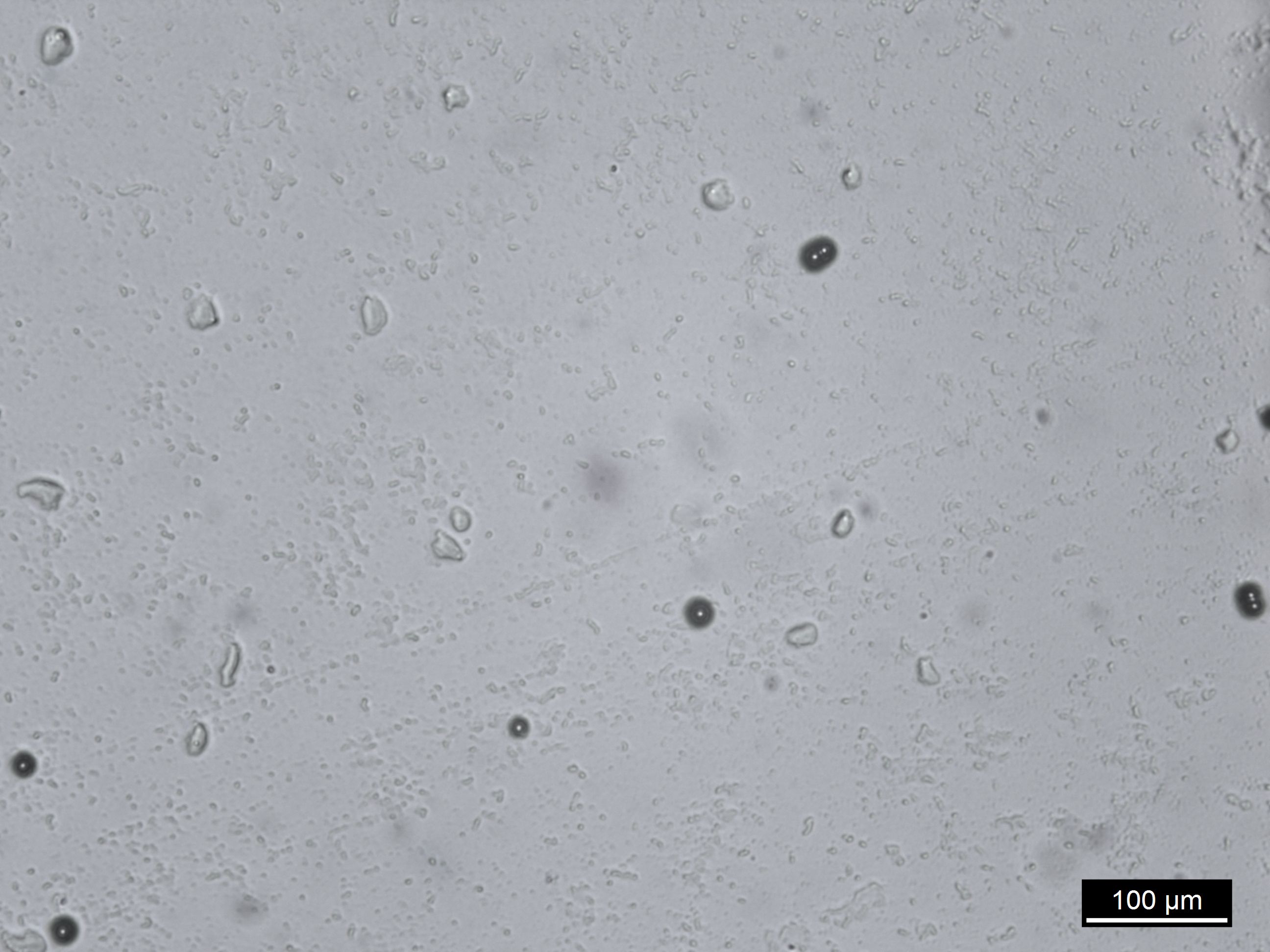

Supplement: Supplementary file 1 [file microorganisms-10-01642-s001.zip › S74_IBU_MF_P.jpg]

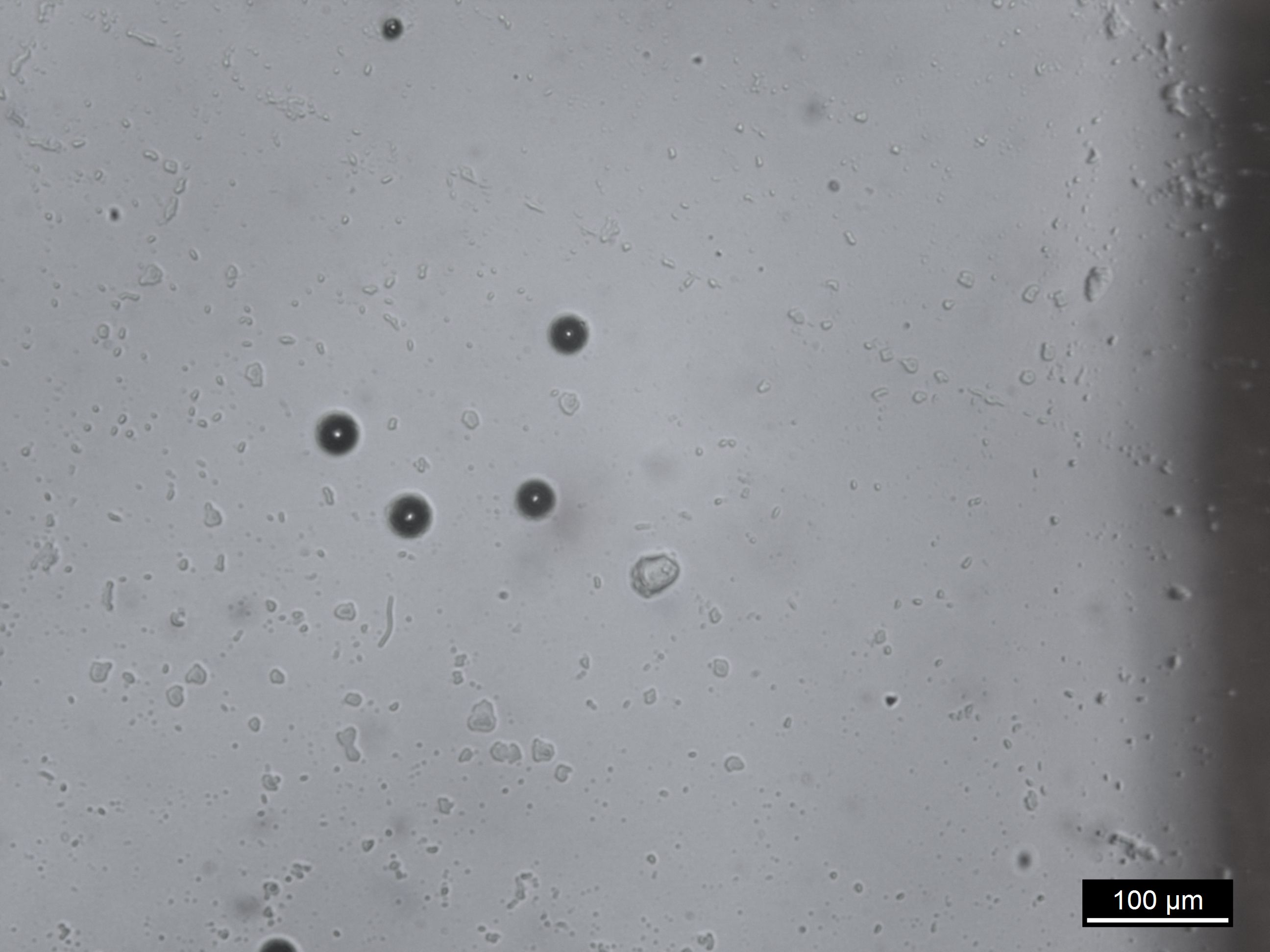

Supplement: Supplementary file 1 [file microorganisms-10-01642-s001.zip › S75_3ST_MF_C.jpg]

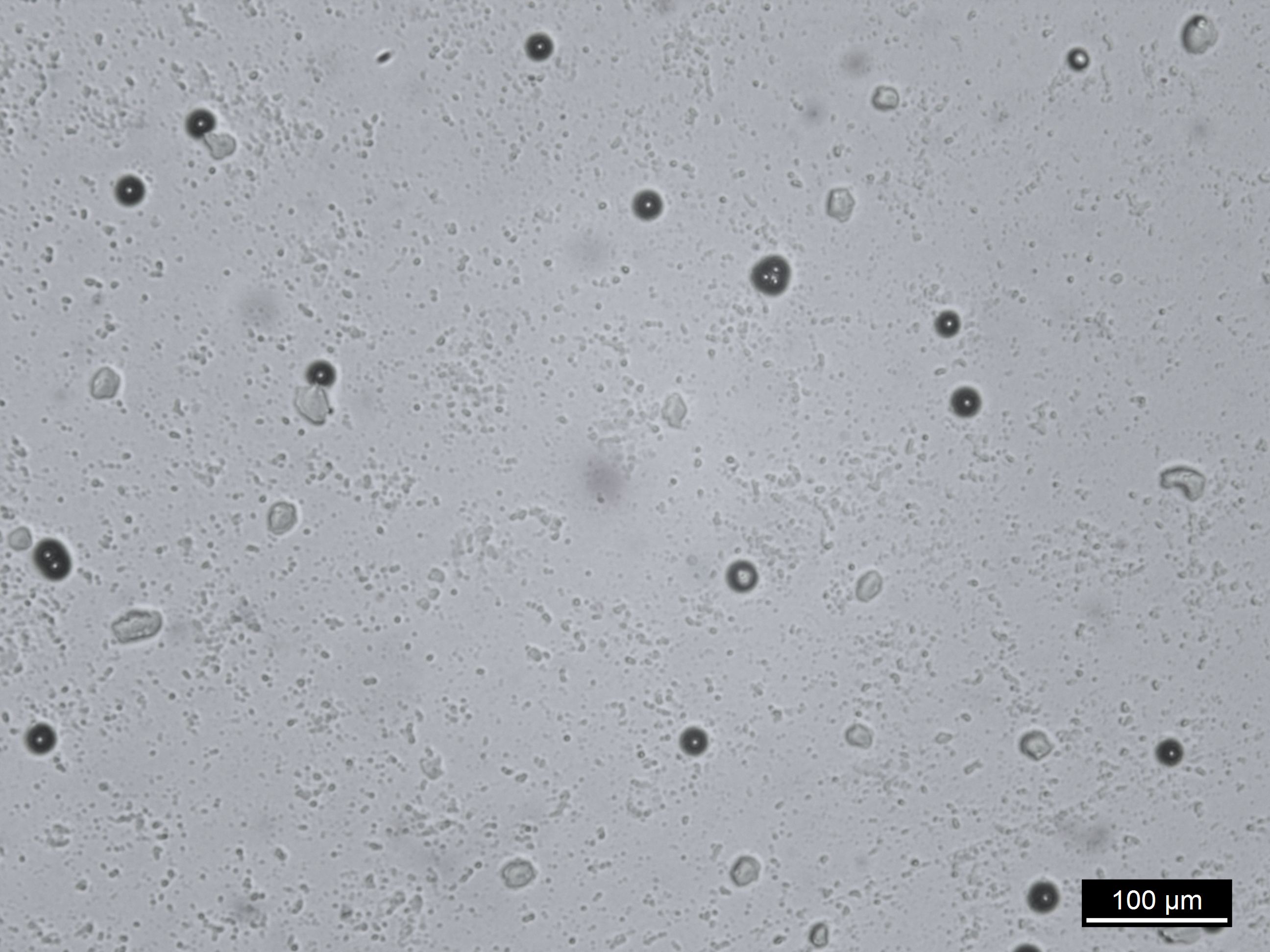

Supplement: Supplementary file 1 [file microorganisms-10-01642-s001.zip › S76_3ST_MF_P.jpg]

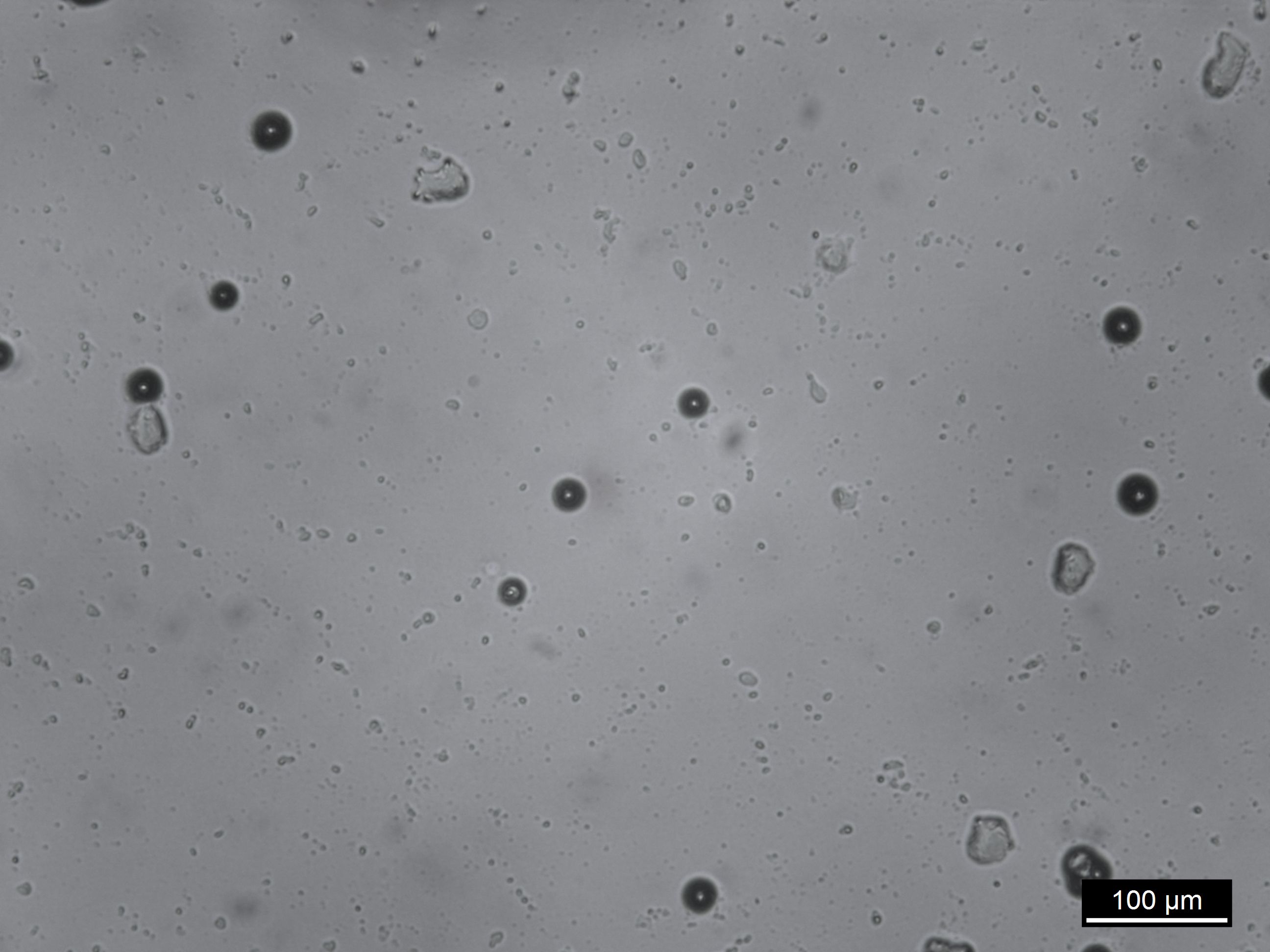

Supplement: Supplementary file 1 [file microorganisms-10-01642-s001.zip › S77_9GU_MF_C.jpg]

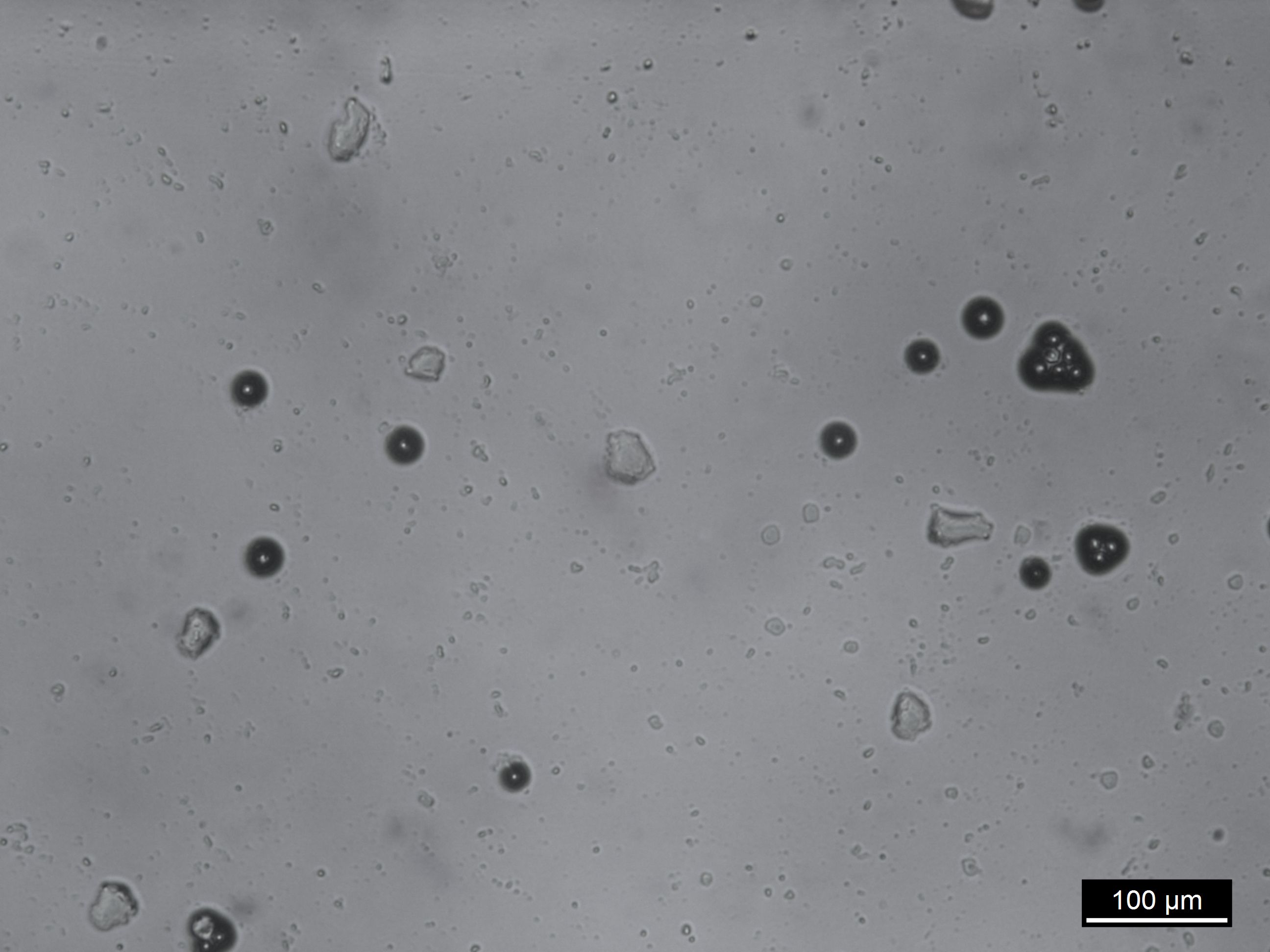

Supplement: Supplementary file 1 [file microorganisms-10-01642-s001.zip › S78_9GU_MF_P.jpg]

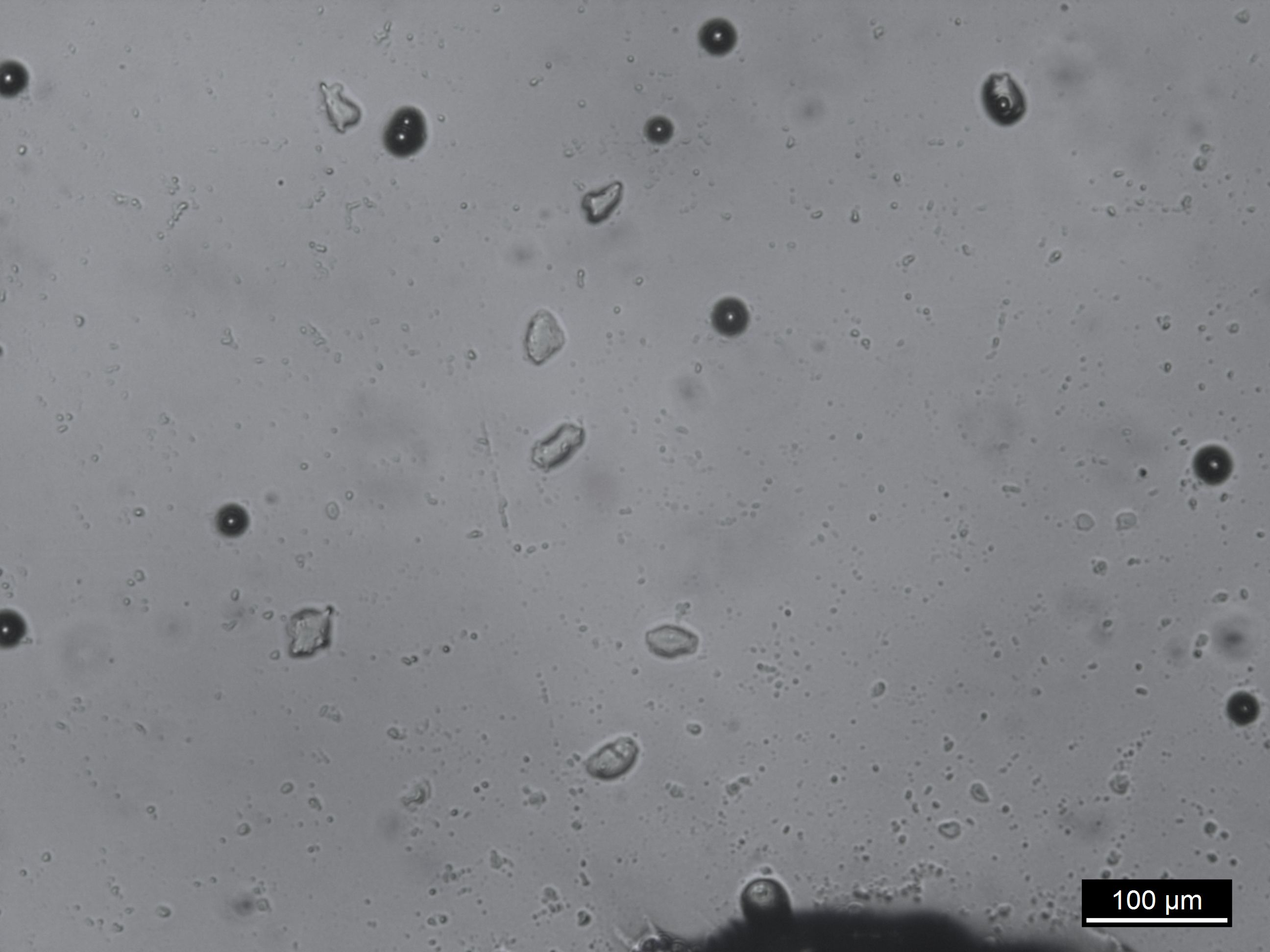

Supplement: Supplementary file 1 [file microorganisms-10-01642-s001.zip › S79_11DS_MF_C.jpg]

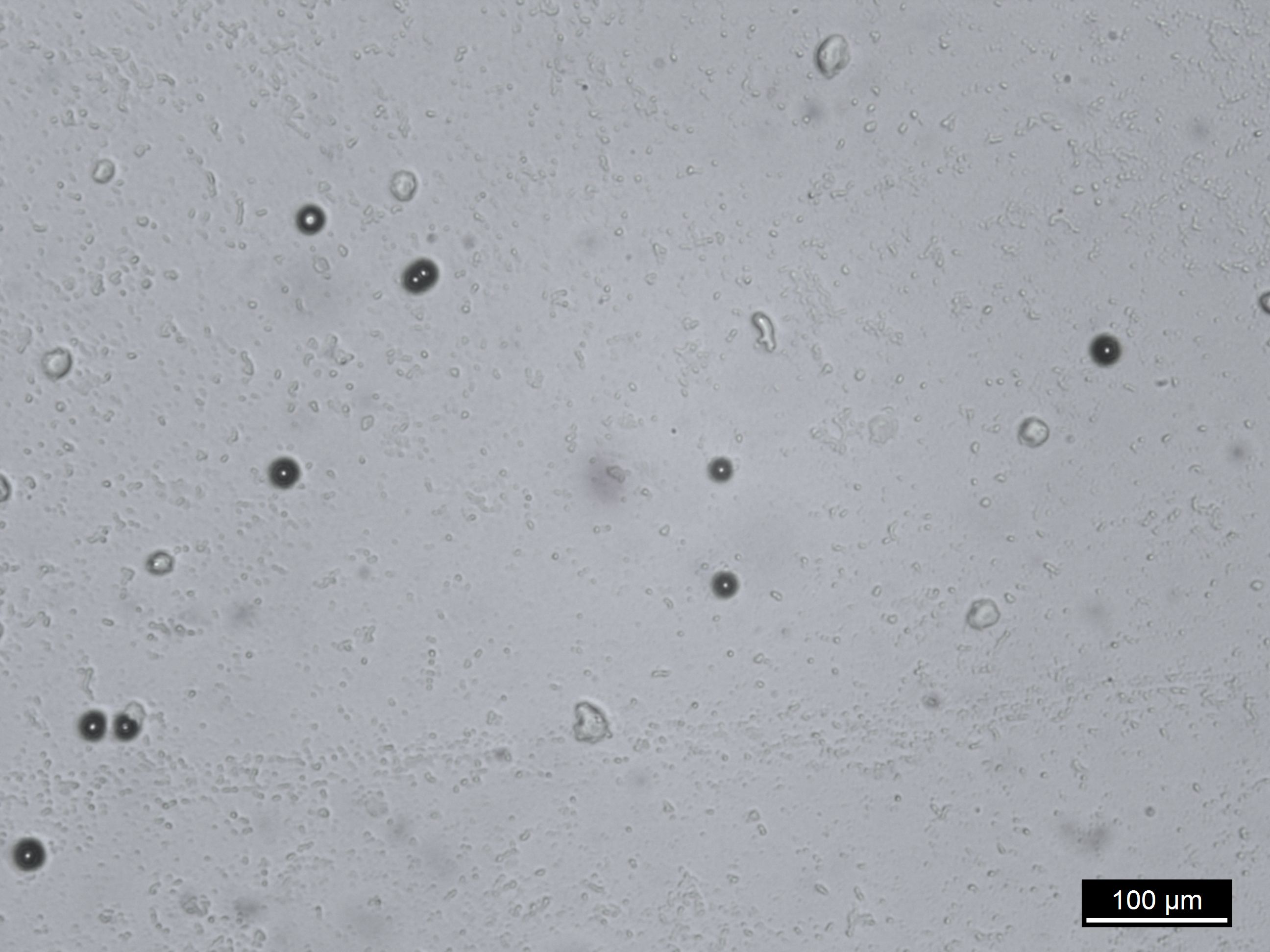

Supplement: Supplementary file 1 [file microorganisms-10-01642-s001.zip › S7_11DS_Control_C.jpg]

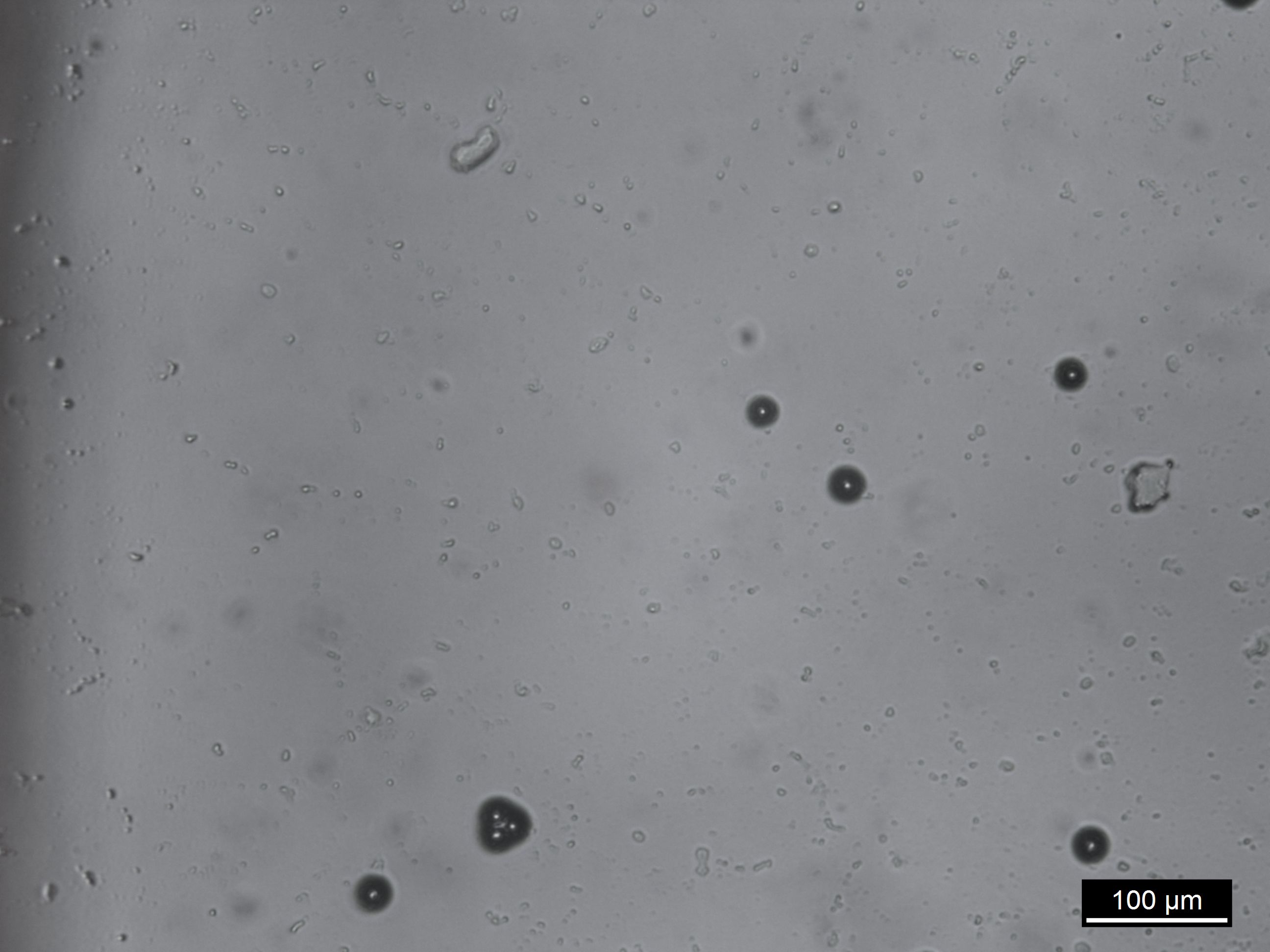

Supplement: Supplementary file 1 [file microorganisms-10-01642-s001.zip › S80_11DS_MF_P.jpg]

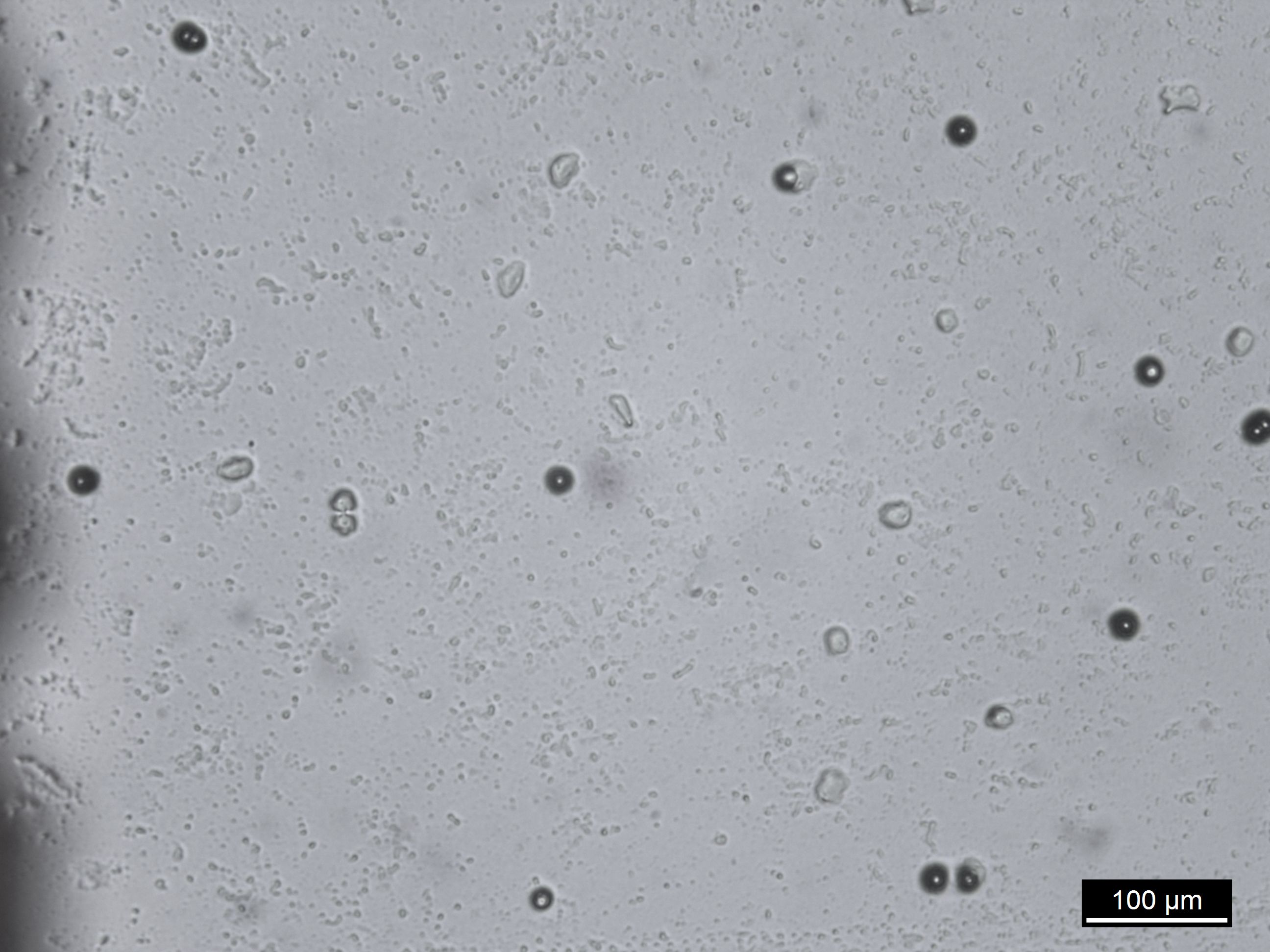

Supplement: Supplementary file 1 [file microorganisms-10-01642-s001.zip › S8_11DS_Control_P.jpg]

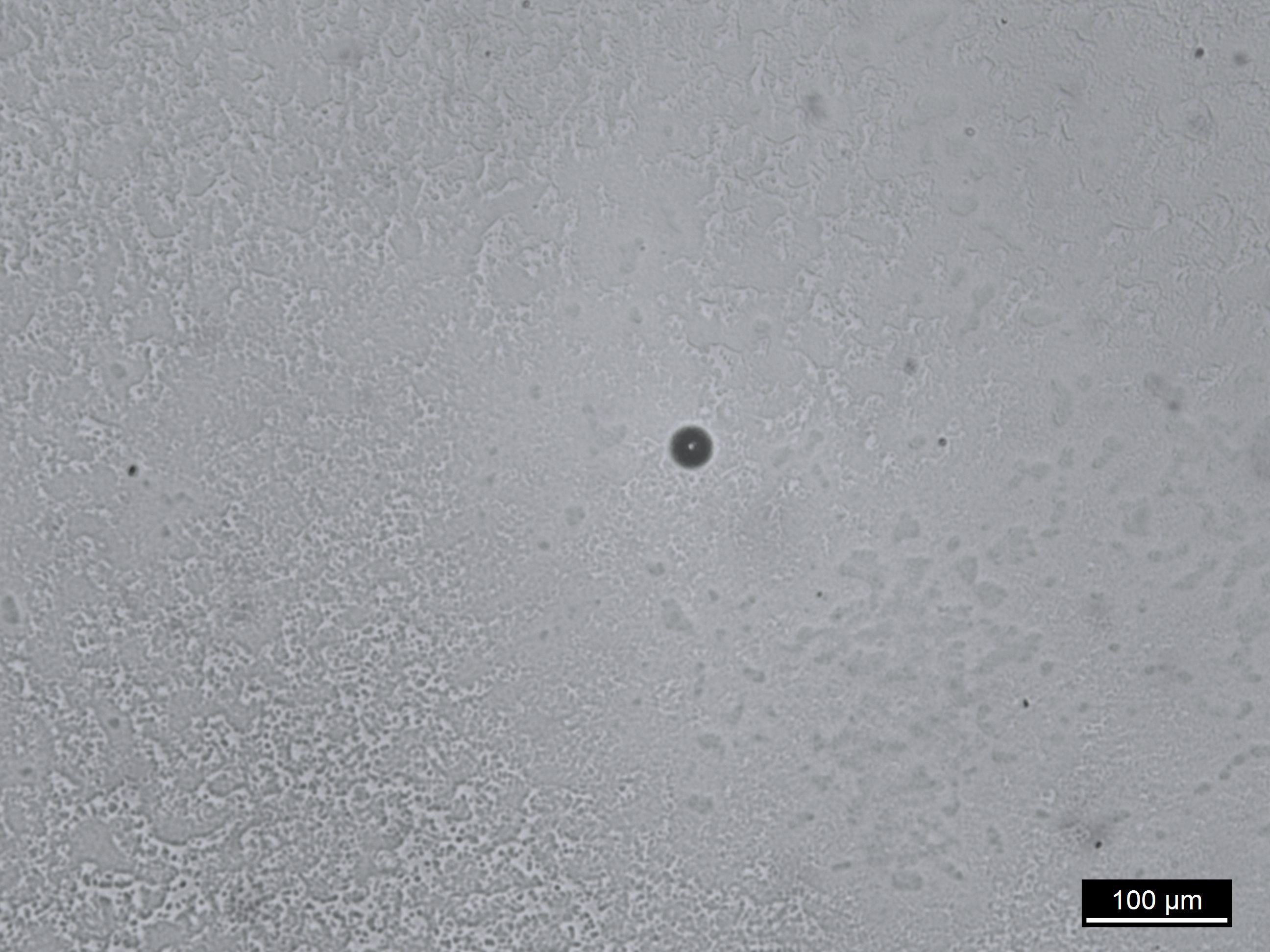

Supplement: Supplementary file 1 [file microorganisms-10-01642-s001.zip › S9_IBU_Lysoform_C.jpg]
